# JAK/STAT in human diseases: a common axis in immunodeficiencies and hematological disorders

**DOI:** 10.3389/fimmu.2025.1669688

**Published:** 2025-12-08

**Authors:** Reda Djidjik, Lydia Lamara Mahammed, Lilya Meriem Berkani, Ines Allam, Alaa Hamidou Benmoussa, Merzak Gharnaout, Brahim Belaid

**Affiliations:** 1 Department of Medical Immunology, Beni Messous University Hospital Center, Algiers, Algeria; 2 Faculty of Pharmacy, the University of Health Sciences, Algiers, Algeria; 3 Department of Pulmonology, Beni Messous University Hospital Center, Algiers, Algeria; 4 Faculty of Medicine, the University of Health Sciences, Algiers, Algeria

**Keywords:** cancers, inborn errors of immunity, JAK, mutations, signaling pathway, STAT

## Abstract

The JAK-STAT signaling pathway (Janus Kinase-Signal Transducer and Activator of Transcription) is a crucial molecular cascade that regulates immune responses, cell proliferation, and hematopoiesis. Germline or somatic mutations affecting this pathway leads to a wide range of pathologies, from severe immunodeficiencies to inflammatory and autoimmune diseases, as well as hematologic malignancies. Loss-of-function mutations impair cytokine signaling and primarily result in immunodeficiency, while gain-of-function mutations cause excessive pathway activation, promoting autoimmune diseases and myeloproliferative syndromes. Advances in our understanding of the JAK-STAT pathway and its involvement in various diseases have opened new perspectives in precision medicine and targeted therapies including JAK inhibitors (JAKi), gene therapy, and hematopoietic stem cell transplantation (HSCT). A detailed understanding of specific mutations and their effects on intracellular signaling allows for the refinement of therapeutic strategies and optimization of patient management. In this review, we examined the biology of the JAK-STAT pathway, highlighted the key pathogenic mutations and their clinical consequences, and described the laboratory and diagnostic approaches used to investigate this pathway.

## 1 Introduction

The Janus kinase-signal transducer and activator of transcription (JAK-STAT) signaling pathway plays a pivotal role in mediating signals from a broad range of cytokines and growth factors. Through this activation, it orchestrates essential biological processes such as cell proliferation, differentiation, survival, and apoptosis (**Table 1**), thereby maintaining immune homeostasis and enabling effective responses to pathogenic threats (1–9).

**Table 1:** Canonical Cytokine–JAK–STAT interactions and their physiologic effects.

Functional classification	Cytokine	JAK	STAT	Biological effect
Regulation of the Immune Response	IL2	JAK1 JAK3	STAT5	Proliferation and differentiation of T and B lymphocytes
	IL4	JAK1 JAK3	STAT6	Differentiation of CD4 T cells into Th2, activation of B lymphocytes
	IL10	JAK1 TYK2	STAT3	Inhibition of pro-inflammatory cytokine production
	IL-19	JAK1 TYK2	STAT3	Anti-inflammatory role, enhancement of Th2 response
	IL-20	JAK1 TYK2	STAT3	Regulation of keratinocyte proliferation, skin inflammation
	IL21	JAK1 JAK3	STAT3	Modulation of B, T, and NK cell responses
Inflammatory Response	IL6	JAK1 JAK2 TYK2	STAT1 STAT3	Induction of acute-phase proteins, lymphocyte activation
	IL-12	JAK2 TYK2	STAT4	Induction of Th1 differentiation, IFN-γ production
	IL17	JAK1 JAK2	STAT3	Induction of pro-inflammatory cytokine production
	IL23	JAK2 TYK2	STAT3	Differentiation of CD4 T cells into Th17
	IL-24	JAK1 TYK2	STAT3	Anti-tumor activity, apoptosis induction
	Il-26	JAK1 TYK2	STAT1 STAT3	Pro-inflammatory effects on epithelial cells
	IFN-γ	JAK1 JAK2	STAT1	Macrophage activation, increased MHC expression
	IL-13	JAK1	STAT6	Mucus production, airway remodeling and allergic inflammation
Antiviral Response	IFN-α, IFN-β	JAK1 TYK2	STAT1 STAT2	Induction of antiviral genes, inhibition of viral replication
Development and Maturation of Immune Cells	IL-3	JAK2	STAT5	Promotion of hematopoietic progenitor survival and differentiation
	IL-7	JAK1 JAK3	STAT5	Survival and proliferation of B and T cell precursors
	IL-9	JAK1 JAK3	STAT5	Stimulation of mast cell proliferation
	IL-15	JAK1 JAK3	STAT5	Maturation and survival of NK cells
Tissue Protection and Repair	IL-11	JAK1 JAK2 TYK2	STAT3	Stimulation of platelet production
	IL-22	JAK1 TYK2	STAT3	Maintenance of epithelial barrier integrity
Hematopoiesis	EPO	JAK2	STAT5	Stimulation of red blood cell production
	TPO	JAK2	STAT5	Regulation of platelet production
	GM-CSF	JAK2	STAT5	Stimulation of granulocyte and macrophage production
	G-CSF	JAK2	STAT3	Stimulation of neutrophil production
Growth and Metabolism	GH	JAK2	STAT5	Stimulation of growth and metabolism
	Leptin	JAK2	STAT3	Control of appetite and metabolism

these interactions may vary depending on the cellular context and cell type.

Tight regulation of the JAK-STAT pathway is crucial for ensuring a delicate balance between immune activation and the maintenance of self-tolerance. Disruption of this equilibrium by germline or somatic mutations in JAK or STAT genes gives rise to a broad spectrum of clinical phenotypes. Notably, recent findings have challenged the traditional view that loss-of-function (LOF) variants cause only immunodeficiency while gain-of-function (GOF) variants lead solely to inflammation and/or autoimmunity and/or malignancy (10, 11). Instead, both types of mutations can manifest with overlapping and sometimes paradoxical combinations of infection susceptibility and immune dysregulation (12, 13).

The identification and characterization of inborn errors of immunity (IEIs) affecting the JAK-STAT pathway have significantly advanced both patient care and our understanding of fundamental human immunobiology. While most germline IEIs are either inherited or result from *de novo* mutations, emerging evidence shows that post-zygotic somatic mutations restricted to a subset of cells can phenotypically mimic germline IEIs. These so-called “phenocopies” challenge traditional classifications and highlight the importance of somatic mosaicism in immune disorders (14, 15).

In parallel, mutations affecting the JAK-STAT pathway play a key role in the development of hematologic malignancies by driving persistent cytokine signaling that promotes proliferation and survival. GOF mutations in JAKs (JAK1, JAK2, JAK3) and STATs (particularly STAT3 and STAT5B), as well as alterations in cytokine receptors (IL7R, CRLF2) and negative regulators (SOCS1, PTPN2), are recurrent in myeloproliferative neoplasms and certain lymphoid cancers. These mutations not only contribute to oncogenesis but also influence prognosis (16–18).

These molecular insights have direct clinical implications. Recent progress in next-generation sequencing has greatly improved the detection of pathogenic mutations in the JAK-STAT pathway, enabling precise molecular diagnosis. This advancement facilitates the implementation of personalized and targeted therapeutic strategies, including the use of JAK inhibitors (JAKi), which have shown efficacy in attenuating aberrant signaling and are already used in the management of certain autoimmune diseases, as well as myelofibrosis and polycythemia vera. Other promising approaches include gene therapy and hematopoietic stem cell transplantation, which offer curative potential for patients with severe immunodeficiencies or LOF-associated syndromes (19, 20).

Given the rapidly evolving landscape of JAK-STAT-related research, an updated synthesis is essential. In this review, we provide a comprehensive overview of key germline and somatic mutations affecting this pathway, covering both IEIs and hematologic malignancies, two fields that are usually addressed separately, and discuss them within a unified JAK-STAT framework. To enrich the clinical relevance of this review, we have included a dedicated section on diagnostic strategies and tools, outlining current laboratory approaches and their direct clinical applications, bridging the gap between molecular insights and real-world practice.

## 2 JAK/STAT pathway

### 2.1 JAK/STAT biology

The JAK family, which includes JAK1, JAK2, JAK3, and TYK2, consists of cytoplasmic tyrosine kinases that associate with various membrane receptors to mediate intracellular signal transmission (21, 22). JAK1, JAK2, and TYK2 are expressed throughout all tissues, while JAK3 is primarily found in hematopoietic cells, where it regulates immune cell growth, survival, and differentiation (23–25).

**Figure 1:**
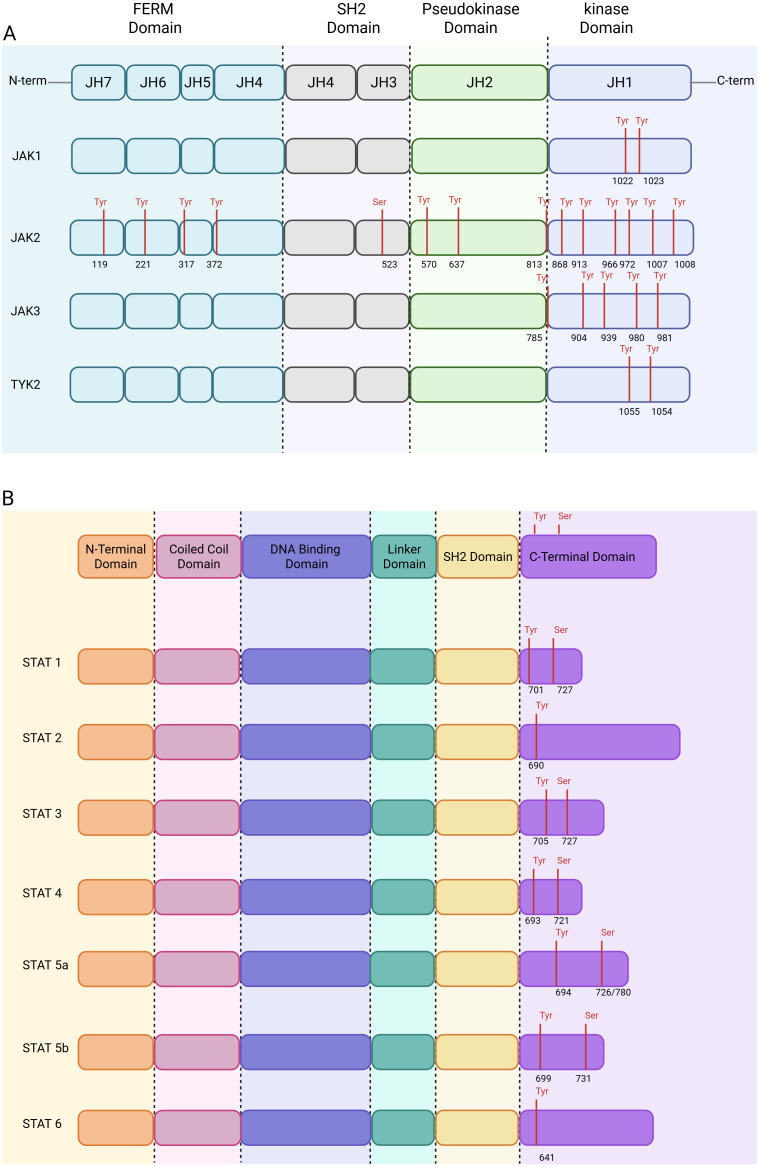
Functional domains and phosphorylation sites of JAK and STAT proteins in the JAK/STAT pathway. **(a)** Linear structure of the four Janus kinases (JAK1, JAK2, JAK3, TYK2), showing the different functional domains: FERM domain (JH7–JH4), SH2 domain (JH3–JH4), pseudokinase domain (JH2), and catalytic kinase domain (JH1). The main phosphorylation residues identified are indicated in red, including tyrosines (Tyr) and serines (Ser) critical for activation and regulation of enzymatic activity. **(b)** Schematic representation of the structures of STAT transcription factors (STAT1 to STAT6), including the N-terminal, coiled-coil, DNA-binding, linker, SH2, and C-terminal domains. Key phosphorylation sites (Tyr and Ser) are also annotated in red, as these post-translational modifications are essential for dimerization, nuclear translocation, and transcriptional activation. Created in BioRender. djidjik, r (2025). https://BioRender.com/s9fko80.

JAK proteins contain seven homology domains known as Janus Homology (JH) domains (**Figure 1**). The JH1 domain, located at the C-terminal end, corresponds to the tyrosine kinase domain, which serves as the main site of autophosphorylation. In JAK3, phosphorylation of tyrosine Y980 activates the enzyme, whereas Y981 has an inhibitory effect (26). In JAK2, the mutation of Y1007 to phenylalanine blocks activation, whereas substitution of Y1008 does not affect its function (27). Tyrosines Y1054 and Y1055 in TYK2 are also essential for ligand-induced activation (28). In JAK1, Y1023 is more highly phosphorylated than Y1022, although the precise roles of these tyrosines remain unclear (29). Additional phosphorylation sites have also been identified in JAK3, such as Y904 and Y939, which enhance its activity during cytokine-induced activation (30). In JAK2, Y868, Y966, and Y972 are required for optimal activation, whereas phosphorylation of Y913 negatively regulates its activity (31, 32). Phosphorylation of Y813, located between the JH1 and JH2 domains in JAK2, promotes binding to the adaptor SH2-Bb, further enhancing enzymatic activity (33). The equivalent tyrosine in JAK3, Y785, is also phosphorylated in response to IL-2 and is essential for this interaction (34). The JH2 domain, known as the pseudokinase domain, is catalytically inactive but regulates the enzyme’s activity. In JAK3, Y570 and Y637 are autophosphorylation sites that differentially modulate catalytic function (34, 35). The SH2 domain (JH3–JH4) has an unclear function, but phosphorylation of JAK2 at S523 within this region has been shown to act as a negative regulatory mechanism (36). Furthermore, JAK3 is also phosphorylated on serine after IL-2 stimulation (37). Finally, the N-terminal region (JH4–JH7) contains a FERM domain (band 4.1, ezrin, radixin, moesin), which is essential for interaction with cytokine receptors and regulation of kinase activity (38, 39). In JAK2, tyrosines Y119 and Y221 within the FERM domain are autophosphorylation sites with inhibitory effects (34, 40). Other sites, such as Y317(in JH5) and Y372 (in JH4), also modulate catalytic activity differently (35, 41).

The STAT protein family includes seven members (STAT1, STAT2, STAT3, STAT4, STAT5A, STAT5B, STAT6) (42, 43), each comprising approximately 750–900 amino acids (44) and sharing six conserved domains essential for their functions (45). The N-terminal domain (NTD) promotes dimerization by mediating STAT-STAT interactions, even without phosphorylation (46, 47). The coiled-coil domain (CCD) enables interaction with coactivators (e.g., IRF9, Nmi) and corepressors (e.g., SMRT/N-CoR) and plays a role in nuclear import via an NLS motif (48–50). The DNA-binding domain (DBD), characterized by an immunoglobulin-like fold, binds specific DNA motifs, such as GAS (TTCN_3_–_4_GAA) in STAT-responsive gene promoters (45, 51–53). The linker domain (LD) supports STAT structure during activation and DNA binding and aids in the formation of transcriptional complexes (45, 54). Lastly, the highly conserved SH2 domain mediates phospho-dependent protein-protein interactions essential for STAT signaling.

### 2.2 JAK/STAT signaling pathway

**Figure 2:**
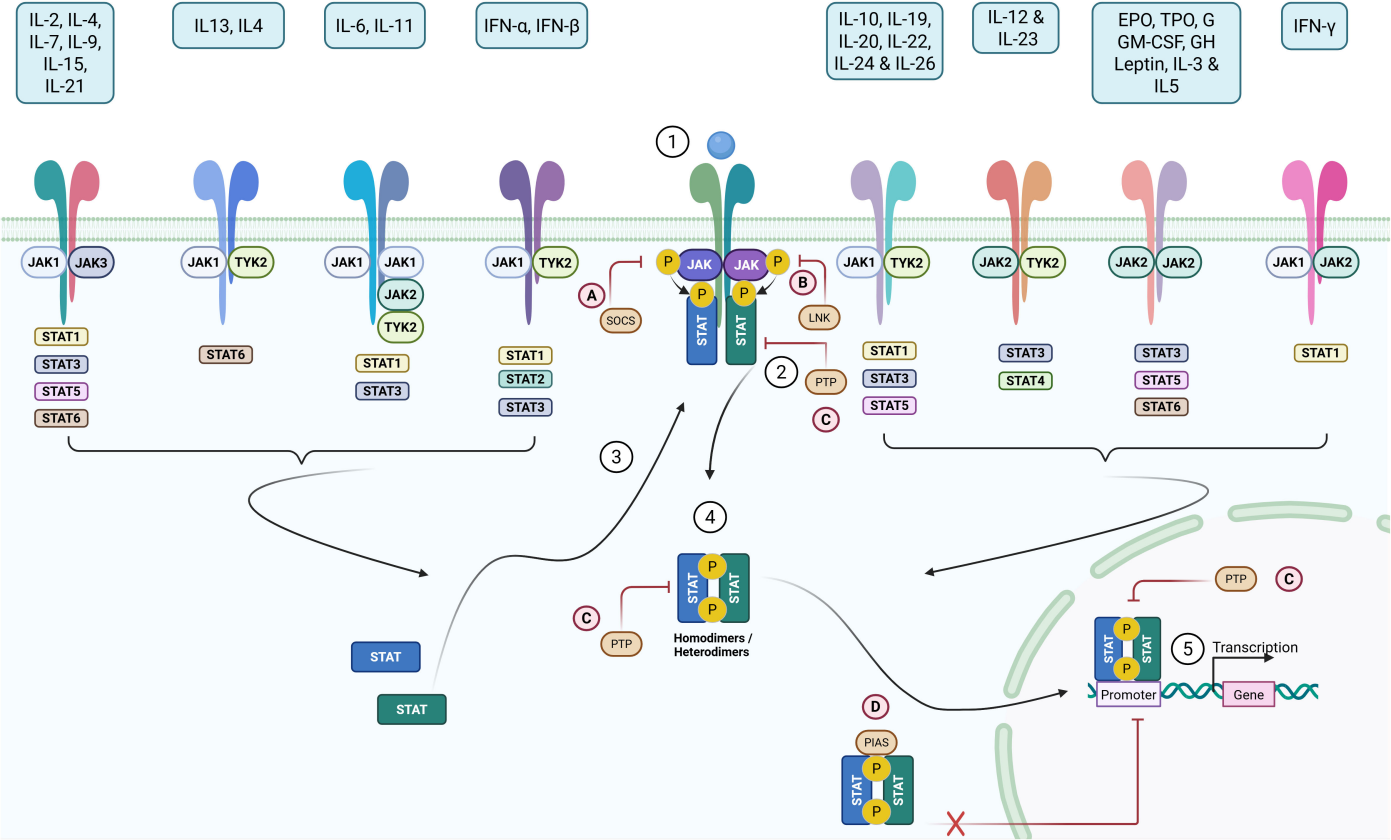
Activation and regulation of the JAK-STAT signaling pathway. The JAK-STAT signaling pathway is a key mechanism of cellular communication, activated by a variety of proteins, including hematopoietic and immune cytokines, metabolic and growth hormones, and hematopoietic growth factors. When a cytokine binds to its specific membrane receptor, (1) it triggers the activation of associated JAK proteins (JAK1, JAK2, JAK3, TYK2) through a transphosphorylation mechanism. These activated JAKs then phosphorylate tyrosine residues in the cytoplasmic domains of the receptors, (2) thereby promoting the recruitment of STAT proteins (STAT1 to STAT6). (3) These proteins, once phosphorylated, form dimers (homodimers or heterodimers) through their SH2 domains (4) and then migrate to the nucleus, where they regulate the transcription of target genes involved in various biological processes (5), such as the immune response or cell proliferation. The JAK/STAT pathway is tightly controlled by several negative regulators to prevent excessive activation. **(A)** Suppressors of Cytokine Signaling (SOCS) proteins bind, via their SH2 domain, to phosphorylated tyrosine residues of JAKs, thereby inhibiting their kinase activity and STAT recruitment. **(B)** The LNK (SH2B3) protein binds directly to phosphorylated JAK via its SH2 domain, inhibiting STAT phosphorylation. **(C)** Protein tyrosine phosphatases (PTPs) dephosphorylate JAKs and STATs at various cellular levels, while protein inhibitors of activated STATs (PIASs) **(D)** prevent the binding of dimerized STATs to DNA, thereby modulating their transcriptional effect. Created in BioRender. djidjik, r (2025). https://BioRender.com/ygo2b82.

The JAK/STAT canonical signaling pathway is initiated when a cytokine or hormone binds to its specific transmembrane receptor expressed on the surface of a target cell (**Figure 2**). This interaction induces receptor dimerization or conformational rearrangement, facilitating the juxtaposition and activation of receptor-associated Janus kinases (JAKs). The four members of the JAK family JAK1, JAK2, JAK3, and TYK2 become activated through transphosphorylation and subsequently phosphorylate specific tyrosine residues within the cytoplasmic domains of the receptor. These phosphotyrosine motifs serve as docking sites for cytoplasmic proteins of the STAT family, specifically STAT1 through STAT6, which are recruited via their SH2 domains (13, 55–57).

Following recruitment, STAT proteins are phosphorylated by the activated JAKs, leading to their dimerization into homo- or heterodimers. These dimers translocate into the nucleus, where they bind to specific DNA response elements in the promoters of target genes, ultimately modulating their transcription. The nature of the transcriptional response is determined by the specific STAT isoforms involved and the cellular context (56).

A wide variety of extracellular signals converge on this pathway. Cytokines of the common gamma-chain family, such as IL-2, IL-4, IL-7, IL-9, IL-15, and IL-21, typically activate JAK1 in association with JAK3 and primarily signal through STAT5 and STAT3, orchestrating key events in lymphocyte development, proliferation, and survival. The IL-6 family of cytokines, including IL-6 and IL-11, generally signal through JAK1 in combination with either JAK2 or TYK2 and engage STAT3, playing critical roles in inflammation and acute-phase responses (6).

Type I interferons (IFN-α, IFN-β) signal through receptors associated with JAK1 and TYK2, leading to the activation of STAT1 and STAT2, and promoting antiviral responses and immune surveillance. In contrast, Type II interferon (IFN-γ) signals through JAK1 and JAK2, primarily activating STAT1 to induce macrophage activation, antigen presentation, and pro-inflammatory gene expression (58).

Interleukins such as IL-10, IL-19, IL-20, IL-22, and IL-24 also use JAK1 and TYK2 to engage STAT3, thereby exerting anti-inflammatory and barrier-protective functions, particularly at mucosal surfaces. The IL-12 and IL-23 families activate JAK2 and TYK2, triggering STAT4 and STAT3, respectively, which are essential for the differentiation of Th1 and Th17 cells. Finally, a group of hematopoietic growth factors and metabolic hormones, including erythropoietin (EPO), thrombopoietin (TPO), GM-CSF, growth hormone (GH), leptin, IL-3, and IL-5, utilize JAK2 homodimers to signal primarily via STAT5, regulating erythropoiesis, granulopoiesis, and systemic growth and metabolism (3, 6).

Despite the diversity of ligands and biological effects, the JAK/STAT pathway operates through a common signaling architecture, allowing for a rapid and transcriptionally efficient response to extracellular cues. However, this potency also requires stringent regulatory control mechanisms to prevent aberrant or prolonged activation, which could otherwise result in immunopathology or oncogenesis.

The regulation of the JAK/STAT pathway involves multiple negative feedback loops that act at various levels of the signaling cascade. One key regulatory system is mediated by SOCS (Suppressor of Cytokine Signaling) proteins, which are themselves transcriptionally induced by STATs. SOCS proteins bind directly to phosphorylated JAKs or cytokine receptors via their SH2 domains and block further signal propagation by inhibiting kinase activity or competing with STAT recruitment. This classical negative feedback loop is essential for curtailing excessive cytokine responses and restoring homeostasis after immune stimulation (19, 59).

Another layer of control is provided by protein tyrosine phosphatases (PTPs), such as SHP1, SHP2, and TC-PTP, which can dephosphorylate JAKs and STATs at multiple steps of the signaling pathway. By reversing phosphorylation events, PTPs effectively shut down active signaling complexes, ensuring that the duration of the response is appropriately limited (59, 60).

A third class of negative regulators is the PIAS (Protein Inhibitors of Activated STATs) family, which functions in the nucleus to inhibit the transcriptional activity of STAT dimers. PIAS proteins prevent STATs from binding to DNA or promote the recruitment of co-repressors, thereby modulating gene expression without altering the phosphorylation status of STATs (61).

## 3 Impact of JAK/STAT mutations on human diseases

As mentioned, the JAK/STAT signaling pathway serves as a crucial regulatory network for various cellular processes. Disruption of normal JAK/STAT protein function, particularly through mutations, is linked to a wide range of human diseases, especially inborn errors of immunity (IEIs) and hematologic malignancies.

### 3.1 JAK/STAT pathway and IEIs

**Figure 3:**
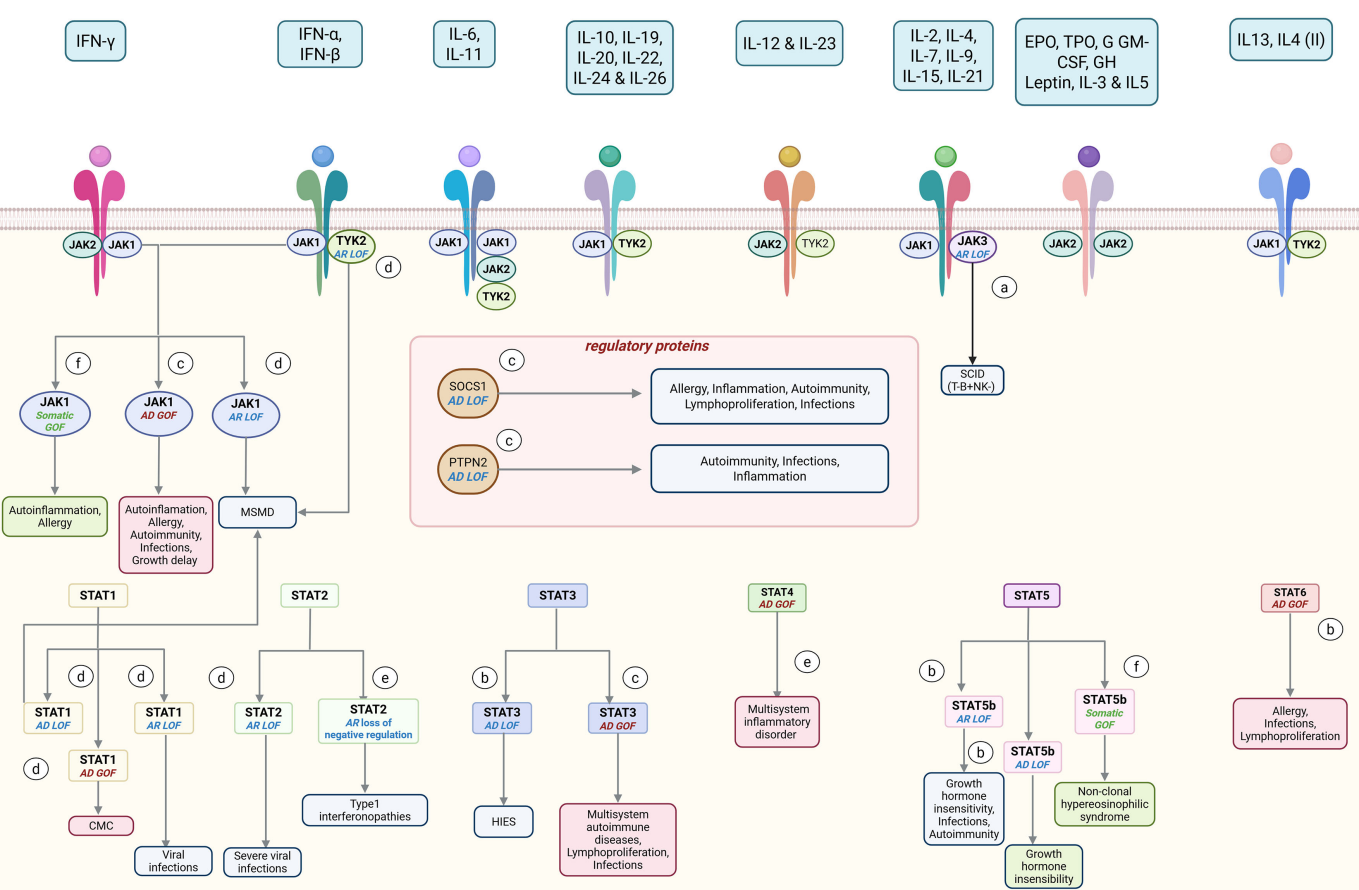
JAK-STAT mutations in inborn errors of immunity. At the molecular level, diverse dysregulations can disrupt the JAK-STAT pathway, leading to a various type of inborn errors of immunity (IEIs) including **(a)** Immunodeficiencies affecting cellular and humoral immunity, **(b)** Combined immunodeficiencies with associated or syndromic features, **(c)** Diseases of immune dysregulation, **(d)** Defects in intrinsic and innate immunity, **(e)** Autoinflammatory disorders and **(f)** Phenocopies of inborn errors of immunity. Mutations in proteins associated with the JAK-STAT pathway are presented along with the clinical manifestations of the resulting IEIs. Germline LOF defects and their principal clinical symptoms are displayed in bleu, germline GOF defects in red and somatic GOF defects in green. AD, autosomal dominant; AR, autosomal recessive; CMC, chronic mucocutaneous candidiasis; GOF, gain of function; HIES, hyper-IgE syndrome; LOF, loss of function; MSMD, Mendelian susceptibility to mycobacterial disease; SCID, severe combined immunodeficiency. Created in BioRender. djidjik, r (2025). https://BioRender.com/4jpwstk.

According to the latest International Union of Immunological Societies (IUIS) classification in 2024 (62), nineteen IEIs have been associated with mutations in the JAK-STAT pathway (**Figure 3**, **Table 2**). Reported disorders are linked to distinct inheritance patterns (autosomal recessive (AR) or autosomal dominant (AD)) and distinct mutation types (germline or somatic, LOF or GOF).

**Table 2:** Overview of IEIs in JAK-STAT signaling pathway (12, 13).

Molecule		Type of mutation	Inheritance	Penetrance	Predominant phenotype	Therapy/management
STATs	STAT1	Germline, LOF	AR	Complete	Viral and mycobacterial infections	HSCT [*complete deficiency*] Antimicrobial agents [*partial deficiency*]
			AD	Incomplete	Mycobacterial infections	Antimicrobial agents
		Germline, GOF	AD	Complete	Chronic mucocutaneous candidiasis	JAKi (+ anti-viral prophylaxis again herpesviruses) HSCT [*to consider for patients with severe and intractable symptoms*]
	STAT2	Germline, LOF	AR	Incomplete	Viral infections	Antiviral agents and immunoglobulin supplementation (+live viral vaccine avoidance)
		Loss of Negative Regulation	AR	Complete	Autoinflammation	JAKi
	STAT3	LOF	AD	Complete	Hyper IgE syndrome	Antimicrobial agents HSCT [*to consider for patients with severe and intractable symptoms*]
		Germline, GOF	AD	Incomplete	Multisystem autoimmunity, infections, inflammation, lymphoproliferation	JAKi and IL-6 blockade (i.e., tocilizumab)
	STAT4	Germline, GOF	AD	Complete	Systemic inflammatory disorder (disabling pansclerotic morphea)	JAKi
	STAT5B	Germline, LOF	AR	Incomplete	Growth failure, infections, autoimmunity	Supportive (IGF-1 replacement, infection prophylaxis)
			AD	Incomplete	Impaired post-natal growth	Supportive (IGF-1 replacement)
		Somatic, GOF	/	/	Non-clonal hypereosinophilic syndrome (urticaria, dermatitis, diarrhea)	JAKi
	STAT6	Germline, GOF	AD	Complete	Atopic dermatitis, allergy, elevated serum IgE	JAKi and dupilumab
JAKs	JAK1	Germline, LOF	AR	Complete	Mycobacterial infections	Antimicrobial agents
		Germline, GOF	AD	Incomplete	Multiorgan immune dysregulation	JAKi
		Somatic, GOF	/	/	Autoinflammation, allergy	JAKi
	JAK3	Germline, LOF	AR	Complete	Severe combined immunodeficiency	Supportive therapy until HSCT
	TYK2	Germline, LOF	AR	Incomplete	Mycobacterial infections	Antimicrobial agents
Regulators	SOCS1	Germline, LOF	AD	Incomplete	Allergy, inflammation, autoimmunity, lymphoproliferation, infections	JAKi
	PTPN2	Germline, LOF	AD	Incomplete	Autoimmunity, infections, inflammation	Consider JAKi

AD, Autosomal Dominant; AR, Autosomal Recessive; GOF, Gain Of Function; HSCT, Hematopoietic Stem Cell Transplantation; JAKi, JAK inhibitors; LOF, Lost Of Function.

**Figure 4:**
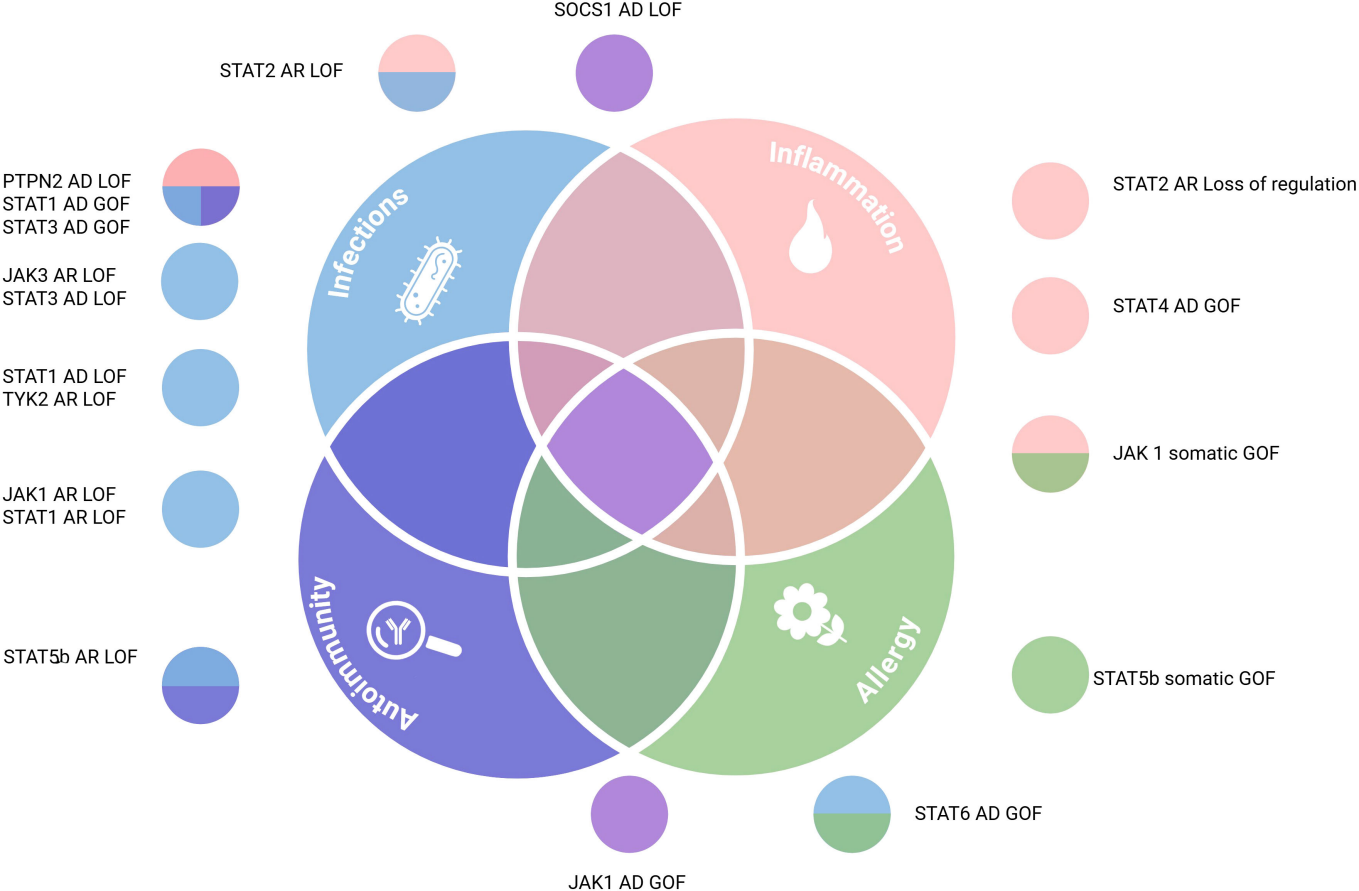
Clinical overlap of JAK-STAT pathway associated IEIs. Some IEIs are characterized by predominant manifestations within one of four distinct pathological axes: infections (light blue), autoimmunity (purple), inflammation (pink), or allergy (green), while others exhibit overlapping symptoms. AD, autosomal dominant; AR, autosomal recessive; GOF, gain of function; LOF, loss of function. Created in BioRender. djidjik, r (2025). https://BioRender.com/72xxu1g.

These mutations give rise to a broad spectrum of overlapping clinical phenotypes (14), spanning from infection susceptibility to autoimmune and inflammatory manifestations (**Figure 4**, **supplementary Tables S1 to S10**). The clinical picture is largely shaped by the nature of the variant: LOF and dominant negative (DN) mutations both impair cytokine signaling and compromise immune responses, resulting in severe or atypical immunodeficiencies. While LOF mutations result in a non-functional or absent protein, DN mutations produce aberrant proteins that interfere with the function of the wild-type counterpart, often leading to even more profound signaling defects. In contrast, GOF mutations promote hyperactivation of the pathway and are associated with chronic inflammatory diseases, autoimmune disorders, and hematologic malignancies (10, 11). However, this simple dichotomy no longer captures the clinical reality of JAK-STAT disorders. Patient cohorts and recent mechanistic studies have revealed that both LOF and GOF mutations can generate phenotypes that blur the classical boundaries between immunodeficiency and inflammation/autoimmunity. In practice, patients with activating mutations may also suffer from recurrent or opportunistic infections, while some with inactivating variants develop autoimmune or autoinflammatory features (12, 13). These apparently contradictory presentations highlight that impaired signaling does not operate in a single direction but rather perturbs a wider immunological network.

To enhance the understanding of these immunodeficiencies, they will be presented in the following sections according to the most recent IUIS classification.

#### 3.1.1 Immunodeficiencies affecting cellular and humoral immunity

• ***AR JAK3 LOF***

Biallelic null mutations (homozygous/compound) in the *JAK3* gene cause SCID (63, 64). These mutations result in a total or near-total loss of JAK3 expression and function, preventing the signaling of all cytokines that rely on the common γ chain (CD132). At the molecular level, JAK3 associates with the common γ chain and is essential for signal transduction by γc-containing receptors, including IL7 and IL15, which are required for the development of T and NK cells, respectively (65). Consequently, mutations in human JAK3 lead to a complete absence of circulating T and NK cells, with a normal number of nonfunctional B cells (T^-^B^+^NK^-^ SCID). The clinical phenotype of JAK3-SCID is nearly identical to that of X-linked SCID (IL2Rγ deficiency). Affected patients typically present within the first few months of age with oral candidiasis (thrush), intractable diarrhea with failure to thrive, and life-threatening bacterial, viral, and fungal infections, often caused by *Pneumocystis jirovecii* (66–74).

Hypomorphic forms of *JAK3* have been identified in patients with late-onset combined immune deficiency (75, 76). Some hypomorphic mutations result in residual protein function allowing partial cytokine signaling and enabling some production of T and NK cells. These patients exhibit a milder clinical phenotype, characterized by less severe infections and the presence of autoimmune features (type 1 diabetes, autoimmune cytopenia).

#### 3.1.2 Combined immunodeficiencies with associated or syndromic features

• ***AD STAT3 LOF (dominant negative)***

Dominant negative mutations in *STAT3* are the most common cause of hyper IgE syndrome (HIES), previously known as Job’s syndrome (77). This multisystem disorder is characterized by eczematoid dermatitis, recurrent skin and lung infections, retained primary teeth, skeletal and joint abnormalities, and vascular anomalies (78). The multisystemic manifestations of the disease are consistent with the ubiquitous expression of STAT3 and its crucial role in mediating responses to various cytokines and growth factors, including the IL-6 family, IL-10 family, IL-21, and IL-23 (79). The infectious spectrum is predominantly characterized by bacterial and fungal infections, primarily involving *Staphylococcus aureus* (80), *Candida albicans* (81), and *Aspergillus* (82, 83), likely related to defective STAT3/Th17 immunity (84).

• ***AD STAT6 GOF***

Recently, several research groups reported a new primary atopic disease caused by germline heterozygous *STAT6* GOF variants (85–89). These mutations are primarily located in the DNA-binding domain, enhancing the transcriptional activity of STAT6. An increase in STAT6 phosphorylation was observed before and after IL-4 stimulation (86, 87), along with the overactivation of STAT6 target genes and excessive expression of IL4R (87, 89), CCL24 (85), and IL-5, leading to elevated production of Th2 cytokines (IL-4, IL-5, IL-13) (87). Consistent with STAT6’s physiological role, elevated serum IgE levels and marked hypereosinophilia were prominent characteristics observed in STAT6 GOF patients.

Clinically, this condition is a multisystem disorder characterized by severe early-onset treatment-resistant atopic dermatitis, multiple food and drug allergies, eosinophilic gastrointestinal disease, lymphoproliferation, and some syndromic features (85–89). Moreover, recurrent skin and respiratory infections of bacterial, fungal, and viral origin have been reported (87, 88). In one case, B-cell lymphoma was described (89). Several features of STAT6 GOF overlap with those of autosomal dominant HIES, justifying its classification as another cause of HIES (62).

• ***AR STAT5b LOF***

Patients with homozygous LOF *STAT5B* mutations (90–98) exhibit a combination of severe growth hormone insensitivity and significant immunodeficiency.

As the primary downstream mediator of the IL-2 cytokine family (including IL-2, IL-4, IL- 7, IL-9, and IL-15), STAT5B plays a crucial role in lymphocyte development, function, and survival (99). Consequently, STAT5B mutations can lead to T and NK lymphopenia (93, 95, 97), potentially explaining the increased susceptibility to severe viral and bacterial infections. Additionally, STAT5B is also crucial for the differentiation of regulatory T cells (100). Indeed, Treg count was found to be reduced in a significant proportion of patients with homozygous STAT5B mutations, who presented with autoimmune manifestations (e.g., Hashimoto’s thyroiditis, autoimmune cytopenia) (101).

These mutations disrupt growth hormone signaling, which is associated with post-natal growth failure resulting in severe short stature (90–98). Facial dysmorphism (95), delayed bone age (93, 97), and delayed puberty (92) were also noted in some cases.

• ***AD STAT5b LOF (dominant negative)***

Heterozygous dominant negative LOF *STAT5B* mutations are extremely rare. Patients present with a milder clinical phenotype characterized by partial growth hormone insensitivity, impaired post-natal growth, eczema and elevated serum IgE without the profound immunologic abnormalities seen in AR *STAT5B* LOF variants (102). At the molecular level, mutant proteins exhibit normal mobilization to the nucleus, but their DNA-binding capacity is impaired. Moreover, these variants can dimerize with wild-type STAT5B, disrupting its normal function and explaining the pathogenicity in heterozygous carriers.

#### 3.1.3 Diseases of immune dysregulation

• ***AD STAT3 GOF***

GOF mutations in *STAT3* lead to excessive activation of STAT3 signaling, promoting hyperinflammation and severe autoimmunity.

Patients with *STAT3* GOF mutations exhibit a dysregulation in the balance between Th17 and Treg cells, characterized by an expansion of Th17 cells and a decreased number of Treg cells with impaired function. This imbalance results in autoimmune manifestations, with more than half of the patients presenting with autoimmune cytopenia (103, 104). Affected individuals frequently develop hypogammaglobulinemia and lymphopenia, making them susceptible to sinopulmonary bacterial infections (103, 104).

Patients with this disorder also exhibit massive lymphoproliferation, with lymphadenopathy, and hepatosplenomegaly (103, 104). Some patients may experience growth delays likely due to enhanced STAT3 signaling disrupting STAT5B-dependent growth hormone signaling (105–107).

• ***AD JAK1 GOF***

The first germline GOF mutation in *JAK1* was reported in 2017 by Del Bel et al. (108). Since then, several patients with *JAK1* GOF variants have been identified (109–111). These mutations are primarily located in the inhibitory pseudo-kinase domain of JAK1, resulting in persistent overactivation of the JAK-STAT signaling pathways. Pronounced hyperphosphorylation of STAT1 and STAT3 has been reported both at baseline and after stimulation with IFN-γ and IL-6 (108, 111). Although less consistently observed, this dysregulation can extend to other STAT proteins, including STAT2, STAT4, STAT5B, and STAT6, leading to an overlapping clinical phenotype. Affected individuals typically present in infancy and experience a lifelong multisystem disorder with heterogeneous features, including atopic diseases (severe atopic dermatitis, allergies, asthma), hyper-eosinophilic syndrome (profound eosinophilia with eosinophilic infiltration of the liver and gastrointestinal tract), autoimmunity (autoimmune thrombocytopenia, autoimmune hepatitis, type I diabetes), inflammatory bowel disease, recurrent infections, and growth defects (108–111).

• ***SOCS1 haploinsufficiency***

Heterozygous LOF mutations in *SOCS1* were first reported in 2020 (112–114). Since then, several patients have been identified. The evolving disease phenotype includes a wide range of immune dysregulatory manifestations, such as allergy, inflammatory bowel diseases, autoimmune cytopenia, lymphoproliferation, and rheumatological manifestations (115). Patients also show increased susceptibility to bacterial and viral infections. Notably, these mutations exhibit incomplete penetrance, with some carriers remaining asymptomatic. Immunological investigations revealed a low proportion of switched memory B cells while the proportion of CD21^low^ B cells was elevated (115). The patients’ PBMC showed increased STAT1 phosphorylation and enhanced IFN I and II signaling consistent with reduced SOCS1 activity (114).

• ***PTPN2 Haploinsufficiency***

Recent studies have identified both heterozygous and homozygous LOF mutations in *PTPN2* associated with a spectrum of clinical manifestations, including autoimmune enteropathy (116, 117), recurrent infections (112), and systemic autoimmunity, including systemic lupus erythematosus and cytopenia (118). Mutations in PTPN2 also result in incomplete penetrance, similar to that observed with SOCS1 haploinsufficiency. At the molecular level, increased phosphorylation of STAT1 and STAT5 after stimulation with IFN-γ and IL-2 has been reported, reflecting hyperactivation of the JAK–STAT pathway (118). Additionally, elevated levels of inflammatory cytokines have been detected in the patients’ sera, further underscoring the inflammatory immune dysregulation associated with PTPN2 deficiency.

#### 3.1.4 Defects in intrinsic and innate immunity

• ***Mendelian Susceptibility To Mycobacterial Disease (MSMD)***

• ***AD STAT1 LOF***

Heterozygous LOF mutations in *STAT1* are rare and lead to increased susceptibility to mycobacterial infections without predisposing to viral infections (119–129), this can be explained by an impaired response to IFN-γ, while IFN-α/β signaling remains preserved. This likely stems from the distinct signaling mechanisms of interferons: IFN-γ relies on STAT1-STAT1 homodimers, whereas IFN-α/β signaling occurs through STAT1-STAT2 heterodimers (130).

• ***AR TYK2 LOF***

TYK2 deficiency is a rare disorder inherited as an AR trait, initially described as a cause of HIES (131). However, further investigation revealed that the majority of TYK2-deficient patients do not exhibit the HIES features. Instead, they demonstrate increased susceptibility to intracellular bacterial and/or viral infections, with mycobacterial disease being the most consistent infection (132). Therefore, it was proposed that human TYK2 deficiency represents a distinct IEIs entity, clinically different from the previously identified patients with AR-HIES. According to the categorization reported by IUIS, TYK2 deficiency was classified under MSMD (133).

There are three forms of TYK2 inherited deficiency (134):

- AR complete TYK2 deficiency (with or without TYK2 expression) (131, 132, 134–136): Characterized by MSMD (and more rarely tuberculosis) and/or viral diseases due to an impaired response to IL-12, IL-23 (poor IFN-γ underlies mycobacterial diseases), IFN-α (viral diseases), and IL-10 signaling.
- Homozygosity for the missense variant P1104A (137): Described as a rare genetic etiology of MSMD and a common genetic cause of primary tuberculosis. Patients mainly present tuberculosis, and more rarely MSMD, without viral diseases. This variant affects the enzymatic activity of TYK2 but not its expression leading to selectively impaired responses to IL-23.
- AR partial TYK2 deficiency (134): Caused by hypomorphic variants. Individuals with partial TYK2 deficiency have normal TYK2 expression but exhibit either impaired responses to several cytokines (similar to complete TYK2 deficiency) or selectively impaired responses to IL-23 (as seen in P1104A).

The impairment of IL-23–dependent induction of IFN-γ is the only mechanism of mycobacterial disease common to all molecular forms of TYK2 deficiency.

• ***AR JAK1 LOF***

So far, only one patient with *JAK1* germline [LOF] mutations has been identified (138), where JAK1 was reduced but not completely absent. Exome sequencing revealed two homozygous hypomorphic mutations affecting the pseudo-kinase domain of JAK1 leading to impaired phosphorylation of several STAT proteins (STAT1, STAT3, STAT4, STAT5, and STAT6), which contributed to the immunodeficiency manifested by the patient. He presented with recurrent atypical mycobacterial infections, cardiomyopathy, developmental delay, and early-onset metastatic bladder carcinoma. Susceptibility to atypical mycobacterial infections could be related to T cell lymphopenia, reduced IFN-γ production, and an impaired JAK1-STAT1 signaling pathway.

• ***Predisposition to Severe Viral Infection***

• ***AR STAT1 LOF***

STAT1 deficiency, in its autosomal recessive inheritance pattern, can occur in two forms: complete and partial.

Complete LOF leads to early-onset, life-threatening infections caused by intracellular pathogens (nontuberculous mycobacteria, BCG) and viruses (typically herpesviruses), reflecting the lack of a STAT1-mediated response to IFN-γ, IFN-α/β, IFN-λ, and IL-27 (139–144). HSCT remains the only curative option for this disease. Notably, some cases suffer from a secondary hemophagocytic syndrome (143).

A milder form of the disease (partial AR *STAT1* LOF) has been reported in patients with biallelic hypomorphic mutations, who exhibit an impaired but not abolished STAT1 response to interferons (144–147).

• ***AR STAT2 LOF***

As part of the ISGF3 (STAT1-STAT2-IRF9) transcription factor complex, STAT2 is essential for cellular responses to type I and type III interferons downstream of their respective receptors (148). Consequently, biallelic LOF mutations in *STAT2* impair the formation of the ISGF3 complex, disrupting the induction of interferon-stimulated antiviral genes (ISGs), resulting in defective IFN-α and IFN-β signaling and an inadequate ability to control viral infections.

Patients with a complete STAT2 deficiency suffer from severe adverse reactions to live attenuated viral vaccines (especially the measles-mumps-rubella vaccine) and severe viral infections, particularly critical influenza pneumonia, critical COVID-19 pneumonia, and herpes simplex virus type 1 (HSV-1) encephalitis (149). Furthermore, some patients experience hemophagocytic lymphohistiocytosis (HLH) and other inflammatory complications, including myocarditis and atypical Kawasaki disease.

• ***Predisposition To Mucocutaneous Candidiasis***

• ***AD STAT1 GOF***

Germline-activating *STAT1* mutations are the most common cause of STAT1-related IEIs. This disease is inherited in an autosomal dominant manner. Dysregulation of STAT1 due to GOF mutations leads to a disorder associated with IEIs that presents with a wide spectrum of phenotypes. These range from mild cases of recurrent thrush, which meet the definition of chronic mucocutaneous candidiasis (CMC), to severe cases that may hinder the diagnosis. In the largest multicenter study of 274 patients with *STAT1*-GOF mutations (150), it was found that 98% of the cases developed CMC. This prevalence can be partially explained by a deficiency of TH17 cells observed in 87% of these patients, which is associated with negative effect of *STAT1* GOF on STAT3-driven induction of TH17 cells crucial for preventing CMC (151). Additionally, patients were susceptible to recurrent lower respiratory tract bacterial infections, primarily caused by *Staphylococcus aureus*. Aside from infectious phenotypes, autoimmune diseases are common complications, affecting 40% of patients, along with conditions such as hypothyroidism, type I diabetes, Addison’s disease, and autoimmune thrombocytopenia. A small proportion (5%) of patients develop squamous cell cancers, particularly cutaneous carcinomas.

#### 3.1.5 Autoinflammatory Disorders

• ***AR STAT2 Loss of Negative Regulation***

In the later stages of IFN-I signaling, STAT 2 contributes to negative regulation by recruiting USP18 to the IFNAR2 subunit of the IFN-I receptor (58). To date, three homozygous *STAT2* variants (R148W, R148Q, and A219V) have been reported to impair this regulatory function. These variants disrupt USP18 binding (R148W and A219V) (152, 153) or hinder proper trafficking of the STAT2-USP18 complex to the receptor (R148Q) (154), leading to prolonged IFN-I signaling and contributing to interferonopathies.

Notably some previous reports referred to the R148W and R148Q variants as conferring a “GOF”. Nevertheless, in this context, the term “GOF” describes the resulting biochemical and clinical phenotype rather than the underlying molecular mechanism related to the loss of the negative regulatory function of STAT2 on IFN-I signaling.

Patients with homozygous loss-of-inhibitory function mutations in *STAT2* mainly present with severe inflammatory encephalopathy (including seizures, brain calcifications, and developmental delay) and inflammatory interstitial lung disease (152–154).

• ***AD STAT4 GOF***

Baghdassarian et al. identified the first monogenic disorder caused by a heterozygous germline GOF variant in STAT4 in 2023 (155). Affected patients presented with disabling pansclerotic morphea, a severe childhood-onset systemic inflammatory disorder characterized by skin sclerosis and mucosal ulcerations. Laboratory evaluations showed mild neutropenia and lymphopenia with hypogammaglobulinemia. Inhibition of JAK signaling with ruxolitinib led to the normalization of most immunologic variables and resolution of systemic symptoms.

#### 3.1.6 Phenocopies of inborn errors of immunity

• ***Non-Clonal Hypereosinophilic Syndrome Due to Somatic Mutation in STAT5b GOF***

Somatic GOF mutations in *STAT5B*, particularly the N642H variant, were initially identified only in association with hematologic malignancies (156). Nevertheless, in 2016, Ma et al. described two patients with an identical somatic mutation in the *STAT5B* gene presenting with a non-neoplastic phenotype of early-onset, severe non-clonal hypereosinophilia, accompanied by urticaria, dermatitis, and diarrhea (157). Since then, this disorder has been included in the IUIS classification as a phenocopy of IEIs due to somatic variants (158). Lymphocytes from affected patients showed a significant increase in STAT5B phosphorylation in response to several cytokines, with only a minimal elevation at the basal state (157).

• ***Somatic JAK1 GOF (S703I)***

In 2020, Gruber et al. identified a *de novo* mutation in the *JAK1* gene (c.2108G >T, S703I) in a patient presenting with a complex autoinflammatory and atopic syndrome (159). From an early age, the patient suffered from an asymmetric pustular rash and chronic gastrointestinal tract inflammation. Later, he developed a membranous glomerulonephritis and multiple allergies. Elevated phosphorylation of STAT1, STAT3, STAT4, STAT5, and STAT6 was observed selectively, depending on the immune cell type. Treatment with tofacitinib reduced STAT hyperphosphorylation and improved disease outcomes.

### 3.2 JAK/STAT pathway in hematologic malignancies

**Figure 5:**
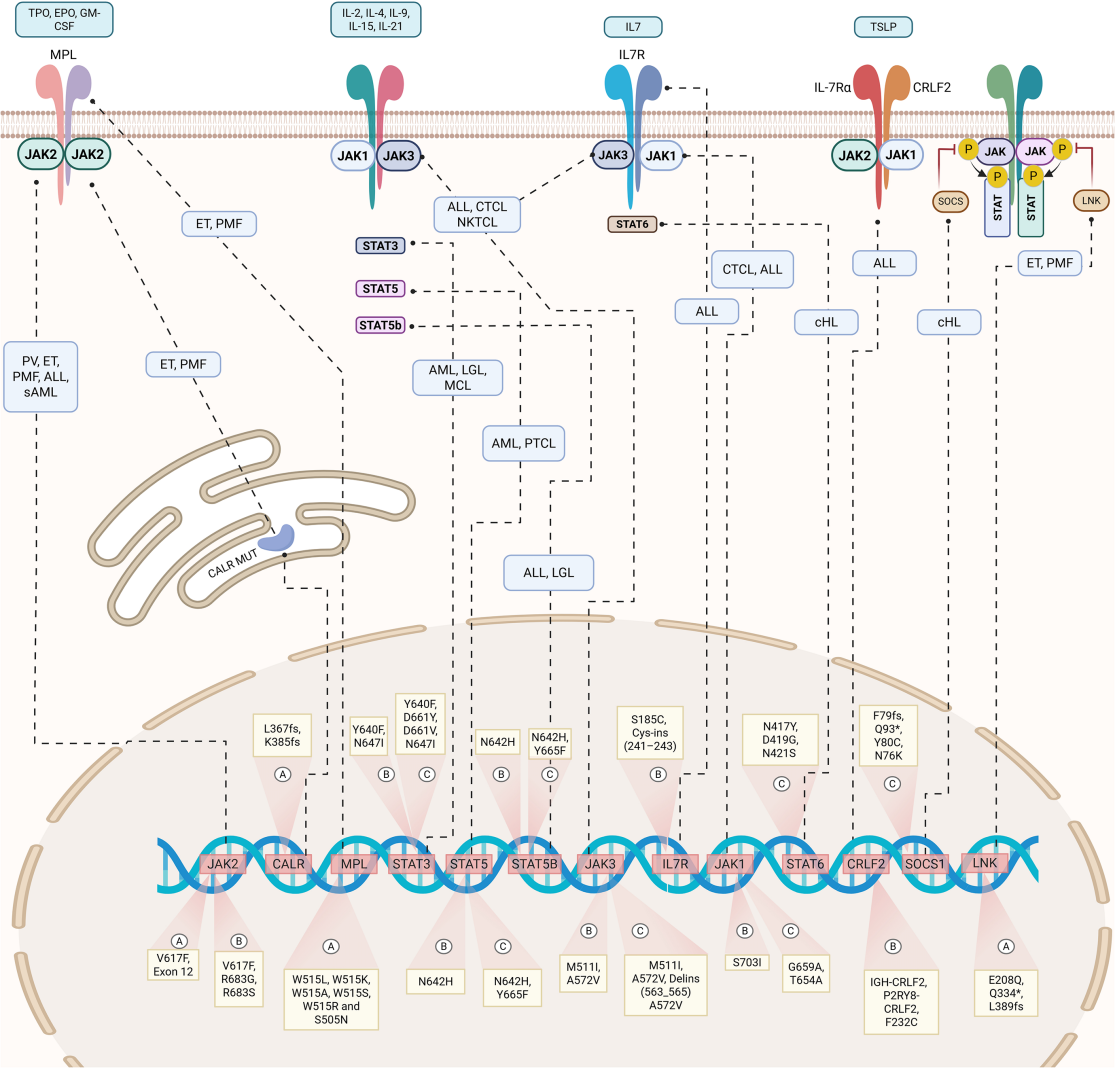
JAK-STAT pathway gene alterations in hematologic malignancies. Common pathogenic mutations are grouped by disease type: **(a)** myeloproliferative neoplasms, **(b)** acute leukemias, and **(c)** mature lymphoid neoplasms. Subtypes are indicated in blue boxes. ALL, Acute Lymphoblastic Leukemia; AML, Acute Myeloid Leukemia; cHL, Classical Hodgkin Lymphoma; CTCL, Cutaneous T-Cell Lymphoma; ET, Essential Thrombocythemia; LGLL, Large Granular Lymphocyte Leukemia; MCL, Mantle Cell Lymphoma; MLNs, Mature lymphoid neoplasms; NKTCL, Natural Killer/T-Cell Lymphoma; PMF, Primary Myelofibrosis; PTCL, Peripheral T-Cell Lymphoma; PV, Polycythemia Vera; sAML, Secondary Acute Myeloid Leukemia. Created in BioRender. djidjik, r (2025). https://BioRender.com/grqjm63.

Hematologic malignancies involve the abnormal proliferation and accumulation of blood cells and represent a heterogeneous group of diseases with complex molecular mechanisms. A key driver in the development and progression of these diseases is the dysregulation of the JAK-STAT signaling pathway. Aberrant activation of JAK-STAT signaling, often driven by genetic mutations (**supplementary tables**) that occur upstream or within core components of the pathway, plays a pivotal role in the pathogenesis of various hematologic malignancies, including myeloproliferative neoplasms, acute leukemias, and mature lymphoid neoplasms (160). These diseases will be reviewed in decreasing order of reported mutation frequency to enable a structured analysis of the most commonly affected conditions. (**Figure 5**).

#### 3.2.1 Myeloproliferative neoplasms

Myeloproliferative neoplasms (MPNs) are a group of clonal hematologic malignancies originating from hematopoietic stem cells. They are characterized by the excessive proliferation of one or more blood cell lineages, typically leukocytes, erythrocytes, or platelets, depending on the specific disease subtype. These disorders are associated with an elevated risk of thrombosis, progressive myelofibrosis, and leukemic transformation. Mutations affecting key components of the JAK-STAT pathway, including the driver mutations JAK2, Calreticulin (CALR), and the myeloproliferative leukemia virus oncogene (MPL), are commonly observed in MPN patients (161, 162).

• ***JAK2***

As discussed in the first section, the JAK2 protein, a member of the tyrosine kinase family, is involved in the signal transduction of several cytokine receptors, including erythropoietin (EPO), thrombopoietin (TPO), and granulocyte-colony stimulating factor (G-CSF).

The JAK2 V617F mutation is a hallmark driver mutation and the most frequent mutation in Philadelphia chromosome (Ph)-negative MPNs. It is found in over 90% of patients with polycythemia vera (PV), approximately 50%-60% of patients with essential thrombocythemia (ET), and primary myelofibrosis (PMF) (16, 163, 164). This mutation is one of the major diagnostic criteria for Ph-negative MPNs. It results from a guanine-to-thymine substitution at nucleotide 1849 in exon 14 of the JAK2 gene, leading to a valine-to-phenylalanine substitution at position 617 in the pseudokinase domain (JH2). The mutation disrupts the inhibitory function of this domain, resulting in the constitutive activation of the kinase’s catalytic (JH1) domain and persistent stimulation of downstream STAT transcription factors, notably STAT3, STAT5, and STAT6. This leads to cytokine hypersensitivity and cytokine-independent growth of myeloid cells, contributing to oncogenic processes in hematologic malignancies. Additionally, the mutation activates the PI3K-AKT and Ras-Raf-MAPK-ERK signaling pathways, making it a significant oncogenic driver (165).

Mutations within exon 12 of the JAK2 gene also contribute significantly to the pathogenesis of Ph-negative MPNs. Similar to JAK2 V617F, these mutations impair the autoinhibition of the JAK2 kinase, resulting in its constitutive activation. Most exon 12 mutations affect residues between residues 537 and 547 (substitutions, deletions, and deletions-insertions). Unlike JAK2 V617F, exon 12 mutations are almost exclusively found in patients with PV, accounting for approximately 2-5% of cases (166, 167).

• ***CALR***

Calreticulin is an endoplasmic reticulum chaperone protein involved in protein folding and is the second most commonly mutated protein in MPNs, following JAK2. Mutations in CALR Exon 9, which are a major diagnostic criterion for Ph-negative MPNs, are found in 67% to 88% of patients with ET or PMF who lack JAK2 mutations (18, 168). The most frequent mutations, L367fs*46 (Type I) and K385fs*47 (Type II), generate a novel C-terminal peptide that aberrantly activates the TPO receptor or MPL through homomultimerization. This interaction results in the constitutive activation of MPL-associated JAK2, driving cytokine-independent signaling (18). CALR mutations are mutually exclusive with other MPNs driver mutations, including JAK2 V617F and MPL mutations (18). Recent findings have demonstrated that mutant CALR induces cytokine-independent activation of MPL through a specific interaction mechanism. This involves the binding of mutant CALR to immature MPL via asparagine-linked glycans within the endoplasmic reticulum, facilitating the formation of a CALR-MPL complex. This complex is subsequently trafficked to the cell surface, where it promotes constitutive activation of the downstream kinase JAK2 associated with MPL (169). Patients with CALR-mutated PMF typically experience better outcomes, including higher platelet and hemoglobin levels, lower thrombosis risk, and longer survival (18).

• ***MPL***

The TPO receptor or MPL is a key component of the TPO-JAK2-STAT signaling pathway, playing a crucial role in the JAK2-STAT signaling cascade. Mutations in MPL can aberrantly activate downstream signaling pathways, contributing to tumorigenesis and disease progression. Common MPL mutations include W515L, W515K, W515A, W515S, W515R, and S505N (169). These mutations are present in 3% to 5% of patients with ET and 8% to 10% of patients with PMF, representing one of the diagnostic criteria for Ph-negative MPNs (170). MPL mutations in ET patients are typically associated with elevated platelet counts, lower hemoglobin levels, increased arterial thrombosis risk, and poorer overall survival. A cohort study of MPN patients and analysis of bone marrow-derived DNA from different disease stages have consistently shown that MPL W515L and JAK2 V617F co-occur in MPNs, suggesting that these mutations may complement each other in driving MPN pathogenesis (171).

• ***LNK***

LNK, a lymphocyte adaptor protein that negatively regulates the JAK-STAT signaling pathway, plays a crucial role in MPNs. Loss-of-function mutations in the LNK gene (SH2B3), mostly missense or frameshift mutations, lead to activation of the JAK-STAT pathway, promoting hematopoietic stem cell proliferation (172, 173).

#### 3.2.2 Acute lymphoblastic leukemias

Acute lymphoblastic leukemia (ALL) is an aggressive hematologic malignancy characterized by the malignant transformation of developing lymphoid precursor cells, driven by the accumulation of genetic alterations. The pathogenesis of ALL involves multiple signaling pathways, with recent studies highlighting the significant role of the JAK-STAT pathway. Dysregulated activation of this pathway promotes abnormal cell proliferation and inhibits apoptosis, contributing to the onset of ALL (19).

• ***JAK2***

Gain-of-function mutations in JAK2, frequently observed in ALL, are primarily located in exon 16, notably affecting arginine at position 683 (R683), which is most commonly substituted with glycine (R683G) or serine (R683S). A rare JAK2 ΔIREED mutation, involving a five–amino acid deletion within the JH2 pseudokinase domain, has also been reported (19, 174). In addition, JAK2 fusion genes resulting in constitutive kinase activity have been described, although rarely in ALL, including ETV6-JAK2 (175) and PCM1-JAK2 fusions (19, 176), all of which are associated with poor prognosis. Nonetheless, JAK2 mutations often require cooperating genetic alterations, particularly cytokine receptor-like factor 2 (CRLF2) rearrangements or additional mutations in JAK family members (177).

• ***CRLF2***

Thymic stromal lymphopoietin (TSLP) signals through a receptor composed of CRLF2 (on X/Y chromosomes) and IL-7Rα, which are constitutively associated with JAK2 and JAK1, respectively (178, 179). CRLF2 alterations, frequently co-occurring with JAK2 mutations, contribute to the constitutive activation of STAT5. These changes promote cytokine-independent proliferation and survival of early hematopoietic cells, thereby facilitating the development of B-cell precursor acute lymphoblastic leukemia (BCP-ALL) (178).

CRLF2 overexpression is primarily driven by chromosomal rearrangements involving IGH or P2RY8 (179).

• ***IL-7R***

A key upstream mutation associated with the abnormal activation of the JAK-STAT pathway in T-ALL is the dysregulated expression of the interleukin 7 receptor (IL-7R). Aberrant IL-7R expression drives oncogenic programs in over 70% of T-ALL cases. IL-7R, predominantly expressed in lymphoid cells, is crucial for T cell development and maintaining homeostasis in mature T cells. Activating mutations in IL7R, notably those causing homodimerization of IL-7Rα, such as the S185C substitution in the extracellular domain or insertions or deletions in the transmembrane domain of IL-7R, enhance JAK–STAT5 signaling and are found in approximately 12% of Ph-like ALL and 2–3% of B-ALL cases (180, 181).

• ***JAK1 and JAK3***

Sustained activation of the JAK-STAT signaling pathway is a key driver in the initiation and progression of T-cell acute lymphoblastic leukemia (T-ALL), with activating mutations in JAK1 (e.g., V658F) and JAK3 (e.g., M511I) commonly reported in T-ALL cases. Recent studies suggest that T-ALL arises through a multistep transformation process involving the accumulation of various genetic defects, such as activating mutations in the JAK-STAT pathway (182).

• ***PTPN2***

Enhanced activation of the JAK-STAT pathway in T-ALL can result from mutations and deletions in protein tyrosine phosphatase non-receptor type 2 (PTPN2). Loss of PTPN2 function increases IL-7R signaling responsiveness, thereby amplifying downstream JAK-STAT activation (17).

• ***STAT5***

The STAT5B N642H hotspot mutation has been commonly found in pediatric T-cell acute lymphoblastic leukemia (T-ALL) and is associated with poor prognosis and an increased risk of relapse (183). In addition, the less frequent STAT5B Y665F mutation, as well as other rare missense mutations in STAT5A or STAT5B affecting the SH2 or transactivation domains, have been reported (184). These gain-of-function mutations result in constitutive activation of the JAK-STAT pathway, promoting leukemic cell proliferation and survival.

#### 3.2.3 Mature lymphoid neoplasms

Studies have shown that aberrant activation and mutations of the JAK-STAT signaling pathway are frequently detected in various mature B-cell, T-cell, natural killer (NK) cell neoplasms, and Hodgkin lymphomas (185).

• ***STAT3, STAT5, STAT6, and SOCS1***

Aberrations in the JAK-STAT pathway are critical features of classical Hodgkin lymphoma (cHL), with frequent mutations in STAT6 (32%) and SOCS1 (59%). In cHL, Pleiotrophin activates JAK2/STAT6, contributing to immune evasion and tumor plasticity. Elevated cytokines, such as IL-21, further stimulate STAT5, enhancing tumor growth (186).

In mantle cell lymphoma (MCL), STAT3 mutations, although rare, can lead to constitutive activation of the JAK/STAT pathway, promoting tumor cell survival, proliferation, and immune evasion (186).

Elevated expression of STAT5A/B has also been detected in human PTCL samples. Both factors have been confirmed as oncogenes in PTCL, with STAT5B being more transforming (187).

Moreover, mutations in STAT3 and STAT5B genes have been detected in patients with large granular lymphocytic leukemia (LGLL), with the STAT5B N642H mutation linked to unfavorable disease progression (156, 188).

• ***JAK1 and JAK3***

Recent studies have identified JAK1 as an oncogenic driver in various lymphomas, particularly in relapsed or refractory peripheral T-cell lymphoma (PTCL), where its sustained activation is associated with enhanced tumor proliferation, survival, migration, and suppression of anti-tumor immunity (189).

Deregulated JAK/STAT signaling due to JAK1 and JAK3 somatic mutations has also been observed in Cutaneous T-Cell Lymphoma (CTCL), supporting malignant proliferation (190).

In natural killer T-cell lymphoma (NKTCL), JAK3 mutations (A572V/A573V) occur in approximately 35% of cases and drive constitutive JAK3/STAT5 activation, promoting proliferation (190).

#### 3.2.4 Acute myeloid leukemia

Acute myeloid leukemia (AML) is a genetically heterogeneous malignancy characterized by the clonal expansion of hematopoietic stem and progenitor cells (HSPCs), leading to the accumulation of immature myeloid precursors in the bone marrow and peripheral blood. This proliferation disrupts normal hematopoiesis, resulting in cytopenia such as anemia, leukopenia, and thrombocytopenia (19).

The development of AML is considered a multistep and multifactorial process (191). Mutations within the JAK/STAT signaling pathway have been implicated not only in secondary acute leukemias (sAML) arising from MPNs but also in *de novo* acute leukemias (192, 193). This aberrant signaling is associated with poor prognosis and serves as a predictive biomarker for disease progression.

• ***JAK2***

High expression levels of JAK2 V617F promote the transformation from MPN to sAML by enabling cytokine-independent survival and proliferation of myeloid progenitor cells (194, 195). JAK2 exon 12 mutations can also occur in sAML derived from PV (167).

Fusion genes such as PCM1–JAK2 (174, 177), BCR–JAK2 (174, 196), and ETV6–JAK2 have been identified in *de novo* AML, leading to constitutive kinase activation (175). This results in persistent activation of downstream signaling, which can lead to unregulated cell division and evasion of apoptotic signals, key hallmarks of AML.

• ***STAT 3 and STAT 5***

Although direct STAT3 and STAT5 mutations are rare in AML, constitutive activation of these transcription factors is frequently observed, with studies reporting activation in 44–76% of patients. This persistent activation is often driven by upstream mutations or cytokine signaling and is associated with chemoresistance and poor prognosis (197, 198).

#### 3.2.5 Indirect genetic activation of JAK-STAT in hematologic malignancies

In addition to canonical cytokine stimulation, various genetic alterations modulate the JAK-STAT pathway in hematologic malignancies, reinforcing disease progression. Lysine (K)-methyltransferase 2A *(*KMT2A) rearrangements, common in ALL and AML, enhance JAK-STAT signaling by downregulating IL-7R/IL-4R and suppressing KMT2A expression (199). Similarly, the BCR-ABL1 fusion, particularly the p190 isoform in Ph-positive B-ALL, directly activates STAT5, either independently or via JAK2, driving proliferation and correlating with poor prognosis (200). In lymphoplasmacytic lymphoma, the MYD88 L265P mutation leads to constitutive STAT3 activation, promoting B-cell survival and immune evasion (201). In Hodgkin lymphoma, PD-L1/PD-L2: 9p24.1 alterations and PD-L1/PD-L2 mutations drive JAK-dependent overexpression of PD-L1/PD-L2, facilitating immune escape via PD-1 signaling (4). In AML, mutations in kinases such as FMS-like tyrosine kinase 3 (FLT3*)* can activate STAT5 independently of JAK2, particularly in the presence of co-occurring nucleophosmin *(*NPM1*)* or DNA methyltransferase 3A (DNMT3A*)* mutations, which further upregulate MYC and BCL2 (202–204). Additionally, gain-of-function KIT mutations (e.g., D816V), along with alterations in CCAAT/enhancer-binding protein alpha (CEBPA*)* and colony-stimulating factor 3 receptor (CSF3R*)*, increase JAK-STAT pathway sensitivity in AML (205). The JAK-STAT pathway also interacts with p53 signaling in hematologic malignancies: JAK2 V617F promotes p53 degradation via murine double minute 2 (MDM2*)* and La protein through the PI3K/Akt/mTOR pathway, while STAT1 counters this by stabilizing p53. Conversely, STAT3 represses TP53 transcription, and STAT5 disrupts NPM1-mediated p53 stabilization. Additionally, the protein inhibitor of activated STAT Y (PIASy*)*, a STAT inhibitor, further suppresses p53 activity (206).

Collectively, these findings underscore the central role of the JAK-STAT pathway in the pathogenesis of various hematologic malignancies, including MPNs, acute leukemias, and mature lymphoid neoplasms. Aberrant activation of this pathway, driven by somatic mutations, chromosomal rearrangements, or dysregulated cytokine signaling, promotes malignant cell proliferation, survival, and immune evasion. Recurrent alterations in core components such as JAK1, JAK2, JAK3, STAT3, STAT5, and STAT6 not only contribute to oncogenesis but also represent actionable therapeutic targets. The development of JAK inhibitors, particularly in combination with immune checkpoint blockade, has opened promising therapeutic avenues. Ongoing elucidation of the molecular mechanisms governing JAK-STAT dysregulation will be critical to advancing precision medicine approaches in hematologic cancers.

## 4 Laboratory-based investigation of the JAK/STAT pathway

### 4.1 Initial assessment of JAK/STAT pathway activity

#### 4.1.1 Analysis of JAK-STAT pathway phosphorylation

JAK-STAT phosphorylation can be studied using various approaches, each providing specific types of information. The most common method is Western blotting following immunoprecipitation from activated cell extracts using anti-phosphotyrosine (a-pY) antibodies. Cells are first lysed in an appropriate buffer, and the targeted JAK or STAT protein is immunoprecipitated, separated by SDS-PAGE, transferred to a membrane, and detected by Western blot. The identity of the phosphorylated protein is then confirmed using JAK- or STAT-specific antibodies. However, Western blot analyses performed on heterogeneous cell populations yield results that are difficult to interpret, making it necessary to use pre-sorted cells. In contrast, a flow cytometry-based method for detecting phosphorylated proteins provides clearer results, and cell populations can be identified simultaneously. After cytokine stimulation, the cells are fixed with paraformaldehyde and then permeabilized using a cold methanol/acetone mixture. The cells are then washed and stained with a phospho-specific antibody that is either directly conjugated to a fluorochrome or followed by a fluorescent secondary antibody if the primary antibody is unlabeled. Surface staining must also be performed to target the cellular subpopulations of interest. After several washes, the cells are analyzed by flow cytometry (207).

#### 4.1.2 Analysis of STAT-induced transcriptional activity

While tyrosine phosphorylation of STAT proteins is crucial for their activation, the primary biological outcome of JAK-STAT pathway stimulation is the altered expression of its target genes (208, 209).

The transcriptional activity of STAT proteins can be assessed by measuring the expression levels of well-characterized target genes such as GBP1, SOCS1, SOCS2, SOCS3, ABCB2, CCND1, c-Myc, Bcl-xL, and Cyclin D1 (210). Total RNA is extracted from cells using a suitable reagent, such as TRIzol or an equivalent kit, followed by DNase I treatment to eliminate any genomic DNA contamination. RNA concentration and purity are evaluated by spectrophotometry, and RNA integrity is assessed by agarose gel electrophoresis or using a bioanalyzer. For quantitative PCR (qPCR), total RNA is first reverse transcribed using random hexamer primers and a reverse transcriptase. qPCR is then carried out using gene-specific primers and a SYBR Green or TaqMan detection system on a real-time thermocycler (211). Gene expression levels are normalized to reference genes such as GAPDH or ACTB.

As a complement or alternative, high-throughput RNA sequencing (RNA-seq) can be used to globally analyze the transcriptome. RNA libraries are prepared from enriched mRNA (by polyA tail selection) or after rRNA depletion, followed by fragmentation, cDNA synthesis, and adaptor ligation. Sequencing is performed using single-end or paired-end reads. Raw reads are then aligned to the reference genome, and transcript abundance is quantified. Differential gene expression analysis is conducted using bioinformatics tools such as DESeq2 or EdgeR. This approach enables accurate and comprehensive evaluation of STAT-dependent transcriptional regulation in response to specific stimuli or conditions (212).

#### 4.1.3 Measurement of cytokines

Quantification of cytokines is essential for evaluating the activation status of the JAK/STAT signaling pathway. Enzyme-linked immunosorbent assays (ELISA) remain the gold standard for the quantification of individual cytokines, offering high sensitivity and specificity. However, ELISA is generally limited to the detection of one analyte per sample. In contrast, multiplex assays such as Luminex xMAP technology or Meso Scale Discovery (MSD) electrochemiluminescence platforms enable the simultaneous detection and quantification of multiple cytokines in small sample volumes. These bead- or spot-based technologies employ fluorescent or electro-chemiluminescent tags bound to capture antibodies specific to the target cytokines, enabling parallel profiling of complex cytokine networks (213–218). Multiplexing is especially valuable when analyzing cytokine panels in inflammatory or immune signaling contexts, where multiple STAT activators may be present. Sample preparation typically involves collection of conditioned media from cultured cells or plasma/serum from *in vivo* studies, followed by centrifugation and dilution according to assay requirements. Controls and standards must be included for accurate quantification, and results are usually reported in pg/mL or ng/mL. Assay sensitivity, cross-reactivity, and dynamic range should be carefully considered during assay selection and data interpretation.

Quantitative cytokine measurements serve not only to confirm the presence of signaling molecules but also to correlate cytokine abundance with downstream STAT phosphorylation and transcriptional output, thereby providing a comprehensive picture of pathway activation.

### 4.2 Genetic and epigenetic analysis of the JAK/STAT pathway

Genetic mutations and epigenetic alterations are key drivers of JAK/STAT pathway dysregulation in human disease. Integrating mutation analysis, CNV profiling, and epigenetic mapping provides critical insights for disease classification, prognosis, and the tailored use of targeted therapies such as JAK inhibitors.

#### 4.2.1 Mutation analysis

• ***Quantitative PCR (qPCR) Diagnosis: Targeted and Rapid Detection***

qPCR is widely used for detecting known point mutations in key genes of the JAK/STAT pathway, such as *JAK2*, *STAT3*, and *STAT5B*. This method uses specific probes or allele-discriminating primers capable of distinguishing wild-type from mutant alleles. A classic example is the JAK2 V617F mutation, found in over 90% of patients with polycythemia vera and in a significant proportion of those with essential thrombocythemia or primary myelofibrosis (219–222). Commercial qPCR kits offer rapid and sensitive detection of these mutations. However, qPCR is limited to the detection of predefined mutations and is therefore not suitable for identifying rare or novel mutations, nor for comprehensively analyzing entire gene regions. Nevertheless, it remains a preferred technique for targeted screening in clinical diagnostics or for monitoring mutation burden over time (223, 224).

• ***Sanger Sequencing: Validation and Point Mutation Detection***

Sanger sequencing remains the gold standard for identifying point mutations, small insertions, or deletions in JAK/STAT pathway genes. It enables direct reading of nucleotide sequences, typically focusing on the coding exons of genes (164, 225, 226). This technique is often used to validate results from other approaches (qPCR or NGS), or in translational research settings. While highly accurate, Sanger sequencing has certain limitations: it lacks sensitivity for detecting low-frequency mutations (allelic frequency <15–20%) and is less efficient for large gene panels. Thus, it is less suitable for complex diagnostic needs requiring broader coverage or high sensitivity (227).

• ***Next-Generation Sequencing (NGS): Comprehensive Analysis of the JAK/STAT Pathway***

NGS has revolutionized molecular diagnostics by enabling massively parallel analysis of numerous genes, entire exomes, or even whole genomes. Three main strategies are commonly used:

- **Targeted Gene Panels (TGP)**: TGPs are NGS-based assays designed to selectively analyze a predefined set of genes associated with specific diseases or biological pathways. They focus on capturing and sequencing only the exons or relevant regions of selected genes, enabling high coverage and sensitivity for detecting pathogenic variants, including single nucleotide variants (SNVs), insertions/deletions (indels), and copy number variations (CNVs) (228, 229). TGPs targeted hematological diseases, IEIs, or signaling pathways often include core JAK/STAT pathway genes. These panels can detect somatic or germline mutations, as well as GOF or LOF mutations (62, 230–232). In oncology, TGPs are also used to identify actionable mutations in the JAK/STAT axis, helping to inform therapeutic decisions and guide personalized medicine strategies (e.g., JAK inhibitor sensitivity) (233–236).
- **Whole Exome Sequencing (WES):** WES focuses on analyzing the protein-coding regions of the genome, which represent approximately 1–2% of the entire genome but harbor around 85% of disease-causing mutations (237). By targeting exons, WES enables the identification of both known and novel mutations that can lead to various monogenic diseases. It is particularly valuable in atypical clinical presentations or when routine targeted sequencing fails to detect pathogenic variants. In the context of the JAK/STAT pathway, WES allows for the discovery of rare or *de novo* mutations in coding regions of genes such as JAK1-3, TYK2, and STAT1-6, which may be implicated in immunodeficiencies, autoimmune syndromes, or hematological disorders. Furthermore, WES can detect mutations in regulatory genes or genetic modifiers that influence the function of the JAK/STAT pathway, often overlooked by standard gene panels limited to core pathway genes (238–240).
- **Whole Genome Sequencing (WGS):** WGS provides comprehensive coverage of the entire genome, including coding, non-coding, regulatory, intronic, and intergenic regions. This broader scope enables the detection of deep intronic mutations, enhancer or promoter alterations, structural variants (e.g., insertions, deletions, inversions), and CNVs that may dysregulate gene expression or disrupt chromosomal architecture (241–244). Such genomic events can influence the expression and regulation of JAK/STAT components or their upstream/downstream effectors, contributing to complex or cryptic disease phenotypes. Although WGS is more expensive and computationally demanding than WES, it is increasingly used in both research and clinical settings for patients with undiagnosed genetic conditions or for those with inconclusive prior testing (245). In particular, WGS can uncover regulatory elements or structural anomalies that modulate the JAK/STAT pathway and are critical for comprehensive diagnosis and precision medicine approaches.

#### 4.2.2 Copy number variations

In cancers, CNVs, including deletions or amplifications, frequently lead to JAK-STAT pathway dysregulation. For example, amplification of STAT3 or STAT5B has been reported in T-cell large granular lymphocytic leukemia and other malignancies (156). Detection of CNVs can be achieved via fluorescence *in situ* hybridization (FISH), qPCR, or more comprehensively through NGS-based copy number profiling and comparative genomic hybridization (CGH) arrays (246). These methods provide insight into dosage alterations that may influence pathway output and therapeutic resistance.

#### 4.2.3 Epigenetic modifications

The epigenetic landscape also contributes to the regulation of JAK/STAT signaling. Promoter hypermethylation of STAT5A, STAT5B, or SOCS1/3 genes has been implicated in the silencing of pathway modulators in various cancers and immune disorders (247, 248). DNA methylation patterns are typically assessed by bisulfite sequencing, methylation-specific PCR, or methylation arrays. Additionally, histone modifications, such as H3K27ac and H3K4me3 at cytokine-responsive promoters, can be evaluated using chromatin immunoprecipitation followed by sequencing (ChIP-seq). These epigenetic signatures provide insights into the transcriptional regulation of STAT target genes and are often integrated with transcriptomic analyses (e.g., RNA-seq) to elucidate downstream effects (249).

### 4.3 Functional assays

#### 4.3.1 Luciferase reporter assays

Luciferase reporter assays are widely used to monitor the transcriptional activity of STAT proteins in living cells. In this approach, cells are transfected with plasmids containing STAT-responsive elements upstream of a luciferase reporter gene (250). Commonly used constructs include pSTAT3-TA-Luc and GAS-Luc, which incorporate tandem repeats of gamma-activated sequences (GAS) that are specifically recognized by activated STATs such as STAT1 or STAT3. Upon cytokine stimulation (e.g., IL-6, IFN-γ), activated STATs translocate to the nucleus and bind to these elements, promoting luciferase expression. The resulting luminescent signal, measured with a luminometer, reflects the degree of STAT-dependent transcriptional activation. This method provides a sensitive, quantitative, and rapid readout of STAT signaling dynamics in response to genetic manipulation or pharmacological inhibition (251, 252).

#### 4.3.2 siRNA/shRNA knockdown of JAK/STAT components to see effects on signaling

To investigate the functional role of JAK/STAT pathway components in signal transduction, gene silencing is performed using small interfering RNAs (siRNAs) or short hairpin RNAs (shRNAs) targeting JAK1, JAK2, STAT1, STAT3, or STAT5.

In brief, siRNA duplexes are transfected into cultured cells using Lipofectamine™ RNAiMAX (Thermos Fisher Scientific) a lipid-based transfection reagent optimized for high-efficiency siRNA delivery with minimal cytotoxicity. Knockdown efficiency is evaluated by quantitative reverse transcription PCR (qRT-PCR) or immunoblotting 2–3 days post-transfection. Non-targeting and positive control siRNAs are included respectively as a negative control and to monitor transfection efficiency. This approach enables efficient and reproducible silencing of target genes involved in critical cellular signaling pathways. For example, it has been employed to dissect the roles of individual components of the JAK/STAT pathway, such as STAT1, STAT3, JAK1, and JAK2, in cytokine signaling and immune regulation (253).

For shRNA-mediated knockdown, lentiviral vectors expressing shRNA sequences under the control of the U6 promoter, were generated by co-transfection into HEK293T cells along with plasmids, as described by Campeau et al. (254). Viral supernatants are harvested and used to infect target cells in the presence of polybrene followed by puromycin selection for 48–72 hours to enrich for transduced populations. Knockdown efficiency can be assessed by RT-qPCR and confirmed at the protein level via Western blot using antibodies against total and phosphorylated forms of the targeted JAK and STAT proteins. To evaluate the functional consequences of the knockdown, cells are stimulated with cytokines known to activate the pathway, followed by assessment of STAT phosphorylation using phospho-specific antibodies and quantification of downstream target gene expression (*SOCS1*, *SOCS3*, *Bcl-xL*, *c-Myc*) by qPCR (255, 256). This strategy enables a mechanistic dissection of individual JAK/STAT components and their specific contributions to signal transduction and transcriptional regulation.

#### 4.3.3 Overexpression of constitutively active STAT mutants

Overexpression of constitutively active (CA) STAT mutants is a widely used strategy to study GOF effects of STAT signaling in various cellular contexts (257). These CA mutants are designed to mimic the active, phosphorylated state of STAT proteins, thereby bypassing the need for upstream cytokine or growth factor stimulation. This approach allows researchers to dissect the direct transcriptional and phenotypic consequences of sustained STAT activation under controlled experimental conditions.

A well-known example is the STAT3-C mutant, in which two cysteine residues are introduced into the SH2 domain (at positions A661C and N663C). This modification promotes the formation of stable disulfide-linked dimers, enabling cytokine-independent nuclear localization and DNA binding (257). Similarly, other mutations such as Y640F in STAT1 or D661V in STAT3, which occur in the SH2 domain or in regions that regulate dimerization, have been reported to enhance STAT activity and are sometimes found in hematologic malignancies and immune disorders (258, 259).

The functional impact of CA-STATs can be assessed by introducing these mutants into cells via plasmid transfection, viral transduction, or genome editing. Downstream effects are typically evaluated using luciferase reporter assays to measure transcriptional activity, or qPCR/RNA-seq to quantify the expression of well-established STAT target genes such as *SOCS1*, *SOCS3*, *Bcl-xL*, *Cyclin D1*, and *c-Myc* (260). Moreover, CA-STATs often induce biological changes such as enhanced cell proliferation, increased survival, or even cellular transformation, reflecting the oncogenic potential of deregulated STAT signaling (261).

#### 4.3.4 CRISPR-Cas9 knockout

CRISPR-Cas9 Knockout technology offers a precise and efficient method for disrupting specific genes to investigate their functional roles in signaling pathways such as JAK/STAT. By designing guide RNAs targeting exons of genes encoding pathway components (e.g., JAK1, JAK2, STAT1, STAT3), researchers can introduce targeted double-strand breaks, which are repaired through non-homologous end joining (NHEJ), often resulting in frameshift mutations and gene knockout (262, 263).

Establishing STAT or JAK knockout (KO) cell lines using CRISPR-Cas9 allows for a direct evaluation of the dependence of downstream responses, such as cytokine-induced gene expression, transcriptional activity, and phenotypic outcomes, on specific pathway components. These KO models are especially useful for distinguishing between canonical and non-canonical STAT functions and for confirming the specificity of small-molecule inhibitors or siRNA effects (264–267). For example, Zhou et al. used CRISPR-mediated STAT3 knockout in A549 cells to show the essential role of STAT3 in IL-6-induced gene expression and tumorigenesis (268). Similarly, Zhang et al. demonstrated that knocking out JAK1 or JAK2 abrogated interferon signaling, validating pathway specificity (269).

#### 4.3.5 Receptor blocking strategies to inhibit JAK/STAT activation

To inhibit cytokine-induced activation of the JAK/STAT pathway, receptor blocking approaches can be employed. This involves using neutralizing antibodies against cytokine receptors or applying small-molecule inhibitors targeting associated kinases. For instance, anti-IL-6R antibodies (such as Tocilizumab) effectively block IL-6 binding to its receptor, thereby preventing downstream STAT3 phosphorylation and activation (270). Similarly, JAK inhibitors such as Ruxolitinib, a selective JAK1/2 inhibitor, suppress cytokine signaling by preventing the phosphorylation of STAT proteins downstream of receptor engagement (271). These interventions allow for the functional analysis of cytokine pathways and are instrumental in both mechanistic studies and therapeutic applications.

### 4.4 Diagnostic Approaches in Diseases Involving the JAK/STAT Pathway

As mentioned before, aberrations in the JAK/STAT signaling pathway have been implicated in a wide spectrum of human diseases, including malignancies, autoimmune disorders, and primary immunodeficiencies. Laboratory investigations of these conditions involve the integration of the different tests mentioned above to accurately assess pathway dysregulation (**Supplementary Figure**).

A prime example can be found in hematological malignancies, such as ALL, lymphoma, and MPNs, genetic alterations affecting JAK2 are frequently observed. Among these, fusion events such as TEL-JAK2 (ETV6-JAK2) and PCM1-JAK2 result in constitutive activation of the JAK2 kinase, leading to persistent STAT phosphorylation and oncogenic transcriptional programs (272, 273). Detection of such fusions can be performed by reverse transcription PCR (RT-PCR) targeting the fusion junctions or by fluorescence *in situ* hybridization (FISH) to detect the underlying chromosomal translocations. Additionally, the JAK2 V617F point mutation, a hallmark of classical MPNs, is routinely screened using allele-specific PCR, high-resolution melting (HRM) analysis, or Sanger sequencing (274). Downstream activation of STATs, particularly STAT5, can be assessed using phospho-flow cytometry or Western blotting, providing functional evidence of pathway activation.

In autoimmune disorders such as rheumatoid arthritis, systemic lupus erythematosus, and psoriasis, aberrant activation of the JAK/STAT pathway, especially involving STAT3 and STAT5, is commonly observed. This hyperactivation is primarily driven by elevated levels of cytokines such as IL-6, IL-21, and IL-23, which enhance Th17 differentiation and promote chronic inflammation. Laboratory evaluation typically involves stimulation of peripheral blood mononuclear cells (PBMCs) with relevant cytokines, followed by assessment of STAT phosphorylation using specific antibodies in flow cytometry or immunoblotting formats (264). Transcriptional activation of downstream targets (e.g., *SOCS3*, *IL17A*, *BCL-XL*) is measured using quantitative PCR (qPCR). Concurrently, serum or plasma levels of inflammatory cytokines can be quantified using ELISA or multiplex bead-based assays, providing a broader picture of the inflammatory milieu.

For IEIs, as previously discussed, several monogenic disorders involving mutations in JAKs or STATs lead to inborn errors of immunity. LOF mutations in STAT3 underlie AD-HIES, characterized by eczema, recurrent infections, and impaired Th17 differentiation. Diagnosis involves STAT3 gene sequencing, typically by Sanger or whole exome sequencing (WES), and functional assays such as IL-6-induced STAT3 phosphorylation (by phospho-flow cytometry or Western blot) and Th17 differentiation assays with IL-17A measurement by ELISA or intracellular cytokine staining (275, 276). Conversely, GOF mutations in STAT3 lead to early-onset autoimmunity and lymphoproliferation. Affected individuals show elevated basal STAT3 phosphorylation and increased transcription of proinflammatory target genes, which can be assessed similarly via flow cytometry and qPCR. STAT1 GOF mutations, associated with chronic mucocutaneous candidiasis and autoimmunity, are diagnosed via STAT1 gene sequencing and functional demonstration of exaggerated STAT1 phosphorylation in response to IFN-α or IFN-γ. In contrast, STAT1 LOF mutations often associated with MSMD result in impaired interferon signaling and are confirmed by reduced STAT1 phosphorylation and target gene expression following stimulation (122). JAK3 deficiency, a well-established AR-SCID cause, results in defective signaling through the common γ-chain (γc) of several interleukin receptors. Immunophenotyping typically reveals a T−B+NK− phenotype, while JAK3 mutations are detected by sequencing (63, 277). Functional assays show absent phosphorylation of STAT5 upon stimulation with γc-dependent cytokines (e.g., IL-2, IL-7). Similarly, STAT5b deficiency impairs growth hormone signaling and immune regulation, with diagnosis relying on genetic testing and evaluation of STAT5 phosphorylation in response to IL-2 (278–280). STAT6 GOF are identified through sequencing and functional assays showing enhanced IL-4 induced STAT6 phosphorylation and elevated IgE production (87–89).

Taken together, these diverse disease entities underscore the critical role of precise molecular and functional assays in characterizing JAK/STAT pathway dysfunction. Integrating genomic sequencing, phospho-protein analysis, cytokine profiling, and cellular immune function assays provides a comprehensive framework for diagnosing and stratifying patients with JAK/STAT-related pathologies.

## 5 Advanced strategies for targeting the JAK/STAT pathway

The concept of inhibiting cytokines by targeting their signaling pathways with small molecules was introduced in 1995. The success of this approach stemmed from the discovery that these molecules could be specifically designed to inhibit protein kinases by targeting the ATP binding site. Several classes of therapeutics have been developed, with the majority focusing on inhibiting JAKs (Jakinibs), alongside growing interest in downstream STAT proteins and regulatory components. Some therapies have been approved, while many others remain in clinical trials (4).

JAK inhibitors (JAKi) represent an emerging class of small molecules that selectively target one or more members of the JAK family signaling pathway. This selectivity prevents STAT phosphorylation, activation, and subsequent gene transcription. Several JAK inhibitors have been developed and approved for clinical use in various IEI, immune-mediated inflammatory diseases and hematologic disorders (**Table 3**). While the first-generation inhibitors, which act as non-selective inhibitors, have demonstrated significant efficacy, they are associated with adverse events such as an increased risk of viral infections and hematological abnormalities. The development of second-generation molecules with selective inhibitory activity against JAKs aims to enhance safety profiles while preserving therapeutic benefits.

**Table 3:** Therapeutic Strategies Targeting the JAK-STAT Pathway.

JAK inhibitor	Target(s)	Main indications	Adverse effects
Tofacitinib	JAK1, JAK3 (partial JAK2, TYK2)	Rheumatoid arthritis, Psoriatic arthritis, Ulcerative colitis, Juvenile idiopathic arthritis	Infections (CMV, EBV, TB), anemia, leukopenia, hyperlipidemia, headache
Ruxolitinib	JAK1, JAK2	Myelofibrosis, Polycythemia vera, Graft-versus-host disease, Atopic dermatitis, STAT1/STAT3 GOF IEIs	Anemia, thrombocytopenia, neutropenia, viral infections
Baricitinib	JAK1, JAK2	Rheumatoid arthritis, Atopic dermatitis, Psoriatic arthritis, Vitiligo, COVID-19 (emergency use)	URTI, UTI, herpes zoster, anemia, neutropenia, lymphopenia, acne, headache
Upadacitinib	JAK1	Rheumatoid arthritis, Psoriatic arthritis, Atopic dermatitis, Ulcerative colitis	Infections, gastrointestinal symptoms, liver enzyme elevations
Filgotinib	JAK1	Rheumatoid arthritis, Crohn’s disease, Ulcerative colitis, Psoriatic arthritis	Infections, headache, nausea
Fedratinib	JAK2, BRD4, FLT3	Intermediate/high-risk myelofibrosis	Anemia, gastrointestinal toxicity, hepatotoxicity
Deucravacitinib	TYK2 (allosteric site)	Moderate-to-severe psoriasis	Nasopharyngitis, upper respiratory tract infections, elevated liver enzymes

The molecular mechanism of JAK inhibitors involves their binding mode and the type of interactions with amino acids in JAKs proteins, categorized as reversible (competitive) or irreversible (covalent) (4).

### 5.1 First generation janus kinase inhibitors

The first approved JAKi were tofacitinib and baricitinib for the oral treatment of rheumatoid arthritis and other autoimmune disorders. Numerous JAKi are currently under preclinical and clinical investigation. The first generation JAKi (tofacitinib, baricitinib, ruxolitinib) are ATP-competitive molecules, leading to the inhibition of multiple JAK family members and, consequently, the restricted release of various cytokines and growth factor (281).

• ***Ruxolitinib***

Ruxolitinib is a selective JAK1/2 inhibitor that blocks the ATP-binding catalytic site of these kinases, inhibiting their activation and thus the disruption of molecules and growth factors signaling pathways, reducing pro inflammatory cytokines and chemokine production.

Ruxolitinib received FDA approval for myelofibrosis in 2011, becoming the first JAK inhibitor. It was later approved for polycythemia vera in 2014 and for graft-versus-host disease in 2019. Ten years after its approval, ruxolitinib has demonstrated sustained efficacy and safety in treating polycythemia vera, especially in patients who are resistant or intolerant to hydroxyurea. Additionally, in 2021, the FDA approved ruxolitinib cream as the first topical JAKi for treating mild to moderate atopic dermatitis, which is generally considered safe even for young children. Ruxolitinib is also being explored for new therapeutic uses, particularly in alopecia areata, psoriasis, peripheral T-cell lymphoma, and relapsed diffuse large T-cell lymphoma. For the JAK/STAT gain-of-function IEI, the JAKi are increasingly recognized as promising therapeutic options. Since the first reported success of ruxolitinib in a STAT1-GOF patient in 2015, its efficacy has been documented in over 30 STAT1-GOF cases, more than 50 STAT3-GOF cases, and isolated cases of other JAK/STAT IEIs (SOCS1 haploinsufficiency, and GOF mutations in JAK1, STAT4, STAT5B, STAT6, as well as STAT1 loss of function) (282, 283).

Ruxolitinib dosing varies depending on the disease: for polycythemia vera and acute graft-versus-host disease, the starting dose is 10 mg twice daily; for myelofibrosis, it is 5–20 mg twice daily; and for acute and chronic graft-versus-host disease, it is 10 mg twice daily. Doses are adjusted during treatment based on patient tolerance and safety. The main side effects reported are hematological events, including anemia and thrombocytopenia, linked to JAK2 inhibition, which plays an important role in erythropoietin and thrombopoietin signaling. Other serious effects may include neutropenia and viral infections (284).

• ***Tofacitinib***

Tofacitinib was the first JAK inhibitor studied and approved by the FDA for rheumatoid arthritis, demonstrating efficacy in diverse patient groups, including those unresponsive to conventional DMARDs and methotrexate-naïve patients. This therapy has also been approved for several auto inflammatory and autoimmune disorders, such as psoriatic arthritis, ulcerative colitis, and polyarticular course juvenile idiopathic arthritis (285).

Pharmacological studies showed that JAK3 and JAK1 were primarily inhibited, with weaker effects on JAK2 and TYK2, further diminishing their inflammatory effects through the greatest effect on IL-6, IFNγ, and common γc cytokines.

Tofacitinib is available as 5 mg and 10 mg dosages, as well as an 11 mg extended-release tablet for adults, while a1 mg/mL oral solution is available for children aged 2 years and older. Adverse effects include a small percentage of patients developing nasopharyngitis, headache, and increased blood creatine phosphokinase and cholesterol levels. Severe adverse effects may include viral and bacterial infections such as cytomegalovirus, Epstein Barr Virus, BK virus, tuberculosis, malignancy, lymphoproliferative disorder, and hematological abnormalities (anemia and leukopenia) (286).

• ***Baricitinib***

This medication is FDA-approved for treating adults with moderate to severe rheumatoid arthritis who have not responded well to other DMARDs, including TNF inhibitor therapies. Additionally, clinical trials have reported that baricitinib effectively improves symptoms of severe atopic dermatitis, psoriatic arthritis, and vitiligo. The FDA granted emergency use authorization for the combination of baricitinib and remdesivir to treat severe hospitalized Covid-19 patients (287).

Baricitinib is an orally active small-molecule inhibitor of JAK1/2, preventing the phosphorylation and activation of STATs. This molecule can modulate the signaling pathways of various interleukins and growth factors (IFNγ, IL-6, IL12/23, EPO, and GM-CSF) and induces cell apoptosis. In patients with rheumatoid arthritis, baricitinib treatment significantly induced an accelerated decrease of serum inflammatory proteins.

The therapy is available in two different strengths: 2 mg and 4 mg. The recommended dosage for rheumatoid arthritis is 2 mg once-daily oral, as monotherapy or in combination. Baricitinib is generally considered a safe and well-tolerated therapy. However, some side effects including severe infections (such as upper respiratory tract infections, urinary tract infections, and herpes zoster infections) have been reported, likely due to its immunosuppressive properties. Moreover, like the other JAKi, baricitinib is associated with bone marrow suppression and hematological abnormalities, including anemia, neutropenia, and lymphopenia. Other frequently observed adverse effects include acne vulgaris and headaches (288).

### 5.2 Second Generation Janus Kinase Inhibitors

Second-generation JAKi, such as filgotinib, itacitinib, and upadacitinib (anti JAK1), fedratinib (anti JAK2), and deucravacitinib (anti TYK2) offer greater target specificity and may provide a lower risk for hematological abnormalities and viral infections. These molecules are generally well-tolerated, with safety and efficacy currently under investigation.

• ***Upadacitinib***

Upadacitinib, taken at 15 mg once daily, is a JAK1 inhibitor approved to treat rheumatoid arthritis, psoriatic arthritis, atopic dermatitis and moderate-to-severe active ulcerative colitis (289).

• ***Filgotinib***

Filgotinib, administred at 200 mg once daily, is a selective, ATP-competitive JAK1 inhibitor approved in September 2020 by the EMA and in Japan for treating moderate to severe rheumatoid arthritis in adults. It is also being evaluated for the treatment of Crohn’s disease, ulcerative colitis, and psoriatic arthritis (290).

• ***Fedratinib***

Fedratinib is a competitive inhibitor of JAK2, BRD4, and FLT3, approved by the FDA in 2019 for treating intermediate or high-risk primary or secondary myelofibrosis (291).

• ***Deucravacitinib***

Deucravacitinib is the first selective allosteric inhibitor targeting the TYK2 pseudokinase domain, effectively blocking IL-12, IL-23, and IFN signaling pathways. The FDA approved its use for treating patients with moderate-to-severe psoriasis (292).

### 5.3 Safety and monitoring strategies for JAK inhibitors

Although JAKi have demonstrated significant clinical efficacy across a range of immune-mediated disorders and hematologic malignancies, several limitations remain. Resistance to therapy can emerge through secondary mutations or compensatory signaling pathways, reducing long-term effectiveness. In addition, incomplete selectivity may lead to off-target effects, raising concerns about safety profiles. The long-term use of JAK inhibitors is also associated with potential adverse events, including malignancies and cardiovascular complications, underscoring the importance of careful monitoring.

Given the risk of hematologic, hepatic, metabolic, and infectious adverse events, patients treated with JAKi should undergo systematic laboratory monitoring before and periodically after the treatment (293, 294). Baseline assessments should include a complete blood count with differential to identify pre-existing cytopenia (anemia, neutropenia, lymphopenia), as JAKi can exacerbate these abnormalities. Also, liver enzymes (transaminases) should be checked prior to initiation due to the risk of hepatotoxicity, particularly in patients receiving concomitant hepatotoxic medications. Renal function (estimated glomerular filtration rate (eGFR)) is also recommended at baseline, as certain JAKi require dose reduction in patients with moderate to severe renal impairment. A fasting lipid profile (low-density lipoprotein cholesterol (LDL), high-density lipoprotein cholesterol (HDL), and triglycerides (TG)) should be evaluated, as certain JAKi are associated with increases in both LDL and HDL cholesterol, typically within the first 12 weeks of treatment initiation. Screening for latent tuberculosis is essential before starting therapy, given the increased risk of reactivation. Additional baseline infectious work-up may include hepatitis B and C serologies, and HIV testing where clinically indicated.

Follow-up monitoring (4–12 weeks after initiation, then every 3–6 months) should include repeat assessments of complete blood count, liver enzymes, lipid profile, and renal function. These evaluations should be conducted regularly throughout treatment, with increased frequency in the presence of laboratory abnormalities or clinical symptoms. Particular attention should be given to elderly patients and those with baseline organ dysfunction.

These challenges underscore the need for ongoing optimization of JAKi design, patient stratification strategies, and combination approaches to maximize therapeutic benefit while minimizing risks.

## 6 Translational and interdisciplinary perspectives on JAK-STAT dysregulation

Building on knowledge from JAK-STAT dysregulation in IEIs, hematologic malignancies, and therapeutic applications, it becomes important to explore their relevance in broader disease settings.

Beyond their role in driving monogenic immune disorders, insights from JAK–STAT pathway dysregulation in IEIs offer valuable perspectives on broader immune conditions including hyperinflammatory states. As outlined above, LOF mutations typically impair host defense and increase susceptibility to infections, whereas GOF mutations, particularly in STAT1 and STAT3, are linked to immune dysregulation and hyperinflammation. These clinical observations mirror the pathophysiological mechanisms underlying acquired hyperinflammatory disorders thereby supporting the potential use of JAK inhibitors (such as ruxolitinib or baricitinib) in hyperinflammatory syndromes like HLH or the cytokine storm associated with COVID-19 (295, 296). Such examples illustrate how mechanistic insights from IEIs can be applied to develop targeted therapies that extend well beyond their original scope.

Similar translational lessons emerge from oncology, where dysregulated JAK-STAT signaling, first characterized in hematologic malignancies, provides a framework for understanding and targeting solid tumors. Indeed, aberrant JAK-STAT signaling also contributes to the pathogenesis of several solid tumors (including breast, colorectal, prostate, and pancreatic cancers) by driving proliferation, survival, angiogenesis, and immune evasion (297, 298). Notably, STAT3 and STAT5 are frequently overactivated in solid tumors, acting as potent oncogenic drivers by inducing pro-survival genes (BCL-2, BCL-XL, Survivin), promoting angiogenesis, and facilitating epithelial–mesenchymal transition. In contrast, STAT1 and STAT2 often play tumor-suppressive and immunoprotective roles. In parallel, JAK1 and JAK2 hyperactivation is commonly linked to cytokine-driven signaling that sustains tumor growth, while TYK2 shows dual behavior: supporting immune surveillance in some contexts but fostering immune evasion in others. Such mechanistic insights are increasingly being translated into therapeutic strategies tailored to solid tumors. For example, the rationale for targeting the JAK/STAT3 signaling axis in colorectal and pancreatic cancers builds on its established oncogenic role in hematologic settings, while the immunomodulatory functions of STAT1-STAT2 could inspire combination approaches with immune checkpoint inhibitors in solid tumors (297, 298).

Taken together, these interdisciplinary perspectives emphasize that unraveling JAK–STAT dysregulation in one disease context can inform therapeutic innovation across others, reinforcing the pathway’s central role as both a mechanistic driver and a therapeutic target in human pathology. Beyond their current applications, the JAK–STAT axis carries broad implications for precision medicine, serving as a cross-disciplinary platform for patient stratification, risk prediction, and treatment monitoring.

## 7 Conclusion

Over the past two decades, our understanding of JAK/STAT dysfunction has expanded significantly, primarily through the study of inborn errors of immunity and hematologic malignancies. Mutations within this pathway, whether germline or somatic, LOF or GOF, lead to a strikingly diverse range of clinical phenotypes: from severe combined immunodeficiencies to systemic autoimmunity, severe atopy, lymphoproliferation, and hematologic cancers. These conditions underscore the pleiotropic roles of JAK/STAT components in both innate and adaptive immunity.

Advances in molecular diagnostics, notably next-generation sequencing and flow cytometry, have allowed clinicians and researchers to delineate the functional consequences of specific mutations and to guide diagnosis and therapy. In parallel, therapeutic innovation has shifted from empirical immune suppression to rational, targeted modulation of signaling pathways. The clinical deployment of JAK inhibitors has validated the therapeutic potential of intervening in this pathway, particularly in myeloproliferative neoplasms, autoimmune diseases, and interferonopathies.

This review brings a unified perspective on these complex challenges by addressing both IEIs and hematologic malignancies within a shared JAK/STAT framework, a connection that is rarely emphasized in previous reviews. In addition, the inclusion of a dedicated section on diagnostic approaches offers practical guidance for clinicians, linking molecular understanding to real-world application. By integrating the latest insights into germline and somatic mutations, updated disease classifications, and currently available therapeutic options, we also provide a timely update in a rapidly evolving field.

Nonetheless, challenges remain. The overlapping phenotypes, variable penetrance, and mosaic presentations demand improved diagnostic algorithms and international registries. Long-term safety data on JAK inhibitors and other pathway modulators are needed, especially in pediatric populations and chronic disease settings. Furthermore, while precision medicine has taken root in hematology, its expansion into immune dysregulation disorders and rare IEIs requires coordinated multidisciplinary efforts.

Looking ahead, integrative approaches combining clinical, immunologic, and genomic profiling with artificial intelligence will likely redefine how JAK/STAT-related disorders are diagnosed and managed. The emergence of gene and RNA-based therapies opens new horizons for curative interventions, while a better understanding of cytokine biology will enable more refined immunomodulation.

In conclusion, the JAK/STAT pathway exemplifies the fusion of basic immunology, clinical medicine, and translational science. By bridging these domains and highlighting both recent discoveries and practical tools for diagnosis and treatment, our review aims to support ongoing efforts to advance precision immunology and improve patient outcomes across a wide disease spectrum.

## Glossary

BCP-ALL: B-cell Precursor Acute Lymphoblastic Leukemia
CCD: Coiled-Coil Domain
ChIP-seq: Chromatin Immunoprecipitation Sequencing
CMC: Chronic Mucocutaneous Candidiasis
CNV: Copy Number Variation
CRLF2: Cytokine Receptor-Like Factor 2
CSF3R: Colony-Stimulating Factor 3 Receptor
DBD: DNA-Binding Domain
DN: Dominant Negative
DNMT3A: DNA Methyltransferase 3A
ELISA: Enzyme-Linked Immunosorbent Assay
EPO: Erythropoietin
FERM: Four-point-one Ezrin Radixin Moesin
FLT3: FMS-like Tyrosine Kinase 3
GAS: Gamma-Activated Sequence
GH: Growth Hormone
GM-CSF: Granulocyte-Macrophage Colony-Stimulating Factor
HIES: Hyper IgE Syndrome
HLH: Hemophagocytic Lymphohistiocytosis
IEI: Inborn Error of Immunity
IFN: Interferon
IRF9: Interferon Regulatory Factor 9
IL: Interleukin
ISG: Interferon-Stimulated Gene
ISGF3: Interferon-Stimulated Gene Factor 3
JAK: Janus kinase
JAKi: JAK inhibitor
KMT2A: Lysine Methyltransferase 2A
KO: Knockout
LD: Linker Domain
MLNs: Mature Lymphoid Neoplasms
MPN: Myeloproliferative Neoplasm
MSMD: Mendelian Susceptibility to Mycobacterial Disease
Nmi: N-Myc interactor
NTD: N-terminal domain
PIAS: Protein Inhibitors of Activated STATs
PTP: Protein Tyrosine Phosphatase
PTPN2: Protein Tyrosine Phosphatase Non-receptor Type 2
qPCR: Quantitative PCR
RNA-seq: RNA sequencing
RT-PCR: Reverse Transcription PCR
SCID: Severe Combined Immunodeficiency
SH2: Src Homology 2
SHP1/2: Src Homology Phosphatase 1/2
shRNA: Short Hairpin RNA
siRNA: Small Interfering RNA
SMRT/N-CoR: Silencing Mediator of Retinoid and Thyroid hormone receptor/Nuclear Co-Repressor
SOCS: Suppressor of Cytokine Signaling
STAT: Signal Transducer and Activator of Transcription
T-ALL: T-cell Acute Lymphoblastic Leukemia
TC-PTP: T-cell Protein Tyrosine Phosphatase
TGP: Targeted Gene Panel
TPO: Thrombopoietin
TYK2: Tyrosine kinase 2
USP18: Ubiquitin-Specific Peptidase 18
WES: Whole Exome Sequencing
WGS: Whole Genome Sequencing;
