# Supplementary figures and images for: JAK/STAT in human diseases: a common axis in immunodeficiencies and hematological disorders
Source: Front Immunol. 2025 Dec 8;16:1669688. doi: 10.3389/fimmu.2025.1669688 (PMC12719510; doi:10.3389/fimmu.2025.1669688)

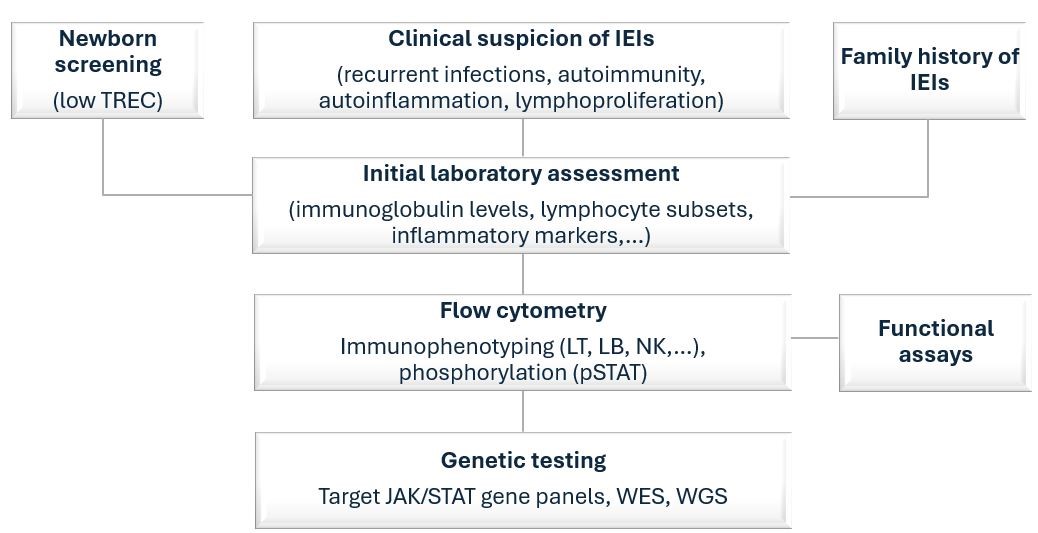

Supplement: Supplementary file 1 [file Image1.jpeg]
