# Supplementary material for: JAK/STAT in human diseases: a common axis in immunodeficiencies and hematological disorders
Source: Front Immunol. 2025 Dec 8;16:1669688. doi: 10.3389/fimmu.2025.1669688 (PMC12719510; doi:10.3389/fimmu.2025.1669688)
Supplement: Supplementary file 2 [file Table1.pdf]

TABLE S1. Literature-Reported Germline Variants in the STAT1 Gene.

N°	HGVS_p	Protein Domain	Type of mutation	Inheritance	Associated condition	Reference
1	p.(Asp65Asn)	NTD	GOF	AD	CMC	[1,2]
2	p.(Arg70His)	NTD	GOF	AD	CMC	[1,2]
3	p.(Arg70Pro)	NTD	GOF	AD	CMC	[1,2]
4	p.(Asn89Tyr)	NTD	GOF	AD	CMC	[1,2]
5	p.(Thr133Ala)	NTD	GOF	AD	CMC	[1,2]
6	p.(Asp151Glu)	CCD	GOF	AD	CMC	[1,2]
7	p.(Ile156Thr)	CCD	GOF	AD	CMC	[1,2]
8	p.(Ile160Phe)	CCD	GOF	AD	CMC	[1,2]
9	p.(Leu163Arg)	CCD	GOF	AD	CMC	[1,2]
10	p.(Asp165Gly)	CCD	GOF	AD	CMC	[1,2]
11	p.(Asp165His)	CCD	GOF	AD	CMC	[1,2]
12	p.(Gln167Glu)	CCD	GOF	AD	CMC	[1,2]
13	p.(Gln167His)	CCD	GOF	AD	CMC	[1,2]
14	p.(Gln167Pro)	CCD	GOF	AD	CMC	[1,2]
15	p.(Asp168Glu)	CCD	GOF	AD	CMC	[1,2]
16	p.(Tyr170Asn)	CCD	GOF	AD	CMC	[1,2]
17	p.(Asp171Asn)	CCD	GOF	AD	CMC	[1,2]
18	p.(Phe172Leu)	CCD	GOF	AD	CMC	[1,2]
19	p.(Cys174Arg)	CCD	GOF	AD	CMC	[1,2]
20	p.(Asn179Lys)	CCD	GOF	AD	CMC	[1,2]
21	p.(Met202Ile)	CCD	GOF	AD	CMC	[1,2]
22	p.(Met202Thr)	CCD	GOF	AD	CMC	[1,2]
23	p.(Met202Val)	CCD	GOF	AD	CMC	[1,2]
24	p.(Leu206His)	CCD	GOF	AD	CMC	[1,2]
25	p.(Leu206Pro)	CCD	GOF	AD	CMC	[1,2]
26	p.(Arg210Gly)	CCD	GOF	AD	CMC	[1,2]
27	p.(Arg210Ile)	CCD	GOF	AD	CMC	[1,2]
28	p.(Arg210Lys)	CCD	GOF	AD	CMC	[1,2]
29	p.(Glu235Ala)	CCD	GOF	AD	CMC	[1,2]
30	p.(Ala267Val)	CCD	GOF	AD	CMC	[1,2]
31	p.(Gln271Pro)	CCD	GOF	AD	CMC	[1,2]
32	p.(Arg274Gly)	CCD	GOF	AD	CMC	[1,2]
33	p.(Arg274Gln)	CCD	GOF	AD	CMC	[1,2]
34	p.(Arg274Trp)	CCD	GOF	AD	CMC	[1,2]
35	p.(Lys278Glu)	CCD	GOF	AD	CMC	[1,2]
36	p.(Leu283Phe)	CCD	GOF	AD	CMC	[1,2]
37	p.(Leu283Met)	CCD	GOF	AD	CMC	[1,2]
38	p.(Leu283Ser)	CCD	GOF	AD	CMC	[1,2]
39	p.(Glu284Lys)	CCD	GOF	AD	CMC	[1,2]
40	p.(Gln285Lys)	CCD	GOF	AD	CMC	[1,2]
41	p.(Gln285Arg)	CCD	GOF	AD	CMC	[1,2]
42	p.(Lys286Ile)	CCD	GOF	AD	CMC	[1,2]
43	p.(Tyr287Asp)	CCD	GOF	AD	CMC	[1,2]
44	p.(Tyr287His)	CCD	GOF	AD	CMC	[1,2]
45	p.(Thr288Ala)	CCD	GOF	AD	CMC	[1,2]
46	p.(Thr288Ile)	CCD	GOF	AD	CMC	[1,2]
47	p.(Thr288Pro)	CCD	GOF	AD	CMC	[1,2]
48	p.(Tyr289Cys)	CCD	GOF	AD	CMC	[1,2]
49	p.(Tyr289His)	CCD	GOF	AD	CMC	[1,2]
50	p.(Asp292Glu)	CCD	GOF	AD	CMC	[1,2]
51	p.(Asp292Asn)	CCD	GOF	AD	CMC	[1,2]
52	p.(Pro293Leu)	CCD	GOF	AD	CMC	[1,2]
53	p.(Pro293Ser)	CCD	GOF	AD	CMC	[1,2]
54	p.(Pro293Thr)	CCD	GOF	AD	CMC	[1,2]
55	p.(Ile294Thr)	CCD	GOF	AD	CMC	[1,2]
56	p.(Lys298Asn)	CCD	GOF	AD	CMC	[1,2]
57	p.(Leu301del)	CCD	GOF	AD	CMC	[1,2]
58	p.(Arg321Gly)	DBD	GOF	AD	CMC	[1,2]
59	p.(Arg321Ser)	DBD	GOF	AD	CMC	[1,2]
60	p.(Cys324Phe)	DBD	GOF	AD	CMC	[1,2]
61	p.(Cys324Arg)	DBD	GOF	AD	CMC	[1,2]
62	p.(Met325Lys)	DBD	GOF	AD	CMC	[1,2]
63	p.(His328Arg)	DBD	GOF	AD	CMC	[1,2]

64	p.(Pro329Leu)	DBD	GOF	AD	CMC	[1,2]
65	p.(Gln330Lys)	DBD	GOF	AD	CMC	[1,2]
66	p.(Lys344Glu)	DBD	GOF	AD	CMC	[1,2]
67	p.(Leu351Phe)	DBD	GOF	AD	CMC	[1,2]
68	p.(Glu353Lys)	DBD	GOF	AD	CMC	[1,2]
69	p.(Leu354Met)	DBD	GOF	AD	CMC	[1,2]
70	p.(Leu354Val)	DBD	GOF	AD	CMC	[1,2]
71	p.(Asn355Asp)	DBD	GOF	AD	CMC	[1,2]
72	p.(Asn357Asp)	DBD	GOF	AD	CMC	[1,2]
73	p.(Leu358Phe)	DBD	GOF	AD	CMC	[1,2]
74	p.(Leu358Trp)	DBD	GOF	AD	CMC	[1,2]
75	p.(Glu370Asp)	DBD	GOF	AD	CMC	[1,2]
76	p.(Gly384Cys)	DBD	GOF	AD	CMC	[1,2]
77	p.(Gly384Asp)	DBD	GOF	AD	CMC	[1,2]
78	p.(Thr385Lys)	DBD	GOF	AD	CMC	[1,2]
79	p.(Thr385Met)	DBD	GOF	AD	CMC	[1,2]
80	p.(Thr387Ala)	DBD	GOF	AD	CMC	[1,2]
81	p.(Lys388Glu)	DBD	GOF	AD	CMC	[1,2]
82	p.(Val389Ala)	DBD	GOF	AD	CMC	[1,2]
83	p.(Val389Leu)	DBD	GOF	AD	CMC	[1,2]
84	p.(Met390Ile)	DBD	GOF	AD	CMC	[1,2]
85	p.(Met390Thr)	DBD	GOF	AD	CMC	[1,2]
86	p.(Met392Thr)	DBD	GOF	AD	CMC	[1,2]
87	p.(Asn397Asp)	DBD	GOF	AD	CMC	[1,2]
88	p.(Leu400Gln)	DBD	GOF	AD	CMC	[1,2]
89	p.(Leu400Val)	DBD	GOF	AD	CMC	[1,2]
90	p.(Phe404Tyr)	DBD	GOF	AD	CMC	[1,2]
91	p.(Thr419Arg)	DBD	GOF	AD	CMC	[1,2]
92	p.(Thr437Ile)	DBD	GOF	AD	CMC	[1,2]
93	p.(Thr437Asn)	DBD	GOF	AD	CMC	[1,2]
94	p.(Ser466Arg)	DBD	GOF	AD	CMC	[1,2]
95	p.(Asp517Gly)	LD	GOF	AD	CMC	[1,2]
96	p.(Cys543Arg)	LD	GOF	AD	CMC	[1,2]
97	p.(Glu545Lys)	LD	GOF	AD	CMC	[1,2]
98	p.(Asn574His)	LD	GOF	AD	CMC	[1,2]
99	p.(Asn574Ile)	LD	GOF	AD	CMC	[1,2]
100	p.(His629Tyr)	SH2D	GOF	AD	CMC	[1,2]
101	p.(Val653Ile)	SH2D	GOF	AD	CMC	[1,2]
102	p.(Asn658Ser)	SH2D	GOF	AD	CMC	[1,2]
103	p.(Glu705Val)	TAD	GOF	AD	CMC	[1,2]
104	p.(Ser708Phe)	TAD	GOF	AD	CMC	[1,2]
105	p.(Glu711Gln)	TAD	GOF	AD	CMC	[1,2]
106	p.(Thr720Ile)	TAD	GOF	AD	CMC	[1,2]
107	p.Gln124His	NTD	LOF	AR complete	Viral/ mycobacterial infections	[3,4]
108	Ex5del	NTD	LOF	AR complete	Viral/ mycobacterial infections	[3,4]
109	c.128+2T>G	NTD	LOF	AR complete	Viral/ mycobacterial infections	[3,4]
110	c.542-8 A>G	CCD	LOF	AR complete	Viral/ mycobacterial infections	[3,4]
111	c.1757_1758delAG	SH2D	LOF	AR complete	Viral/ mycobacterial infections	[3,4]
112	p.Leu600Pro	SH2D	LOF	AR complete	Viral/ mycobacterial infections	[3,4]
113	c.1928insA	SH2D	LOF	AR complete	Viral/ mycobacterial infections	[3,4]
114	p.Ala46Thr	NTD	LOF	AR partial	Viral/ mycobacterial infections	[3,4]
115	p.Lys201Asn	CCD	LOF	AR partial	Viral/ mycobacterial infections	[3,4]
116	p.Lys211Arg	CCD	LOF	AR partial	Viral/ mycobacterial infections	[3,4]
117	p.Pro696Ser	TSD	LOF	AR partial	Viral/ mycobacterial infections	[3,4]
118	p.Glu157Lys	CCD	LOF	AD	MSMD	[3,4]
119	p.Gly250Ala	CCD	LOF	AD	MSMD	[3,4]
120	p.Gly250Glu	CCD	LOF	AD	MSMD	[3,4]
121	p.Glu320Gln	DBD	LOF	AD	MSMD	[3,4]
122	p.Gln463His	DBD	LOF	AD	MSMD	[3,4]
123	p.Lys637Glu	SH2D	LOF	AD	MSMD	[3,4]
124	p.Met654Lys	SH2D	LOF	AD	MSMD	[3,4]
125	p.Lys673Arg	SH2D	LOF	AD	MSMD	[3,4]
126	p.Tyr701Cys	TSD	LOF	AD	MSMD	[3,4]
127	p.Leu706Ser	TSD	LOF	AD	MSMD	[3,4]

N-terminal domain (NTD), Coiled-coil domain (CCD), Tail segment domain (TSD), DNA-binding domain (DBD), Transactivation domain (TAD), SH2 domain (SH2D), Linker domain (L), Chronic mucocutaneous candidiasis (CMC); Mendelian susceptibility to mycobacterial disease (MSMD)

REFERENCES

1. Liu L, Okada S, Kong X-F, Kreins AY, Cypowyj S, Abhyankar A, et al. Gain-of-function human STAT1 mutations impair IL-17 immunity and underlie chronic mucocutaneous candidiasis. Journal of Experimental Medicine. 2011;208:1635–48. https://doi.org/10.1084/jem.20110958

2. L van de VF, S PT, Alexander H, P SS, B JLA, Christian G, et al. STAT1 Mutations in Autosomal Dominant Chronic Mucocutaneous Candidiasis. New England Journal of Medicine. Massachusetts Medical Society; 2025;365:54–61. https://doi.org/10.1056/NEJMoa1100102

3. Mizoguchi Y, Okada S. Inborn errors of STAT1 immunity. Curr Opin Immunol. 2021;72:59–64. https://doi.org/https://doi.org/10.1016/j.coi.2021.02.009

4. Zhang W, Chen X, Gao G, Xing S, Zhou L, Tang X, et al. Clinical Relevance of Gain- and Loss-of-Function Germline Mutations in STAT1: A Systematic Review. Front Immunol. 2021;Volume 12-2021. https://doi.org/10.3389/fimmu.2021.654406

TABLE S2. Literature-Reported Germline Variants in the STAT2 Gene.

N°	HGVS_c (NM_005419.3)	HGVS_P	Protein Domain	Type of mutation	Inheritance	Associated condition	Reference
1	c.381+5G>C (splice variant)	Complete absence of STAT2 protein	Complete absence of STAT2 protein	LOF	AR	Severe viral infections	[1]
2	c.820C>T	p.Gln274Ter	Coiled-coil domain	LOF	AR	Severe viral infections	[1]
3	c.988C>T	p.Arg330Ter	DNA-binding domain	LOF	AR	Severe viral infections	[1]
4	c.1209+1delG	Splice variant (delEx13)	Linker domain	LOF	AR (compound heterozygous)	Severe viral infections	[1]
5	c.1528C>T	p.Arg510Ter	SH2 domain	LOF	AR	Severe viral infections	[1]
6	c.1576G>A	p.Val526Ile (predicted deleterious)	SH2 domain	LOF	AR	Severe viral infections	[1]
7	c.1836C>A	p.Cys612Ter	SH2 domain	LOF	AR	Severe viral infections	[1]
8	c.1883_1884del	p.Val628fs*14	SH2 linker	LOF	AR	Severe viral infections	[1]
9	c.1999C>T	p.Arg667Ter	Transactivation domain (TAD)	LOF	AR	Severe viral infections	[1]
10	del(5′US–In8) [chr12:56360796–56352109]	Complete absence of STAT2 protein	Complete absence of STAT2 protein	LOF	AR	Severe viral infections	[1]
11	del(Ex5–In19) [chr12:56355504–56348082]	Complete absence of STAT2 protein	Complete absence of STAT2 protein	LOF	AR (compound heterozygous)	Severe viral infections	[1]
12	c.656C>T	p.Ala219Val	Coiled-Coil Domain (CCD)	Loss of negative regulation	AR	type I interferonopathy	[2]
13	c.442C>T	p.Arg148Trp	Coiled-Coil Domain (CCD)	Loss of negative regulation	AR	type I interferonopathy	[3]
14	c.443G>A	p.Arg148Gln	Coiled-Coil Domain (CCD)	Loss of negative regulation	AR	type I interferonopathy	[4]

Autosomal recessive (AR), Loss of function (LOF).

REFERENCES

1. Bucciol G, Moens L, Ogishi M, Rinchai D, Matuozzo D, Momenilandi M, et al. Human inherited complete STAT2 deficiency underlies inflammatory viral diseases. Journal of Clinical Investigation. American Society for Clinical Investigation; 2023;133. https://doi.org/10.1172/JCI168321

2. Zhu G, Badonyi M, Franklin L, Seabra L, Rice GI, Anne-Boland-Auge, et al. Type I Interferonopathy due to a Homozygous Loss-of-Inhibitory Function Mutation in STAT2. J Clin Immunol. 2023;43:808–18. https://doi.org/10.1007/s10875-023-01445-3

3. Duncan CJA, Thompson BJ, Chen R, Rice GI, Gothe F, Young DF, et al. Severe type I interferonopathy and unrestrained interferon signaling due to a homozygous germline mutation in STAT2. Sci Immunol. American Association for the Advancement of Science; 2019;4:eaav7501. https://doi.org/10.1126/sciimmunol.aav7501

4. Gruber C, Martin-Fernandez M, Ailal F, Qiu X, Taft J, Altman J, et al. Homozygous STAT2 gain-of-function mutation by loss of USP18 activity in a patient with type I interferonopathy. Journal of Experimental Medicine. 2020;217:e20192319. https://doi.org/10.1084/jem.20192319

TABLE S3. Literature-Reported Germline and Somatic Variants in the STAT3 Gene.

HGVS	Protein Domain	Type of mutation	Inheritance	Condition	Reference
p.Arg382Trp	DBD	Germline, LOF (DN)	AD	HIES	[1]
p.Arg382Gln	DBD	Germline, LOF (DN)	AD	HIES	[1]
p.Arg382Leu	DBD	Germline, LOF (DN)	AD	HIES	[1]
p.Phe384Leu	DBD	Germline, LOF (DN)	AD	HIES	[1]
p.Phe384Ser	DBD	Germline, LOF (DN)	AD	HIES	[1]
p.Arg423Gln	DBD	Germline, LOF (DN)	AD	HIES	[1]
p.Val463del	DBD	Germline, LOF (DN)	AD	HIES	[1]
p.Ser465Ala	DBD	Germline, LOF (DN)	AD	HIES	[1]
p.Ser611Asn	SH2	Germline, LOF (DN)	AD	HIES	[1]
p.Phe621Val	SH2	Germline, LOF (DN)	AD	HIES	[1]
p.Thr622Ile	SH2	Germline, LOF (DN)	AD	HIES	[1]
p.Val637Met	SH2	Germline, LOF (DN)	AD	HIES	[1]
p.Val637Leu	SH2	Germline, LOF (DN)	AD	HIES	[1]
p.Pro639Ala	SH2	Germline, LOF (DN)	AD	HIES	[1]
p.Gln644del	SH2	Germline, LOF (DN)	AD	HIES	[1]
p.Asn647Asp	SH2	Germline, LOF (DN)	AD	HIES	[1]
p.Glu652Lys	SH2	Germline, LOF (DN)	AD	HIES	[1]
p.Tyr657Cys	SH2	Germline, LOF (DN)	AD	HIES	[1]
p.Arg382Trp	DBD	Germline, LOF (DN)	AD	HIES	[2]
p.Arg382Gln	DBD	Germline, LOF (DN)	AD	HIES	[2]
p.Arg382Leu	DBD	Germline, LOF (DN)	AD	HIES	[2]
p.Phe384Ser	DBD	Germline, LOF (DN)	AD	HIES	[2]
p.Phe384Leu	DBD	Germline, LOF (DN)	AD	HIES	[2]
p.Arg423Gln	DBD	Germline, LOF (DN)	AD	HIES	[2]
p.Val463del	DBD	Germline, LOF (DN)	AD	HIES	[2]
p.Ser465Ala	DBD	Germline, LOF (DN)	AD	HIES	[2]
p.Lys531Gln	LD	Germline, LOF (DN)	AD	HIES	[2]
p.Leu571Phe	SH2-linker	Germline, LOF (DN)	AD	HIES	[2]
p.Tyr575Asp	SH2-linker	Germline, LOF (DN)	AD	HIES	[2]
p.Val637Met	SH2	Germline, LOF (DN)	AD	HIES	[2]
p.Val637Leu	SH2	Germline, LOF (DN)	AD	HIES	[2]
p.Pro639Ala	SH2	Germline, LOF (DN)	AD	HIES	[2]
p.Gln644del	SH2	Germline, LOF (DN)	AD	HIES	[2]
p.Asn647Asp	SH2	Germline, LOF (DN)	AD	HIES	[2]
p.Glu652Lys	SH2	Germline, LOF (DN)	AD	HIES	[2]
p.Tyr657Cys	SH2	Germline, LOF (DN)	AD	HIES	[2]
p.Ile711Phe	TAD	Germline, LOF (DN)	AD	HIES	[2]
p.Met660Val	SH2	Germline, LOF (DN)	AD	HIES	[2]
p.Ser465Pro	DBD	Germline, LOF (DN)	AD	HIES	[2]
p.His58Tyr	CCD	Germline, LOF (DN)	AD	HIES	[3]
p.Cys328_Pro330dup	DBD	Germline, LOF (DN)	AD	HIES	[3]
p.Gly342Asp	DBD	Germline, LOF (DN)	AD	HIES	[3]
p.Asp371_Gly380del	DBD	Germline, LOF (DN)	AD	HIES	[3]
p.Asp371_Gly380del	DBD	Germline, LOF (DN)	AD	HIES	[3]
p.Asp371_Gly380del	DBD	Germline, LOF (DN)	AD	HIES	[3]
p.Asp371_Gly380del	DBD	Germline, LOF (DN)	AD	HIES	[3]
p.Arg382Trp	DBD	Germline, LOF (DN)	AD	HIES	[3]
p.Arg382Leu	DBD	Germline, LOF (DN)	AD	HIES	[3]
p.Arg382Gln	DBD	Germline, LOF (DN)	AD	HIES	[3]
p.Phe384Leu	DBD	Germline, LOF (DN)	AD	HIES	[3]
p.Thr389Ile	DBD	Germline, LOF (DN)	AD	HIES	[3]
p.Arg423Gln	DBD	Germline, LOF (DN)	AD	HIES	[3]
p.Val463del	DBD	Germline, LOF (DN)	AD	HIES	[3]
p.Asn466Asp	DBD	Germline, LOF (DN)	AD	HIES	[3]
p.Asn466Ser	DBD	Germline, LOF (DN)	AD	HIES	[3]
p.Asn466Thr	DBD	Germline, LOF (DN)	AD	HIES	[3]
p.Asn466Lys	DBD	Germline, LOF (DN)	AD	HIES	[3]
p.Gln469His	DBD	Germline, LOF (DN)	AD	HIES	[3]
p.Lys591Glu	SH2	Germline, LOF (DN)	AD	HIES	[3]
p.Thr622Ile	SH2	Germline, LOF (DN)	AD	HIES	[3]
p.Ser636Tyr	SH2	Germline, LOF (DN)	AD	HIES	[3]
p.Val637Met	SH2	Germline, LOF (DN)	AD	HIES	[3]
p.Val637Ala	SH2	Germline, LOF (DN)	AD	HIES	[3]
p.Pro639Ser	SH2	Germline, LOF (DN)	AD	HIES	[3]
p.Tyr657Cys	SH2	Germline, LOF (DN)	AD	HIES	[3]

p.Ser668Phe	SH2	Germline, LOF (DN)	AD	HIES	[3]
p.Thr708Ser	TAD	Germline, LOF (DN)	AD	HIES	[3]
p.Phe710Cys	TAD	Germline, LOF (DN)	AD	HIES	[3]
p.Thr714Ala	TAD	Germline, LOF (DN)	AD	HIES	[3]
c.2144+1G>A, p. ?	TAD	Germline, LOF (DN)	AD	HIES	[3]
p.Gly342Asp	DBD	Germline, LOF (DN)	AD	HIES	[4]
p.Arg382Trp	DBD	Germline, LOF (DN)	AD	HIES	[4]
p.Arg382Gln	DBD	Germline, LOF (DN)	AD	HIES	[4]
p.Val432Leu	DBD	Germline, LOF (DN)	AD	HIES	[4]
p.Val463del	DBD	Germline, LOF (DN)	AD	HIES	[4]
p.Ser465Ala	DBD	Germline, LOF (DN)	AD	HIES	[4]
p.Asn466Ser	DBD	Germline, LOF (DN)	AD	HIES	[4]
p.Asn567Asp	LD	Germline, LOF (DN)	AD	HIES	[4]
p.Lys591Glu	SH2	Germline, LOF (DN)	AD	HIES	[4]
p.Val637Met	SH2	Germline, LOF (DN)	AD	HIES	[4]
p.Val637Ala	SH2	Germline, LOF (DN)	AD	HIES	[4]
p.Pro639Leu	SH2	Germline, LOF (DN)	AD	HIES	[4]
p.Met660Ala	SH2	Germline, LOF (DN)	AD	HIES	[4]
p.Phe710Cys	TAD	Germline, LOF (DN)	AD	HIES	[4]
p.Phe621Leu	TAD	Germline, LOF (DN)	AD	HIES	[5]
p.His437Gln	DBD	Germline, LOF (DN)	AD	HIES	[5]
p.Arg382Trp	DBD	Germline, LOF (DN)	AD	HIES	[5]
p.Arg382Pro	DBD	Germline, LOF (DN)	AD	HIES	[5]
p.Arg609Thr	SH2	Germline, LOF (DN)	AD	HIES	[5]
p.Arg609Ser	SH2	Germline, LOF (DN)	AD	HIES	[5]
p.Val637Met	SH2	Germline, LOF (DN)	AD	HIES	[5]
p.His332Asn	CCD	Germline, LOF (DN)	AD	HIES	[5]
p.Gly421Arg	DBD	Germline, LOF (DN)	AD	HIES	[5]
p.Arg382Gln	DBD	Germline, LOF (DN)	AD	HIES	[6]
p.Arg382Trp	DBD	Germline, LOF (DN)	AD	HIES	[6]
p.Val637Met	SH2	Germline, LOF (DN)	AD	HIES	[6]
p.Asn466Thr	DBD	Germline, LOF (DN)	AD	HIES	[6]
p.Tyr657Ter	SH2	Germline, LOF (DN)	AD	HIES	[6]
p.Arg18fs*34	N-terminal	Germline, LOF (DN)	AD	HIES	[6]
p.Phe493Leufs*508	DBD	Germline, LOF (DN)	AD	HIES	[6]
p.Phe174Ser	CCD	Germline, LOF (DN)	AD	HIES	[7]
p.Val637Met	SH2	Germline, LOF (DN)	AD	HIES	[7]
p.Arg382Gln	DBD	Germline, LOF (DN)	AD	HIES	[7]
p.Asn567Asp	LD	Germline, LOF (DN)	AD	HIES	[7]
p.Leu404Serfs*8	DBD	Germline, LOF (DN)	AD	HIES	[7]
p.Asp661Val	SH2	Germline, LOF (DN)	AD	HIES	[7]
p.Tyr657Cys	SH2	Germline, LOF (DN)	AD	HIES	[7]
p.Met329Lys	DBD	Germline, LOF (DN)	AD	HIES	[7]
p.Val637Met	SH2	Germline, LOF (DN)	AD	HIES	[7]
p.Arg382Trp	DBD	Germline, LOF (DN)	AD	HIES	[7]
p.Gly380Arg	DBD	Germline, LOF (DN)	AD	HIES	[7]
p.Lys340Gln	DBD	Germline, LOF (DN)	AD	HIES	[7]
p.Arg382Gln	DBD	Germline, LOF (DN)	AD	HIES	[7]
p.Met660Thr	SH2	Germline, LOF (DN)	AD	HIES	[7]
p.Thr714Ile	TAD	Germline, LOF (DN)	AD	HIES	[7]
p.Arg518Ter	LD	Germline, LOF (DN)	AD	HIES	[7]
p.Arg423Gln	DBD	Germline, LOF (DN)	AD	HIES	[7]
p.Ile467Phe (I467F)	DBD	Germline, LOF (DN)	AD	HIES	[7]
p.Glu434Asp	DBD	Germline, LOF (DN)	AD	HIES	[7]
p.Arg382Trp	DBD	Germline, LOF (DN)	AD	HIES	[8]
p.Arg382Gln	DBD	Germline, LOF (DN)	AD	HIES	[8]
p.Val432Ile	DBD	Germline, LOF (DN)	AD	HIES	[8]
p.Tyr469Cys	DBD	Germline, LOF (DN)	AD	HIES	[8]
p.Glu609Asp	SH2	Germline, LOF (DN)	AD	HIES	[8]
p.Thr620Arg	SH2	Germline, LOF (DN)	AD	HIES	[8]
p.Asn621Lys	SH2	Germline, LOF (DN)	AD	HIES	[8]
p.Val637Met	SH2	Germline, LOF (DN)	AD	HIES	[8]
p.Lys638Arg	SH2	Germline, LOF (DN)	AD	HIES	[8]
p.Tyr657Ser	SH2	Germline, LOF (DN)	AD	HIES	[8]
p.Tyr705His	TAD	Germline, LOF (DN)	AD	HIES	[8]
c.1140-2A>C	Absence of protein? (Aberrant splicing)	Germline, LOF (DN)	AD	HIES	[9]
c.1140-2A>G	Absence of protein? (Aberrant splicing)	Germline, LOF (DN)	AD	HIES	[9]

p.Ala13LeufsTer41	Early frameshift causing premature stop and loss of STAT3 function.	Germline, LOF (DN)	AD	HIES	[9]
c.1140-3C>G	splice site affected	Germline, LOF (DN)	AD	HIES	[9]
p.Phe493LeufsTer16	Early truncation → complete loss of STAT3 function	Germline, LOF (DN)	AD	HIES	[9]
p.Tyr657*	Frameshift in DBD → abolishes DNA binding and downstream structure	Germline, LOF (DN)	AD	HIES	[9]
p.Gln125*	Frameshift within LD→ loss of SH2 and TAD	Germline, LOF (DN)	AD	HIES	[9]
p.Asp371LeufsTer14	Truncation in LD → removes SH2 and TAD	Germline, LOF (DN)	AD	HIES	[9]
p.Arg518*	Truncation near SH2 junction → loss of SH2 and TAD	Germline, LOF (DN)	AD	HIES	[9]
p.Trp562*	Truncation in SH2 → disrupts dimerization and TAD	Germline, LOF (DN)	AD	HIES	[9]
p.Gln633*	Stop within SH2 → loss of SH2 and TAD	Germline, LOF (DN)	AD	HIES	[9]
p.Asp698GlufsTer6	TAD	Germline, LOF (DN)	AD	HIES	[9]
p.His58Tyr	N-term	Germline, LOF (DN)	AD	HIES	[9]
p.Arg84Gln	N-term	Germline, LOF (DN)	AD	HIES	[9]
p.His332Arg	DBD	Germline, LOF (DN)	AD	HIES	[9]
p.His332Leu	DBD	Germline, LOF (DN)	AD	HIES	[9]
p.His332Tyr	DBD	Germline, LOF (DN)	AD	HIES	[9]
p.His332Pro	DBD	Germline, LOF (DN)	AD	HIES	[9]
p.Arg335Trp	DBD	Germline, LOF (DN)	AD	HIES	[9]
p.Lys340Gln	DBD	Germline, LOF (DN)	AD	HIES	[9]
p.Lys340Glu	DBD	Germline, LOF (DN)	AD	HIES	[9]
p.Thr341Asn	DBD	Germline, LOF (DN)	AD	HIES	[9]
p.Gly342Asp	DBD	Germline, LOF (DN)	AD	HIES	[9]
p.Val343Leu	DBD	Germline, LOF (DN)	AD	HIES	[9]
p.Val343Phe	DBD	Germline, LOF (DN)	AD	HIES	[9]
p.Pro356Arg	DBD	Germline, LOF (DN)	AD	HIES	[9]
p.Asp374Gly	DBD	Germline, LOF (DN)	AD	HIES	[9]
p.Arg382Gly	DBD	Germline, LOF (DN)	AD	HIES	[9]
p.Phe384Cys	DBD	Germline, LOF (DN)	AD	HIES	[9]
p.Thr389Ile	DBD	Germline, LOF (DN)	AD	HIES	[9]
p.Asn395Tyr	DBD	Germline, LOF (DN)	AD	HIES	[9]
p.Thr412Ala	DBD	Germline, LOF (DN)	AD	HIES	[9]
p.Thr412Ser	DBD	Germline, LOF (DN)	AD	HIES	[9]
p.Asn425Tyr	DBD	Germline, LOF (DN)	AD	HIES	[9]
p.Val432Met	DBD	Germline, LOF (DN)	AD	HIES	[9]
p.Val432Leu	DBD	Germline, LOF (DN)	AD	HIES	[9]
p.His437Pro	DBD	Germline, LOF (DN)	AD	HIES	[9]
p.His437Tyr	DBD	Germline, LOF (DN)	AD	HIES	[9]
p.Val463Glu	DBD	Germline, LOF (DN)	AD	HIES	[9]
p.Ser465Phe	DBD	Germline, LOF (DN)	AD	HIES	[9]
p.Asn466Ile	LD	Germline, LOF (DN)	AD	HIES	[9]
p.Asn466Lys	LD	Germline, LOF (DN)	AD	HIES	[9]
p.Asn466Thr	LD	Germline, LOF (DN)	AD	HIES	[9]
p.Asn466Ser	LD	Germline, LOF (DN)	AD	HIES	[9]
p.Asn466Asp	LD	Germline, LOF (DN)	AD	HIES	[9]
p.Ile467Phe	LD	Germline, LOF (DN)	AD	HIES	[9]
p.Gln469His	LD	Germline, LOF (DN)	AD	HIES	[9]
p.Gln469Arg	LD	Germline, LOF (DN)	AD	HIES	[9]
p.Asn472Asp	LD	Germline, LOF (DN)	AD	HIES	[9]
p.Ser476Phe	LD	Germline, LOF (DN)	AD	HIES	[9]
p.Trp479Cys	LD	Germline, LOF (DN)	AD	HIES	[9]
p.Lys531Glu	LD	Germline, LOF (DN)	AD	HIES	[9]
p.Lys531Asn	LD	Germline, LOF (DN)	AD	HIES	[9]
p.Asn567Asp	LD	Germline, LOF (DN)	AD	HIES	[9]
p.Ile568Met	SH2	Germline, LOF (DN)	AD	HIES	[9]

p.Ile568Phe	SH2	Germline, LOF (DN)	AD	HIES	[9]
p.Lys591Glu	SH2	Germline, LOF (DN)	AD	HIES	[9]
p.Lys591Met	SH2	Germline, LOF (DN)	AD	HIES	[9]
p.Arg593Pro	SH2	Germline, LOF (DN)	AD	HIES	[9]
p.Glu594Lys	SH2	Germline, LOF (DN)	AD	HIES	[9]
p.Arg609Gly	SH2	Germline, LOF (DN)	AD	HIES	[9]
p.Ser611Asn	SH2	Germline, LOF (DN)	AD	HIES	[9]
p.Ser611Ile	SH2	Germline, LOF (DN)	AD	HIES	[9]
p.Ser611Gly	SH2	Germline, LOF (DN)	AD	HIES	[9]
p.Ser614Gly	SH2	Germline, LOF (DN)	AD	HIES	[9]
p.Lys615Glu	SH2	Germline, LOF (DN)	AD	HIES	[9]
p.Gly617Glu	SH2	Germline, LOF (DN)	AD	HIES	[9]
p.Gly617Val	SH2	Germline, LOF (DN)	AD	HIES	[9]
p.Gly618Asp	SH2	Germline, LOF (DN)	AD	HIES	[9]
p.Thr620Ser	SH2	Germline, LOF (DN)	AD	HIES	[9]
p.Thr620Ala	SH2	Germline, LOF (DN)	AD	HIES	[9]
p.Phe621Ile	SH2	Germline, LOF (DN)	AD	HIES	[9]
p.Phe621Val	SH2	Germline, LOF (DN)	AD	HIES	[9]
p.Phe621Ser	SH2	Germline, LOF (DN)	AD	HIES	[9]
p.Phe621Leu	SH2	Germline, LOF (DN)	AD	HIES	[9]
p.Trp623Leu	SH2	Germline, LOF (DN)	AD	HIES	[9]
p.Ser636Tyr	SH2	Germline, LOF (DN)	AD	HIES	[9]
p.Ser636Phe	SH2	Germline, LOF (DN)	AD	HIES	[9]
p.Val637Ala	SH2	Germline, LOF (DN)	AD	HIES	[9]
p.Val637Leu	SH2	Germline, LOF (DN)	AD	HIES	[9]
p.Glu638Gly	SH2	Germline, LOF (DN)	AD	HIES	[9]
p.Pro639Thr	SH2	Germline, LOF (DN)	AD	HIES	[9]
p.Pro639Gln	SH2	Germline, LOF (DN)	AD	HIES	[9]
p.Pro639Ser	SH2	Germline, LOF (DN)	AD	HIES	[9]
p.Lys642Glu	SH2	Germline, LOF (DN)	AD	HIES	[9]
p.Gln644Pro	SH2	Germline, LOF (DN)	AD	HIES	[9]
p.Leu645Gln	SH2	Germline, LOF (DN)	AD	HIES	[9]
p.Glu652Lys	SH2	Germline, LOF (DN)	AD	HIES	[9]
p.Tyr657Asn	SH2	Germline, LOF (DN)	AD	HIES	[9]
p.Lys658Glu	SH2	Germline, LOF (DN)	AD	HIES	[9]
p.Met660Thr	SH2	Germline, LOF (DN)	AD	HIES	[9]
p.Met660Arg	SH2	Germline, LOF (DN)	AD	HIES	[9]
p.Ile665Asn	SH2	Germline, LOF (DN)	AD	HIES	[9]
p.Ser668Tyr	SH2	Germline, LOF (DN)	AD	HIES	[9]
p.Ser668Phe	SH2	Germline, LOF (DN)	AD	HIES	[9]
p.Tyr705Asn	TAD	Germline, LOF (DN)	AD	HIES	[9]
p.Tyr705Cys	TAD	Germline, LOF (DN)	AD	HIES	[9]
p.Leu706Met	TAD	Germline, LOF (DN)	AD	HIES	[9]
p.Thr708Asn	TAD	Germline, LOF (DN)	AD	HIES	[9]
p.Thr708Ser	TAD	Germline, LOF (DN)	AD	HIES	[9]
p.Lys709Glu	TAD	Germline, LOF (DN)	AD	HIES	[9]
p.Phe710Cys	TAD	Germline, LOF (DN)	AD	HIES	[9]
p.Ile711Thr	TAD	Germline, LOF (DN)	AD	HIES	[9]
p.Ile711Val	TAD	Germline, LOF (DN)	AD	HIES	[9]
p.Cys712Arg	TAD	Germline, LOF (DN)	AD	HIES	[9]
p.Val713Met	TAD	Germline, LOF (DN)	AD	HIES	[9]
p.Val713Leu	TAD	Germline, LOF (DN)	AD	HIES	[9]
p.Thr714Ile	TAD	Germline, LOF (DN)	AD	HIES	[9]
p.Thr714Ala	TAD	Germline, LOF (DN)	AD	HIES	[9]
p.Ala744Val	TAD	Germline, LOF (DN)	AD	HIES	[9]
c.1110-2A>G	Absence of protein? (Aberrant splicing)	Germline, LOF (DN)	AD	HIES	[9]
c.1110-2A>C	Absence of protein? (Aberrant splicing)	Germline, LOF (DN)	AD	HIES	[9]
c.1110-3C>A	Absence of protein? (Aberrant splicing)	Germline, LOF (DN)	AD	HIES	[9]
c.1110-1 G>T	Absence of protein? (Aberrant splicing)	Germline, LOF (DN)	AD	HIES	[9]
c.1139+5 G>A	Absence of protein? (Aberrant splicing)	Germline, LOF (DN)	AD	HIES	[9]
c.1139+1G>T	Absence of protein? (Aberrant splicing)	Germline, LOF (DN)	AD	HIES	[9]
c.1281+1del	Absence of protein? (Aberrant splicing)	Germline, LOF (DN)	AD	HIES	[9]

c.1281-89C>T	Absence of protein? (Aberrant splicing)	Germline, LOF (DN)	AD	HIES	[9]
p.Asp427_Gly428ins17	DBD	Germline, LOF (DN)	AD	HIES	[9]
p.Lys340_Thr341delinsAsn	CCD	Germline, LOF (DN)	AD	HIES	[9]
p.Asp369_Leu370delinsGlu	DBD	Germline, LOF (DN)	AD	HIES	[9]
p.Val463del	LD	Germline, LOF (DN)	AD	HIES	[9]
p.Gln644del	SH2	Germline, LOF (DN)	AD	HIES	[9]
c.2101+2332_oSTAT: c.22571772del3933bp	Absence of protein? (Large deletion)	Germline, LOF (DN)	AD	HIES	[9]
p.Ser560del	LD	Germline, LOF (DN)	AD	HIES	[9]
p.Ile654del	SH2	Germline, LOF (DN)	AD	HIES	[9]
p.Ile654_Lys658del	SH2	Germline, LOF (DN)	AD	HIES	[9]
p.Gly656_Met660del	SH2	Germline, LOF (DN)	AD	HIES	[9]
p.His328_Glu330dup	CCD	Germline, LOF (DN)	AD	HIES	[9]
c.1139+2dup	Absence of protein? (Aberrant splicing)	Germline, LOF (DN)	AD	HIES	[9]
p.Thr708dup	TAD	Germline, LOF (DN)	AD	HIES	[9]
p.Arg103Trp	N-ter	Germline, GOF	AD	Immune dysregulation	[10]
p.Arg107Gln	N-ter	Germline, GOF	AD	Immune dysregulation	[10]
p.Gln125Glu	N-ter	Germline, GOF	AD	Immune dysregulation	[10]
p.Arg152Trp	CCD	Germline, GOF	AD	Immune dysregulation	[10]
p.Phe174Ser	CCD	Germline, GOF	AD	Immune dysregulation	[10]
p.Met206Arg	CCD	Germline, GOF	AD	Immune dysregulation	[10]
p.Arg246Gln	CCD	Germline, GOF	AD	Immune dysregulation	[10]
p.Leu260Pro	CCD	Germline, GOF	AD	Immune dysregulation	[10]
p.Arg278His	CCD	Germline, GOF	AD	Immune dysregulation	[10]
p.Gln280Pro	CCD	Germline, GOF	AD	Immune dysregulation	[10]
p.Glu286Gly	CCD	Germline, GOF	AD	Immune dysregulation	[10]
p.Glu286Ala	CCD	Germline, GOF	AD	Immune dysregulation	[10]
p.Leu287Val	CCD	Germline, GOF	AD	Immune dysregulation	[10]
p.Gln288Pro	CCD	Germline, GOF	AD	Immune dysregulation	[10]
p.Lys290Asn	CCD	Germline, GOF	AD	Immune dysregulation	[10]
p.Arg302Gln	CCD	Germline, GOF	AD	Immune dysregulation	[10]
p.Phe313Leu	CCD	Germline, GOF	AD	Immune dysregulation	[10]
p.Met329Lys	DBD	Germline, GOF	AD	Immune dysregulation	[10]
p.Met329Arg	DBD	Germline, GOF	AD	Immune dysregulation	[10]
p.Pro330Ser	DBD	Germline, GOF	AD	Immune dysregulation	[10]
p.Gln344His	DBD	Germline, GOF	AD	Immune dysregulation	[10]
p.Lys348Glu	DBD	Germline, GOF	AD	Immune dysregulation	[10]
p.Val353Phe	DBD	Germline, GOF	AD	Immune dysregulation	[10]
p.Lys392Arg	DBD	Germline, GOF	AD	Immune dysregulation	[10]
p.Val393Ala	DBD	Germline, GOF	AD	Immune dysregulation	[10]
p.Met394Thr	DBD	Germline, GOF	AD	Immune dysregulation	[10]
p.Asn401Asp	DBD	Germline, GOF	AD	Immune dysregulation	[10]
p.Glu415Leu	DBD	Germline, GOF	AD	Immune dysregulation	[10]

p.Glu415Lys	DBD	Germline, GOF	AD	Immune dysregulation	[10]
p.Gly419Arg	DBD	Germline, GOF	AD	Immune dysregulation	[10]
p.Asn420Lys	DBD	Germline, GOF	AD	Immune dysregulation	[10]
p.Gly421Arg	DBD	Germline, GOF	AD	Immune dysregulation	[10]
p.Asn425Lys	DBD	Germline, GOF	AD	Immune dysregulation	[10]
p.Cys426Arg	DBD	Germline, GOF	AD	Immune dysregulation	[10]
p.Thr443Ile	DBD	Germline, GOF	AD	Immune dysregulation	[10]
p.Pro471Arg	LD	Germline, GOF	AD	Immune dysregulation	[10]
p.Asp570Asn	LD	Germline, GOF	AD	Immune dysregulation	[10]
p.Glu616del	SH2	Germline, GOF	AD	Immune dysregulation	[10]
p.Glu616Val	SH2	Germline, GOF	AD	Immune dysregulation	[10]
p.Gly618Ala	SH2	Germline, GOF	AD	Immune dysregulation	[10]
p.Gln635Leu	SH2	Germline, GOF	AD	Immune dysregulation	[10]
p.Asn646Lys	SH2	Germline, GOF	AD	Immune dysregulation	[10]
p.Lys658Asn	SH2	Germline, GOF	AD	Immune dysregulation	[10]
p.Lys658Met	SH2	Germline, GOF	AD	Immune dysregulation	[10]
p.Ile659Leu	SH2	Germline, GOF	AD	Immune dysregulation	[10]
p.Thr663Ile	SH2	Germline, GOF	AD	Immune dysregulation	[10]
p.Ala703Thr	TAD	Germline, GOF	AD	Immune dysregulation	[10]
p.Pro715Leu	TAD	Germline, GOF	AD	Immune dysregulation	[10]
p.Thr716Met	TAD	Germline, GOF	AD	Immune dysregulation	[10]
p.Ser614Arg	SH2	Somatic, GOF	/	NKTCL	[11]
p.Gly618Arg	SH2	Somatic, GOF	/	NKTCL	[11]
p.Tyr640Phe	SH2	Somatic, GOF	/	NKTCL	[11–13]
p.Asp661Tyr	SH2	Somatic, GOF	/	NKTCL	[11–13]
p.Ala702Thr	SH2 (C-term)	Somatic, GOF	/	NKTCL	[11]
p.Asn647Ile	SH2	Somatic, GOF	/	T-LGLL	[12,13]
p.Tyr657_Lys658insTyr	SH2	Somatic, GOF	/	T-LGLL	[13]
p.Lys658Asn	SH2	Somatic, GOF	/	T-LGLL	[13]
p.Asp661Val	SH2	Somatic, GOF	/	T-LGLL	[12,13]
p.Asp661His	SH2	Somatic, GOF	/	T-LGLL	[13]
p.Lys658Asn	SH2	Somatic, GOF	/	T-LGLL	[12]
p.Lys658Tyr	SH2	Somatic, GOF	/	T-LGLL	[12]
p.Tyr657_Lys658insTyr	SH2	Somatic, GOF	/	T-LGLL	[12]
p.Glu616Gly	Linker-SH2	Somatic, GOF	/	T-LGLL	[12]

Dominant-negative (DN), loss of function (LOF), Autosomal Dominant (AD), Transactivation (TAD), DNA-binding (DBD), Linker Domain (LD), Coiled-coil domain (CCD), Hyper-IgE syndrome (HIES), chronic mucocutaneous candidiasis (CMC), NK/T-cell lymphoma (NKTCL), T-cell Large Granular Lymphocytic Leukemia (T-LGLL)

REFERENCES

1. M HS, R DF, Z EH, P HA, Gulbu U, Nina B, et al. STAT3 Mutations in the Hyper-IgE Syndrome. New England Journal of Medicine. Massachusetts Medical Society; 2025;357:1608–19. https://doi.org/10.1056/NEJMoa073687

2. Frede N, Rojas-Restrepo J, Caballero Garcia de Oteyza A, Buchta M, Hübscher K, Gámez-Díaz L, et al. Genetic Analysis of a Cohort of 275 Patients with Hyper-IgE Syndromes and/or Chronic

Mucocutaneous Candidiasis. J Clin Immunol. 2021;41:1804–38. https://doi.org/10.1007/s10875-021-01086-4

3. Woellner C, Gertz EM, Schäffer AA, Lagos M, Perro M, Glocker E-O, et al. Mutations in STAT3 and diagnostic guidelines for hyper-IgE syndrome. Journal of Allergy and Clinical Immunology. Elsevier; 2010;125:424-432.e8. https://doi.org/10.1016/j.jaci.2009.10.059

4. Carrabba M, Dellepiane RM, Cortesi M, Baselli LA, Soresina A, Cirillo E, et al. Long term longitudinal follow-up of an AD-HIES cohort: the impact of early diagnosis and enrollment to IPINet centers on the natural history of Job's syndrome. Allergy, Asthma & Clinical Immunology. 2023;19:32. https://doi.org/10.1186/s13223-023-00776-5

5. Lin L, Wang Y, Sun B, Liu L, Ying W, Wang W, et al. The clinical, immunological and genetic features of 12 Chinese patients with STAT3 mutations. Allergy, Asthma & Clinical Immunology. 2020;16:65. https://doi.org/10.1186/s13223-020-00462-w

6. Tavassoli M, Abolhassani H, Yazdani R, Ghadami M, Azizi G, Abdolrahim Poor Heravi S, et al. The first cohort of Iranian patients with hyper immunoglobulin E syndrome: A long-term follow-up and genetic analysis. Pediatric Allergy and Immunology. John Wiley & Sons, Ltd; 2019;30:469–78. https://doi.org/https://doi.org/10.1111/pai.13043

7. Saikia B, Rawat A, Minz RW, Suri D, Pandiarajan V, Jindal A, et al. Clinical Profile of Hyper-IgE Syndrome in India. Front Immunol . 2021;Volume 12-2021. https://doi.org/10.3389/fimmu.2021.626593

8. Wu J, Chen J, Tian Z-Q, Zhang H, Gong R-L, Chen T-X, et al. Clinical Manifestations and Genetic Analysis of 17 Patients with Autosomal Dominant Hyper-IgE Syndrome in Mainland China: New Reports and a Literature Review. J Clin Immunol. 2017;37:166–79. https://doi.org/10.1007/s10875-017-0369-7

9. Asano T, Khourieh J, Zhang P, Rapaport F, Spaan AN, Li J, et al. Human STAT3 variants underlie autosomal dominant hyper-IgE syndrome by negative dominance. Journal of Experimental Medicine. 2021;218:e20202592. https://doi.org/10.1084/jem.20202592

10. Faletti L, Ehl S, Heeg M. Germline STAT3 gain-of-function mutations in primary immunodeficiency: Impact on the cellular and clinical phenotype. Biomed J. 2021;44:412–21. https://doi.org/https://doi.org/10.1016/j.bj.2021.03.003

11. Küçük C, Jiang B, Hu X, Zhang W, Chan JKC, Xiao W, et al. Activating mutations of STAT5B and STAT3 in lymphomas derived from γδ-T or NK cells. Nat Commun. 2015;6:6025. https://doi.org/10.1038/ncomms7025

12. Savola P, Brück O, Olson T, Kelkka T, Kauppi MJ, Kovanen PE, et al. Somatic STAT3 mutations in Felty syndrome: An implication for a common pathogenesis with large granular lymphocyte leukemia. Haematologica. Ferrata Storti Foundation; 2018;103:304–12. https://doi.org/10.3324/haematol.2017.175729

13. M KHL, Samuli E, Pekka E, J van AA, Heikki K, I AE, et al. Somatic STAT3 Mutations in Large Granular Lymphocytic Leukemia. New England Journal of Medicine. Massachusetts Medical Society; 2025;366:1905–13. https://doi.org/10.1056/NEJMoa1114885

TABLE S4. Literature Reported Germline Variants in the STAT4 Gene.

N°	HGVS_P	Domain	Type	Inheritance	Associated condition	Reference
1	p.His623Tyr	SH2	Germline, GOF	Autosomal Dominant	Multisystem inflammatory disorder	[1]
2	p.Ala635Val	SH2	Germline, GOF	Autosomal Dominant	Multisystem inflammatory disorder	[1]
3	p.Ala650Asp	SH2–TAD hinge	Germline, GOF	Autosomal Dominant	Multisystem inflammatory disorder	[1]

REFERENCE

1. Hratch B, A BS, S CO, Rachael P, Brynja M, Michele N, et al. Variant STAT4 and Response to Ruxolitinib in an Autoinflammatory Syndrome. New England Journal of Medicine. Massachusetts Medical Society; 2023;388:2241–52. https://doi.org/10.1056/NEJMoa2202318

TABLE S5. Literature Reported Germline and Somatic Variants in the STAT5B gene.

HGVS_p	Protein Domain	Type of Mutation	Inheritance	Associated condition	Reference
p.Gln41Ter	N-terminal	Germinal, LOF	AR	Growth retardation, immunodeficiency	[1]
p.Arg152Ter	N-terminal	Germinal, LOF	AR	Growth retardation, immunodeficiency	[2]
p.Leu151Pro	CCD	Germinal, LOF	AR	Growth retardation, immunodeficiency	[1]
p.Ala630Pro	SH2	Germinal, LOF	AR	Growth retardation, immunodeficiency	[3]
p.Phe646Ser	SH2	Germinal, LOF	AR	Growth retardation, immunodeficiency	[1]
p.Leu142SerfsTer14	N-terminal	Germinal, LOF	AR	Growth retardation, immunodeficiency	[4]
p.His368ProfsTer12	CCD	Germinal, LOF	AR	Growth retardation, immunodeficiency	[1]
p.Lys398GluFsTer4	CCD	Germinal, LOF	AR	Growth retardation, immunodeficiency	[1]
p.Met561IlefsTer2	SH2	Germinal, LOF	AR	Growth retardation, immunodeficiency	[1]
p.Trp631Ter	SH2	Germinal, LOF	AR	Growth retardation, immunodeficiency	[5]
p.Gln177Pro	CCD	Germinal, LOF (DN)	AD	GH insensitivity syndrome (GHIS)	[6]
p.Gln474Arg	DBD	Germinal, LOF (DN)	AD	GH insensitivity syndrome (GHIS)	[6]
p.Ala478Val	DBD	Germinal, LOF (DN)	AD	GH insensitivity syndrome (GHIS)	[6]
p.Asn642His	SH2	Somatic GOF	/	Non-clonal hypereosinophilic syndrome	[7]
p.Gln706Leu	TAD	Somatic, GOF	/	T-LGL	[8]
p.Ser715Phe	TAD	Somatic, GOF	/	T-LGL	[8]
p.Asn642His	SH2	Somatic, GOF	/	T-LGL	[8]
p.Tyr665Phe	SH2	Somatic, GOF	/	T-LGL	[8]
p.Tyr665Phe	SH2	Somatic, GOF	/	T-LGL	[9]
p.Thr628Ser	SH2	Somatic, GOF	/	T-LGL	[10]
p.Pro685Arg	SH2	Somatic, GOF	/	T-LGL	[10]
p.Val712Glu	TAD	Somatic, GOF	/	T-LGL	[10]
p.Glu433Lys	DBD	Somatic, GOF	/	T-LGL	[10]
p.Glu433Gly	DBD	Somatic, GOF	/	T-LGL	[10]
p.Gln220His	CCD	Somatic, Unknown effect	/	T-LGL	[10]
p.Pro702Ala	Inter-domain	Somatic, Unknown effect	/	T-LGL	[10]
p.Glu637Lys	SH2	Somatic, GOF	/	myeloid neoplasms	[11]
p.Arg673Gln	SH2	Somatic, Unknown effect	/	myeloid neoplasms	[11]

T-cell large granular lymphocytic leukemia (T-LGL), Coiled-coil domain (CCD), DNA-binding (DBD), Transactivation domain (TAD)

REFERENCES

1. Hwa V, Nadeau K, Wit JM, Rosenfeld RG. STAT5b deficiency: Lessons from STAT5b gene mutations. Best Pract Res Clin Endocrinol Metab. 2011;25:61–75. https://doi.org/https://doi.org/10.1016/j.beem.2010.09.003

2. Nadeau K, Hwa V, Rosenfeld RG. STAT5b Deficiency: An Unsuspected Cause of Growth Failure, Immunodeficiency, and Severe Pulmonary Disease. J Pediatr. Elsevier; 2011;158:701–8. https://doi.org/10.1016/j.jpeds.2010.12.042

3. M KE, Vivian H, Brian L, A WK, K BC, Junko T, et al. Growth Hormone Insensitivity Associated with a STAT5b Mutation. New England Journal of Medicine. Massachusetts Medical Society; 2025;349:1139–47. https://doi.org/10.1056/NEJMoa022926

4. M KE, Vivian H, Brian L, A WK, K BC, Junko T, et al. Growth Hormone Insensitivity Associated with a STAT5b Mutation. New England Journal of Medicine. Massachusetts Medical Society; 2025;349:1139–47. https://doi.org/10.1056/NEJMoa022926

5. Foley CL, Al Remeithi SS, Towe CT, Dauber A, Backeljauw PF, Tyzinski L, et al. Developmental Adaptive Immune Defects Associated with STAT5B Deficiency in Three Young Siblings. J Clin Immunol. 2021;41:136–46. https://doi.org/10.1007/s10875-020-00884-6

6. Bernasconi A, Marino R, Ribas A, Rossi J, Ciaccio M, Oleastro M, et al. Characterization of Immunodeficiency in a Patient With Growth Hormone Insensitivity Secondary to a Novel STAT5b Gene Mutation. Pediatrics. 2006;118:e1584–92. https://doi.org/10.1542/peds.2005-2882

7. Ma CA, Xi L, Cauff B, DeZure A, Freeman AF, Hambleton S, et al. Somatic STAT5b gain-of-function mutations in early onset nonclonal eosinophilia, urticaria, dermatitis, and diarrhea. Blood. 2017;129:650–3. https://doi.org/10.1182/blood-2016-09-737817

8. Andersson EI, Tanahashi T, Sekiguchi N, Gasparini VR, Bortoluzzi S, Kawakami T, et al. High incidence of activating STAT5B mutations in CD4-positive T-cell large granular lymphocyte leukemia. Blood. 2016;128:2465–8. https://doi.org/10.1182/blood-2016-06-724856

9. Rajala HLM, Eldfors S, Kuusanmäki H, van Adrichem AJ, Olson T, Lagström S, et al. Discovery of somatic STAT5b mutations in large granular lymphocytic leukemia. Blood. 2013;121:4541–50. https://doi.org/10.1182/blood-2012-12-474577

10. Bhattacharya D, Teramo A, Gasparini VR, Huuhtanen J, Kim D, Theodoropoulos J, et al. Identification of novel STAT5B mutations and characterization of TCRβ signatures in CD4+ T-cell large granular lymphocyte leukemia. Blood Cancer J. 2022;12:31. https://doi.org/10.1038/s41408-022-00630-8

11. Yin CC, Tam W, Walker SM, Kaur A, Ouseph MM, Xie W, et al. STAT5B mutations in myeloid neoplasms differ by disease subtypes but characterize a subset of chronic myeloid neoplasms with eosinophilia and/or basophilia. Haematologica. Ferrata Storti Foundation; 2024;109:1825–6. https://doi.org/10.3324/haematol.2023.284311

TABLE S6. Literature Reported Germline and Somatic Variants in the STAT6 Gene.

N°	HGVS_P	Domain	Type of mutation	Inheritance	Associated condition	Reference
1	p.Glu372Lys	DBD	Germline, GOF	AD	Primary atopic disorders	[1]
2	p.Glu377Lys	DBD	Germline, GOF	AD	Primary atopic disorders	[2]
9	p.Glu382Gln	DBD	Germline, GOF	AD	Primary atopic disorders	[3]
3	p.Asp419His	DBD	Germline, GOF	AD	Primary atopic disorders	[3]
4	p.Asp419Gly	DBD	Germline, GOF	AD	Primary atopic disorders	[3]
5	p.Asp419Ala	DBD	Germline, GOF	AD	Primary atopic disorders	[3]
6	p.Asp419Tyr	DBD	Germline, GOF	AD	Primary atopic disorders	[3]
7	p.Asp419Asn	DBD	Germline, GOF	AD	Primary atopic disorders	[3]
8	p.Asp419His	DBD	Germline, GOF	AD	Primary atopic disorders	[3]
9	p.Asp519His	DBD	Germline, GOF	AD	Primary atopic disorders	[3]
10	p.Lys595Arg	DBD	Germline, GOF	AD	Primary atopic disorders	[3]
11	p.Pro643Arg	DBD	Germline, GOF	AD	Primary atopic disorders	[3]
12	p.Glu372Lys	DBD	Somatic, GOF	/	Follicular lymphoma	[4]
13	p.Glu377Lys	DBD	Somatic, GOF	/	Follicular lymphoma	[4]
14	p.Asp419His	DBD	Somatic, GOF	/	Follicular lymphoma	[4]
15	p.Asp419Ala	DBD	Somatic, GOF	/	Follicular lymphoma	[4]
16	p.Asp419Gly	DBD	Somatic, GOF	/	Follicular lymphoma	[4]
17	p.Asp419Gly; p.Pro643Leu	DBD + SH2	Somatic, GOF	/	Follicular lymphoma	[4]
18	p.Asp523Val	Linker–SH2 interface	Somatic, GOF	/	Follicular lymphoma	[4]
19	p.Asn417Tyr	DBD	Somatic, GOF	/	Primary mediastinal B-cell lymphoma	[4]
20	p.Asn417Ser	DBD	Somatic, GOF	/	Primary mediastinal B-cell lymphoma	[5]
21	p.Asp419Val	DBD	Somatic, GOF	/	Primary mediastinal B-cell lymphoma	[5]
22	p.Asn421Lys	DBD	Somatic, GOF	/	Primary mediastinal B-cell lymphoma	[5]
23	p.Asn430Thr	DBD	Somatic, GOF	/	Primary mediastinal B-cell lymphoma	[5]
24	p.Asn430Ser	DBD	Somatic, GOF	/	Primary mediastinal B-cell lymphoma	[5]
25	p.Trp444Arg	DBD	Somatic, GOF	/	Primary mediastinal B-cell lymphoma	[5]
26	p.Gln499Lys	DBD	Somatic, GOF	/	Primary mediastinal B-cell lymphoma	[5]
27	p.Asp516Ala	DBD	Somatic, GOF	/	Primary mediastinal B-cell lymphoma	[5]
28	p.Lys519Asn	DBD	Somatic, GOF	/	Primary mediastinal B-cell lymphoma	[5]
29	p.Asp419Asn	DBD	Somatic, GOF	/	Primary mediastinal B-cell lymphoma	[5]
30	p.Gln490Lys	DBD	Somatic, GOF	/	Hodgkin lymphoma	[6]

DNA-binding domain (DBD), Gain of function (GOF), Autosomal Dominant (AD)

REFERENCES

1. Baris S, Benamar M, Chen Q, Catak MC, Martínez-Blanco M, Wang M, et al. Severe allergic dysregulation due to a gain of function mutation in the transcription factor STAT6. Journal of Allergy and Clinical Immunology. Elsevier; 2023;152:182-194.e7. https://doi.org/10.1016/j.jaci.2023.01.023

2. Suratannon N, Ittiwut C, Dik WA, Ittiwut R, Meesilpavikkai K, Israsena N, et al. A germline STAT6 gain-of-function variant is associated with early-onset allergies. Journal of Allergy and Clinical Immunology. Elsevier; 2023;151:565-571.e9. https://doi.org/10.1016/j.jaci.2022.09.028

3. Sharma M, Leung D, Momenilandi M, Jones LCW, Pacillo L, James AE, et al. Human germline heterozygous gain-of-function STAT6 variants cause severe allergic disease. Journal of Experimental Medicine. 2023;220:e20221755. https://doi.org/10.1084/jem.20221755

4. Yildiz M, Li H, Bernard D, Amin NA, Ouillette P, Jones S, et al. Activating STAT6 mutations in follicular lymphoma. Blood. 2015;125:668–79. https://doi.org/10.1182/blood-2014-06-582650

5. Ritz O, Guiter C, Castellano F, Dorsch K, Melzner J, Jais J-P, et al. Recurrent mutations of the STAT6 DNA binding domain in primary mediastinal B-cell lymphoma. Blood. 2009;114:1236–42. https://doi.org/10.1182/blood-2009-03-209759

6. Tiacci E, Ladewig E, Schiavoni G, Penson A, Fortini E, Pettirossi V, et al. Pervasive mutations of JAK-STAT pathway genes in classical Hodgkin lymphoma. Blood. 2018;131:2454–65. https://doi.org/10.1182/blood-2017-11-814913

TABLE S7. Literature Reported Germline Variants in the JAK1 Gene.

HGVS_p	Protein domain	Type of mutation	Inheritance	Associated condition	Reference
p.His596Asp	Pseudokinase (JH2)	Germline, GOF	AD	Autoinflammatory keratinization disease with hepatitis et autism	[1]
p.Ile597Phe	Pseudokinase (JH2)	Germline, GOF	AD	Autoinflammatory syndrome with hypereosinophilia	[2]
p.Ala634Asp	Pseudokinase (JH2)	Germline, GOF	AD	Complex autoinflammatory syndrome	[3]
p.Ser703Ile	Pseudokinase (JH2)	Germline, GOF	AD	Mosaic: autoinflammation + atopy + multi-organ immune dysregulation	[3]
p.Cys787Phe	pseudokinase (JH2)	Germline, GOF	AD	Severe early-onset inflammatory disease	[4]
p.Glu139Lys	FERM Domain	Germline, GOF	AD	Inflammatory immune dysregulation and atopy	[4]
p.Arg506Cys	SH2 Domain	Germline, GOF	AD	Cytokine-dependent hyperactivation; atopy and chronic inflammation	[4]
p.Ser700Asn	pseudokinase (JH2)	Germline, GOF	AD	mixed immune disorders	[4]
p.(Val985Ile)	Kinase domain (JH1)	Germline, GOF	AD	Systemic inflammation and atopy	[4]
p.Val464Met	Pseudokinase (JH2)	Germline, GOF	AD	Cutaneous manifestations/eczema	[5]
p.Pro815Ser	Pseudokinase (JH2)	Germline, GOF	AD	Systemic autoimmune/inflammatory features	[5]
p.Arg879Gly	Kinase domain (JH1)	Germline, GOF	AD	Recurrent bacterial/viral infections	[5]
p.Asn917Asp	Kinase domain (JH1)	Germline, GOF	AD	Immune dysregulation	[5]
p.Asp1042Tyr	Kinase domain (JH1)	Germline, GOF	AD	dermatologic disease	[5]
p.Pro733Leu	Pseudokinase (JH2)	Germline, LOF	AR	MSMD	[6]
p.Pro832Ser	Pseudokinase (JH2)	Germline, LOF	AR	MSMD	[6]

N-terminal domain (NTD), Coiled-coil domain (CCD), Tail segment domain (TSD), DNA-binding domain (DBD), Transactivation domain (TAD), SH2 domain (SH2D), Linker domain (L), Chronic mucocutaneous candidiasis (CMC); Mendelian susceptibility to mycobacterial disease (MSMD)

REFERENCES

1. Takeichi T, Lee JYW, Okuno Y, Miyasaka Y, Murase Y, Yoshikawa T, et al. Autoinflammatory Keratinization Disease With Hepatitis and Autism Reveals Roles for JAK1 Kinase Hyperactivity in Autoinflammation. Front Immunol. 2022;Volume 12-2021. https://doi.org/10.3389/fimmu.2021.737747

2. Del Bel KL, Ragotte RJ, Saferali A, Lee S, Vercauteren SM, Mostafavi SA, et al. JAK1 gain-of-function causes an autosomal dominant immune dysregulatory and hypereosinophilic syndrome. Journal of Allergy and Clinical Immunology. Elsevier; 2017;139:2016-2020.e5. https://doi.org/10.1016/j.jaci.2016.12.957

3. Gruber CN, Calis JJA, Buta S, Evrony G, Martin JC, Uhl SA, et al. Complex Autoinflammatory Syndrome Unveils Fundamental Principles of JAK1 Kinase Transcriptional and Biochemical Function. Immunity. Elsevier; 2020;53:672-684.e11. https://doi.org/10.1016/j.immuni.2020.07.006

4. Horesh ME, Martin-Fernandez M, Gruber C, Buta S, Le Voyer T, Puzenat E, et al. Individuals with JAK1 variants are affected by syndromic features encompassing autoimmunity, atopy, colitis, and dermatitis. Journal of Experimental Medicine. 2024;221:e20232387. https://doi.org/10.1084/jem.20232387

5. Jeanpierre M, Debeaupuis O, Brunaud C, Yancoski J, Riller Q, Hadjadj J, et al. In silico modeling guides identification of novel *JAK1* variants associated with immune dysregulation. EMBO Mol Med. Springer Nature; 2025;1-25–25. https://doi.org/https://doi.org/10.1038/s44321-025-00317-0

6. Eletto D, Burns SO, Angulo I, Plagnol V, Gilmour KC, Henriquez F, et al. Biallelic JAK1 mutations in immunodeficient patient with mycobacterial infection. Nat Commun. 2016;7:13992. https://doi.org/10.1038/ncomms13992

TABLE S8. Literature Reported Germline and Somatic Variants in the JAK2 Gene.

HGVS_p	Domain	Type of mutation	Associated Condition	Reference
p.Val617Phe	Pseudokinase (JH2)	Somatic, GOF	Polycythemia vera, essential thrombocythemia, primary myelofibrosis	[1]
p.Lys539Leu	JH2/JH1 interface	Somatic, GOF	Polycythemia vera (V617F–negative)	[2]
p.Cys616Tyr	Pseudokinase (JH2)	Somatic, GOF	MPN	[3]
p.Arg564Gln	Pseudokinase (JH2)	Germline, GOF	Familial thrombocythemia / hereditary MPN	[4,5]
p.Arg564Leu	Pseudokinase (JH2)	Germline, GOF	Familial thrombocythemia	[6]
p.Phe556Val	Pseudokinase / regulatory loop	Germline, GOF	Hereditary thrombocytosis	[7]
p.Glu846Asp	Kinase (JH1)	Germline, Likely GOF	Familial erythrocytosis	
p.Arg1063His	C-terminal kinase	Germline, Uncertain / mild GOF	Familial erythrocytosis / MPN predisposition	[8]
p.Val617Ile	Pseudokinase (JH2)	Germline, GOF (weaker than V617F)	Familial MPN (rare)	[9]
p.Ile223Thr	FERM	Germline, Possible GOF	Hereditary thrombocytosis	[10]
p.His608Asn	Regulatory region (JH2 proximal)	Germline	Familial MPN	[11]
p.Ile724Thr	Kinase (JH1)	Germline	Reported in variant reviews	[12]
p.Gly571Ser	Pseudokinase (JH2)	Somatic / Germline GOF (weak)	MPN / AML occasional	[13]
p.Val625Phe	Pseudokinase (JH2)	Somatic, GOF	MPN / AML cohorts	[14]

Gain of function (GOF), Acute Myeloid Leukemia (AML), Myeloproliferative Neoplasms (MPN)

REFERENCES

1. Robert K, Francesco P, S BA, Soon-Siong T, Ralph T, R PJ, et al. A Gain-of-Function Mutation of JAK2 in Myeloproliferative Disorders. New England Journal of Medicine. Massachusetts Medical Society; 2025;352:1779–90. https://doi.org/10.1056/NEJMoa051113

2. M SL, Wei T, L LR, A SM, A BP, R SM, et al. JAK2 Exon 12 Mutations in Polycythemia Vera and Idiopathic Erythrocytosis. New England Journal of Medicine. Massachusetts Medical Society; 2025;356:459–68. https://doi.org/10.1056/NEJMoa065202

3. Zhang S-J, Li J-Y, Li W-D, Song J-H, Xu W, Qiu H-X. The investigation of JAK2 mutation in Chinese myeloproliferative diseases-identification of a novel C616Y point mutation in a PV patient. Int J Lab Hematol. John Wiley & Sons, Ltd; 2007;29:71–2. https://doi.org/https://doi.org/10.1111/j.1365-2257.2006.00864.x

4. Stewart BL, Carraway HE, Alvarez AL, Lesmana H, Molina J, Tu ZJ, et al. JAK2 p.R564 germ line variants associated with hereditary thrombocythemia and hematologic neoplasms. Blood Adv. 2025;9:1534–43. https://doi.org/10.1182/bloodadvances.2024013661

5. Franco S, Krawiec K, Strzalka P, Godley LA. Germline JAK2 R564Q variants presenting as hereditary thrombocytosis: case report. BJC Reports. 2025;3:69. https://doi.org/10.1038/s44276-025-00186-7

6. Cannova J, Drazer MW. Germ line JAK2 variants and hereditary blood cancers. Blood Adv. 2025;9:1585–6. https://doi.org/10.1182/bloodadvances.2025015810

7. Meggendorfer M, Haferlach T, Beykirch MK, Petrides PE. Unraveling the germline inheritance of the F556V gene mutation in familial thrombocythemia: a comprehensive analysis of 11 family members and potential implications for surveillance. Haematologica. 2025;110:485–7. https://doi.org/10.3324/haematol.2024.285329

8. Zimolova V, Buroczíova M, Berkova L, Grusanovic S, Gursky J, Janotka L, et al. Germline Jak2-R1063H mutation interferes with normal hematopoietic development and increases risk of thrombosis and leukemic transformation. Leukemia. 2025; https://doi.org/10.1038/s41375-025-02737-w

9. J MA, J RM, W JSE, Anna S. Germline JAK2 Mutation in a Family with Hereditary Thrombocytosis. New England Journal of Medicine. Massachusetts Medical Society; 2025;366:967–9. https://doi.org/10.1056/NEJMc1200349

10. Müller J, Porret NA, Rüfer A. Identification of a JAK2 FERM Domain Variant Associated With Hereditary Thrombocytosis. Hemasphere. 2021;5(8):e626. https://doi.org/10.1097/HS9.0000000000000626

11. Maaziz N, Garrec C, Airaud F, Bobée V, Contentin N, Cayssials E, et al. Germline JAK2 E846D Substitution as the Cause of Erythrocytosis? Genes (Basel). MDPI; 2023;14. https://doi.org/10.3390/genes14051066

12. Puli'uvea C, Immanuel T, Green TN, Tsai P, Shepherd PR, Kalev-Zylinska ML. Insights into the role of JAK2-I724T variant in myeloproliferative neoplasms from a unique cohort of New Zealand patients. Hematology. Taylor & Francis; 2024;29:2297597. https://doi.org/10.1080/16078454.2023.2297597

13. Panovska-Stavridis I, Eftimov A, Pivkova-Veljanovska A, Ivanovski M, Cevreska L, Dimovski AJ. Familiar JAK2 G571S Variant Not Linked with Essential Trombocythemia. Blood. 2014;124:5585. https://doi.org/10.1182/blood.V124.21.5585.5585

14. Feenstra JDM, Nivarthi H, Gisslinger H, Leroy E, Rumi E, Chachoua I, et al. Whole-exome sequencing identifies novel MPL and JAK2 mutations in triple-negative myeloproliferative neoplasms. 2016; https://doi.org/10.1182/blood-2015

TABLE S9. Literature Reported Germline and Somatic Variants in the JAK3 Gene.

HGVS_p	Protein domain	Mutation type	Inheritance	Associated condition	Reference
p.Met1Val	FERM	Germline, LOF	AR	SCID	[1]
p.Gly36fs*146	FERM	Germline, LOF	AR	SCID	[1]
p.Ala58del	FERM	Germline, LOF	AR	SCID	[1]
p.Ala58Pro	FERM	Germline, LOF	AR	SCID	[1]
p.Tyr100Cys	FERM	Germline, LOF	AR	SCID	[1]
p.Pro151Arg	FERM	Germline, LOF	AR	SCID	[1]
p.Asp169Glu	FERM	Germline, LOF	AR	SCID	[1]
p.Gln286*	Pseudokinase	Germline, LOF	AR	SCID	[1]
p.Gln286insfs*302	Pseudokinase	Germline, LOF	AR	SCID	[1]
p.Thr391fs*408	Pseudokinase	Germline, LOF	AR	SCID	[1]
p.Arg445*	Pseudokinase	Germline, LOF	AR	SCID	[1]
p.Leu462insfs*519	Pseudokinase	Germline, LOF	AR	SCID	[1]
p.Glu481Gly	Pseudokinase	Germline, LOF	AR	SCID	[1]
p.Glu481_Asp482del	Pseudokinase	Germline, LOF	AR	SCID	[1]
p.Lys482_Ser596del	JH4–JH3	Germline, LOF	AR	SCID	[1]
p.Cys565*	Pseudokinase	Germline, LOF	AR	SCID	[1]
p.Arg582Trp	Pseudokinase	Germline, LOF	AR	SCID	[1]
p.Gly589Ser	Pseudokinase	Germline, LOF	AR	SCID	[1]
p.Val590_Ser596del	Pseudokinase	Germline, LOF	AR	SCID	[1]
p.Arg651Trp	Pseudokinase	Germline, LOF	AR	SCID	[1]
p.Pro689Ser	Pseudokinase	Germline, LOF	AR	SCID	[1]
p.Glu694Lys	Pseudokinase	Germline, LOF	AR	SCID	[1]
p.Glu698*	Pseudokinase	Germline, LOF	AR	SCID	[1]
p.Lys734_Asp784del	Pseudokinase	Germline, LOF	AR	SCID	[1]
p.Gln766*	Pseudokinase	Germline, LOF	AR	SCID	[1]
p.Arg771*	Pseudokinase	Germline, LOF	AR	SCID	[1]
p.Asp784Asn	Pseudokinase	Germline, LOF	AR	SCID	[1,2]
p.Tyr904*	Kinase	Germline, LOF	AR	SCID	[1]
p.Leu910Ser	Kinase	Germline, LOF	AR	SCID	[1]
p.Tyr929*	Kinase	Germline, LOF	AR	SCID	[1]
p.Gly987fs*1031	Kinase	Germline, LOF	AR	SCID	[1]
p.Tyr1023*	Kinase	Germline, LOF	AR	SCID	[1]
p.Cys1024fs*1037	Kinase	Germline, LOF	AR	SCID	[1]
p.Leucine156Proline	FERM	Somatic, GOF	/	T/NK-cell neoplasm	[3]
p.Aspartic acid172Glutamine	FERM	Somatic, GOF	/	T/NK-cell neoplasm	[3,4]
p.Leucine183Glycine	FERM	Somatic, GOF	/	T/NK-cell neoplasm	[3,4]
p.Glutamine507Proline	SH2	Somatic, GOF	/	T/NK-cell neoplasm	[4,5]
p.Methionine511Isoleucine	SH2	Somatic, GOF	/	T/NK-cell neoplasm	[4,6]
p.Alanine518Histidine	SH2	Somatic, GOF	/	T/NK-cell neoplasm	[4]
p.Alanine572Valine	Pseudokinase	Somatic, GOF	/	T/NK-cell neoplasm	[4]
p.Alanine573Valine	Pseudokinase	Somatic, GOF	/	T/NK-cell neoplasm	[4,6]
p.Arginine657Glutamine	Pseudokinase	Somatic, GOF	/	T/NK-cell neoplasm	[4,6]
p.Valine674Alanine	Pseudokinase	Somatic, GOF	/	T/NK-cell neoplasm	[4,6]
p.Valine674Phenylalanine	Pseudokinase	Somatic, GOF	/	T/NK-cell neoplasm	[6]
p.Valine678Leucine	Pseudokinase	Somatic, GOF	/	T/NK-cell neoplasm	[6]
p.Gln988Pro	Kinase	Somatic, GOF	/	T-cell acute lymphoblastic leukemia	[7]

Gain of function (GOF), Loss of function (LOF), Severe combined immunodeficiency (SCID)

REFERENCES

1. Liongue C, Ratnayake T, Basheer F, Ward AC. Janus Kinase 3 (JAK3): A Critical Conserved Node in Immunity Disrupted in Immune Cell Cancer and Immunodeficiency. Int J Mol Sci. 2024;25. https://doi.org/10.3390/ijms25052977

2. Barreiros LA, Segundo GRS, Grumach AS, Roxo-Júnior P, Torgerson TR, Ochs HD, et al. A Novel Homozygous JAK3 Mutation Leading to T-B+NK– SCID in Two Brazilian Patients. Front Pediatr. 2018;Volume 6-2018. https://doi.org/10.3389/fped.2018.00230

3. Elliott NE, Cleveland SM, Grann V, Janik J, Waldmann TA, Davé UP. FERM domain mutations induce gain of function in JAK3 in adult T-cell leukemia/lymphoma. Blood. 2011;118:3911–21. https://doi.org/10.1182/blood-2010-12-319467

4. Bodaar K, Yamagata N, Barthe A, Landrigan J, Chonghaile TN, Burns M, et al. JAK3 mutations and mitochondrial apoptosis resistance in T-cell acute lymphoblastic leukemia. Leukemia. 2022;36:1499–507. https://doi.org/10.1038/s41375-022-01558-5

5. Kiel MJ, Velusamy T, Rolland D, Sahasrabuddhe AA, Chung F, Bailey NG, et al. Integrated genomic sequencing reveals mutational landscape of T-cell prolymphocytic leukemia. Blood. 2014;124:1460–72. https://doi.org/10.1182/blood-2014-03-559542

6. Degryse S, de Bock CE, Cox L, Demeyer S, Gielen O, Mentens N, et al. JAK3 mutants transform hematopoietic cells through JAK1 activation, causing T-cell acute lymphoblastic leukemia in a mouse model. Blood. 2014;124:3092–100. https://doi.org/10.1182/blood-2014-04-566687

7. Lahera A, Vela-Martín L, Fernández-Navarro P, Llamas P, López-Lorenzo JL, Cornago J, et al. The JAK3Q988P mutation reveals oncogenic potential and resistance to ruxolitinib. Mol Carcinog. John Wiley & Sons, Ltd; 2024;63:5–10. https://doi.org/https://doi.org/10.1002/mc.23632

TABLE S10. Literature Reported Germline and Somatic Variants in the TYK2 Gene.

HGVS_p	Protein domain	Effect	Inheritance	Phenotype	Reference
**p.Pro1104Ala**	Kinase (JH1)	Germline, LOF (Partial)	AR	MSMD	[1]
**p.Cys70Serfs*21**	FERM (N-term)	Germline, LOF (Complete)	AR (homozygous / compound het)	MSMD	[2]
**p.Ser50Hisfs*1**	FERM	Germline, LOF (Complete)	AR (homozygous)	MSMD	[3]
**p.Glu154***	FERM	Germline, LOF (Complete)	AR (homozygous)	MSMD	[3]
**p.Arg638***	Pseudokinase (JH2)	Germline, LOF (Complete)	AR (homozygous)	MSMD	[3]
**premature stop**	Pseudokinase (JH2)	Germline, LOF (Complete)	AR (homozygous)	MSMD	[3]
**premature stop**	Kinase (JH1)	Germline, LOF (Complete)	AR (homozygous)	MSMD	[3]
**p.Gly799Arg**	Pseudokinase (JH2)	Germline, LOF	AR (homozygous)	MSMD	[4]
**p.Arg231Trp**	SH2-like / near FERM	Germline, LOF (Partial)	AR (compound heterozygous)	CID / lymphoma	[5]
**p.Gly634Glu**	Pseudokinase (JH2)	Germline, LOF (Partial)	AR (homozygous)	MSMD	[6]
**p.Arg864Cys**	Pseudokinase (JH2)	Germline, LOF (Partial)	AR (homozygous)	MSMD	[6]
**p.Gly996Arg**	JH2–JH1 interface	Germline, LOF (Partial)	AR (homozygous)	MSMD	[6]
**p.Gly1010Asp**	Kinase (JH1)	Germline, LOF	AR (homozygous)	MSMD	[6]
**p.Arg425His**	FERM (JH4)	Somatic, LOF	/	B-ALL	[7]
**p.Ser431Gly**	FERM (JH4)	Somatic, LOF	/	B-ALL	[7]
**p.Arg832Trp**	Pseudokinase (JH2)	Somatic, LOF	/	B-ALL	[7]
**p.Pro760Leu**	Pseudokinase (JH2)	Somatic, GOF	/	Pediatric ALL	[8]
**p.Gly761Val**	Pseudokinase (JH2)	Somatic, GOF	/	Pediatric ALL	[8]

Autosomal recessive (AR), B-cell acute lymphoblastic leukemia (B-ALL), Loss of function (LOF), combined immunodeficiency (CID), Mendelian Susceptibility to Mycobacterial Disease (MSMD), Hyper-IgE syndrome (HIES)

REFERENCES

1. Kerner G, Ramirez-Alejo N, Seeleuthner Y, Yang R, Ogishi M, Cobat A, et al. Homozygosity for TYK2 P1104A underlies tuberculosis in about 1% of patients in a cohort of European ancestry. Proceedings of the National Academy of Sciences. Proceedings of the National Academy of Sciences; 2019;116:10430–4. https://doi.org/10.1073/pnas.1903561116

2. Minegishi Y, Saito M, Morio T, Watanabe K, Agematsu K, Tsuchiya S, et al. Human Tyrosine Kinase 2 Deficiency Reveals Its Requisite Roles in Multiple Cytokine Signals Involved in Innate and Acquired Immunity. Immunity. Elsevier; 2006;25:745–55. https://doi.org/10.1016/j.immuni.2006.09.009

3. Kreins AY, Ciancanelli MJ, Okada S, Kong X-F, Ramírez-Alejo N, Kilic SS, et al. Human TYK2 deficiency: Mycobacterial and viral infections without hyper-IgE syndrome. Journal of Experimental Medicine. 2015;212:1641–62. https://doi.org/10.1084/jem.20140280

4. Wu P, Chen S, Wu B, Chen J, Lv G. A TYK2 Gene Mutation c.2395G>A Leads to TYK2 Deficiency: A Case Report and Literature Review. Front Pediatr. 2020;Volume 8-2020. https://doi.org/10.3389/fped.2020.00253

5. Nemoto M, Hattori H, Maeda N, Akita N, Muramatsu H, Moritani S, et al. Compound heterozygous TYK2 mutations underline primary immunodeficiency with T-cell lymphopenia. Sci Rep. 2018;8:6956. https://doi.org/10.1038/s41598-018-25260-8

6. Ogishi M, Arias AA, Yang R, Han JE, Zhang P, Rinchai D, et al. Impaired IL-23–dependent induction of IFN-γ underlies mycobacterial disease in patients with inherited TYK2 deficiency. Journal of Experimental Medicine. 2022;219:e20220094. https://doi.org/10.1084/jem.20220094

7. Turrubiartes-Martínez E, Bodega-Mayor I, Delgado-Wicke P, Molina-Jiménez F, Casique-Aguirre D, González-Andrade M, et al. Tyk2 variants in b-acute lymphoblastic leukaemia. Genes (Basel). MDPI AG; 2020;11:1–16. https://doi.org/10.3390/genes11121434

8. Woess K, Macho-Maschler S, van Ingen Schenau DS, Butler M, Lassnig C, Valcanover D, et al. Oncogenic TYK2 P760L kinase is effectively targeted by combinatorial TYK2, mTOR and CDK4/6 kinase blockade. Haematologica. Ferrata Storti Foundation; 2023;108:993–1005. https://doi.org/10.3324/haematol.2021.279848

TABLE S11. Newborn Screening Outcomes and Diagnostic Pathways in JAK–STAT–Related Immunodeficiencies

Gene	Typical phenotype (LOF / GOF)	Expected TREC/KREC	Likelihood of detection by NBS
JAK3	LOF → SCID ($T^-B^+NK^-$)	Very low TREC	Detected. Classic SCID target of TREC screening.
STAT1 (GOF)	Immune dysregulation, CMC, autoimmunity	Normal TREC/KREC	Usually missed (functional/regulatory defect).
STAT1 (LOF)	Mendelian susceptibility to mycobacteria/viruses	Normal TREC/KREC	Usually missed.
STAT2	Severe viral susceptibility (type I IFN pathway)	Normal TREC/KREC	Usually missed (innate antiviral signalling defect).
STAT3 (GOF)	Autoimmunity, lymphoproliferation	Normal TREC/KREC	Usually missed (numbers often preserved).
STAT3 (LOF)	AD-HIES (Hyper-IgE syndrome)	Normal TREC/KREC	Usually missed.
STAT4	Impaired Th1/IL-12 responses (rare)	Normal TREC/KREC	Usually missed.
STAT5B	T-cell growth signalling defect; GH insensitivity	Normal/near-normal TREC	Usually missed (functional defect).
STAT6	Th2/atopy dysregulation (GOF recently reported)	Normal TREC/KREC	Usually missed.
JAK1	Cytokine signalling defects (germline LOF rare)	Normal TREC/KREC	Usually missed.
JAK2	Germline very rare; somatic GOF → MPNs	Normal TREC/KREC	Usually missed (not a congenital T/B output defect).
TYK2	Impaired IFN/IL-12/23 signalling; mycobacterial/viral susceptibility	Normal/near-normal TREC/KREC	Usually missed (signalling defect).
